# Towards Solar Methanol: Past, Present, and Future

**DOI:** 10.1002/advs.201801903

**Published:** 2019-02-19

**Authors:** Athanasios A. Tountas, Xinyue Peng, Alexandra V. Tavasoli, Paul N. Duchesne, Thomas L. Dingle, Yuchan Dong, Lourdes Hurtado, Abhinav Mohan, Wei Sun, Ulrich Ulmer, Lu Wang, Thomas E. Wood, Christos T. Maravelias, Mohini M. Sain, Geoffrey A. Ozin

**Affiliations:** ^1^ Department of Chemical Engineering and Applied Chemistry University of Toronto 200 College Street Toronto ON M5S 3E5 Canada; ^2^ Department of Chemical and Biological Engineering University of Wisconsin–Madison 1415 Engineering Drive Madison WI 53706 USA; ^3^ Department of Materials Science and Engineering University of Toronto 184 College St Toronto ON M5S 3E4 Canada; ^4^ Department of Chemistry University of Toronto 80 St. George Street Toronto Ontario M5S 3H6 Canada; ^5^ Department of Mechanical and Industrial Engineering University of Toronto 5 King's College Road Toronto ON M5S 3G8 Canada

**Keywords:** commercial methanol production, solar‐assisted processes, solar methanol, solar reactor design and engineering, technoeconomic analysis

## Abstract

This work aims to provide an overview of producing value‐added products affordably and sustainably from greenhouse gases (GHGs). Methanol (MeOH) is one such product, and is one of the most widely used chemicals, employed as a feedstock for ≈30% of industrial chemicals. The starting materials are analogous to those feeding natural processes: water, CO_2_, and light. Innovative technologies from this effort have global significance, as they allow GHG recycling, while providing society with a renewable carbon feedstock. Light, in the form of solar energy, assists the production process in some capacity. Various solar strategies of continually increasing technology readiness levels are compared to the commercial MeOH process, which uses a syngas feed derived from natural gas. These strategies include several key technologies, including solar‐thermochemical, photochemical, and photovoltaic–electrochemical. Other solar‐assisted technologies that are not yet commercial‐ready are also discussed. The commercial‐ready technologies are compared using a technoeconomic analysis, and the scalability of solar reactors is also discussed in the context of light‐incorporating catalyst architectures and designs. Finally, how MeOH compares against other prospective products is briefly discussed, as well as the viability of the most promising solar MeOH strategy in an international context.

## 1 Methanol: Then and Now

Methanol (MeOH) has a long and fascinating history. Following its first recorded use in Ancient Egypt for embalming rituals, it remained unisolated in its pure form until the necessary experiments were performed by Sir Robert Boyle in 1661. It was first produced at the industrial scale in 1923 from synthesis gas or “syngas,” a mixture of carbon monoxide (CO) and hydrogen (H_2_) derived from coal, thanks to the work of Alwin Mittasch and Mathias Pier at BASF.^1^ These inventors filed the first patent on the synthesis of MeOH from syngas in 1913.^1^ Today, more than 90 production plants are in operation worldwide, with a combined production capacity of around 110 million metric tonnes per year (MMTA), while providing upward of 90 000 jobs and creating roughly US$55 billion in economic activity.^2^

Major demands for MeOH originate from its growing use as both a chemical feedstock and a petroleum fuel substitute. As an important feedstock for the chemical industry, it is used in the production of as much as 30% of global industrial chemicals.^3^, ^4^ It can be used to produce olefins, formaldehyde, acetic acid, and diverse products such as building materials, pharmaceuticals, resins, adhesives, paints, plastics, and foams.^5^, ^6^ The development of renewable, environmentally friendlier fuels is also the focus of many researchers worldwide. As urban populations continue to experience deteriorating air quality, there has been increasing pressure on governments and the transportation sector to find drop‐in, cleaner‐burning fuels to replace fossil fuels. To this end, dimethyl ether (DME) and its less volatile higher homologues, polyoxymethylene dimethyl ethers (PODEs), have attracted increasing attention for their ability to produce less soot when burned in a standard diesel engine. Both of these compounds can be produced, in part, from MeOH. Furthermore, MeOH has been shown to improve internal combustion engine efficiency and performance via its use as a gasoline additive or as a neat fuel.^7^ As will be shown later, MeOH can be produced as efficiently as DME from “solar‐accessible” syngas, and is more appealing due to its many commercial applications. Furthermore, MeOH can be used in fuel cells as an alternative to H_2_, due to its higher volumetric energy density, and also serves as a medium for the safe storage of H_2_ (i.e., as a H_2_ “shuttle”), since each MeOH molecule can fixate four H atoms. Both of these efforts have resulted in an increased focus on the pursuit for sustainable MeOH.^8^

The current dominant industrial feedstock for MeOH synthesis is syngas from natural gas (NG) representing 75% of the market (other sources include coal, biomass, and CO_2_).^9^ The syngas, besides containing a mixture of CO and H_2_, also contains a small quantity of added CO_2_. The original BASF process involved the gas‐phase heterogeneous catalytic conversion of syngas to MeOH at high temperatures and pressures of 360–380 °C and 25–30 MPa, respectively, using a chromium and manganese oxide–based catalyst.^10^ This process afforded a relatively low yield of just 10 mol% MeOH.^10^ Today, the preferred catalysts for manufacturing MeOH are mainly copper–zinc oxide hetero‐nanostructures supported on aluminum oxide (CZA); these catalysts function more productively, but are able to do so under much milder conditions of temperature and pressure, typically 200–300 °C and 5–10 MPa, respectively.

The commercial CZA catalyst in use today is the result of six decades of continuous improvement and optimization. Originally patented by John Thomas Gallagher and John Mitchell Kidd of Imperial Chemical Industries (ICI) in 1965,^11^ the technology was acquired by Johnson Matthey through the purchase of ICI's Synetix catalyst business. Synetix specialized in delivering catalysts to the MeOH, ammonia, oil and gas, chemicals, fine chemicals, and oleochemical industries, among others.^12^ The original catalyst patent is still the basis of the KATALCO 51‐series catalyst sold by Johnson Matthey today and is the basis of the kinetics model used here to predict the CZA catalyst MeOH productivity from a standard CO‐rich feed (as described in Section 2.3) and from increasingly CO_2_‐rich feeds (as described in Section 4.3.1). It is envisioned that this catalyst will find continued use due to its low cost, high activity, and stability when used in conjunction with CO‐rich feeds.

Due in part to rising concern about anthropogenic CO_2_ emissions and the unsustainable sourcing of syngas from NG, there has recently been increasing emphasis on using CO_2_ and H_2_O (water) in place of NG as the major feedstocks for MeOH production. This would allow CO_2_, the most abundant greenhouse gas (GHG), to be recycled into useful chemicals and products, thereby helping to “close” the carbon cycle. Low‐temperature, highly active, economical, and water‐stable catalysts are currently being sought due to the more favorable equilibrium yields with CO_2_‐rich feeds at low temperatures. Alternatively, harnessing the already productive CZA catalyst by adding a quantity of renewably CO_2_‐derived CO to the feed could be more economically feasible near term with minimal investment.

**Figure 1:**
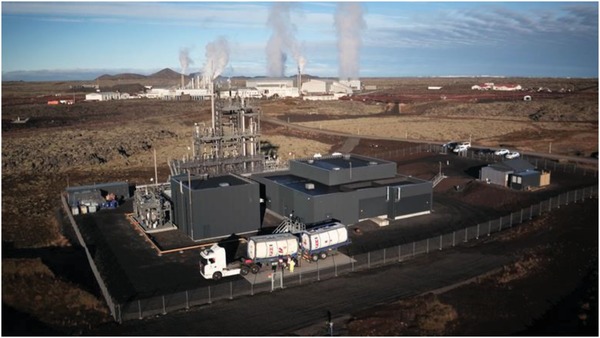
Carbon recycling international CO_2_‐to‐MeOH plant, Reykjavik, Iceland. (Image courtesy of CRI).

Nobel laureate George Olah has been a strong proponent of the vision of the “MeOH economy,”^1^ which is exemplified in the form of a renewable MeOH production plant in Reykjavik, Iceland (**Figure** **1**). This industrial facility commissioned in 2007, and named in his honor, annually produces 4000 metric tonnes of MeOH (MT_MeOH_) made from CO_2_ and H_2_. This corresponds to 5500 MT_CO2_ recycled per year. The location of the facility allows it to utilize geothermal steam from the 75 MWel (0.18 MT_CO2_ MWh^−1^) HS Orka's Svartsengi power station (shown in background of figure) to provide heat (for distillation) and electricity (5 MWel) without burning fossil fuels. It uses ≈10% of the total CO_2_ emission of the power station. This electricity is used for high pressure (≈3 MPa) alkaline water electrolysis to produce H_2_, which in turn reduces CO_2_ to MeOH in the presence of a CZA catalyst, in a process operating at 250 °C and 5–10 MPa.^13^

### 1.1 Solar MeOH

An innovative trend becoming increasingly evident in the scientific literature is the use of light to drive or assist chemical reactions and processes. A solar‐assisted process is defined as one where a portion of the total energy required for the process is replaced by solar energy. The prospect of using solar energy and feedstocks CO_2_ and water to synthesize MeOH could lead to an economically viable technology capable of replacing the fossil fuel‐heavy industry with a renewably sourced alternative. One prospect is that solar energy could help unlock favorable lower‐temperature equilibrium regimes. Otherwise, it provides a vast source (energy intercepted by the Earth in 1 h > annual world consumption) of clean energy that can be harnessed in various ways to produce light‐assisted chemical products.^14^

**Figure 2:**
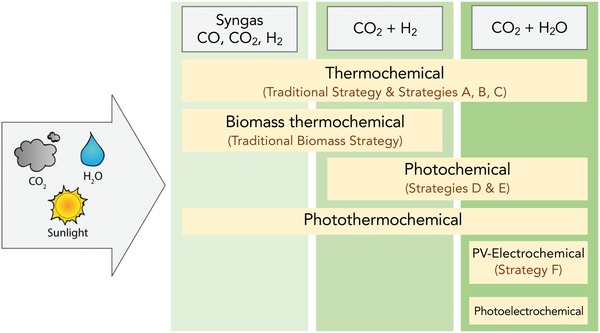
Traditional and solar methanol production technologies. Technologies can be used with feedstocks shown in gray. All technologies discussed in this review are shown, including the applicable feedstocks derived from the raw materials that can be used as feed. The two traditional strategies are for thermochemical conversion of natural gas and biomass to MeOH. The strategies listed as A–F utilize solar energy in some capacity either via solar‐thermochemical, photochemical, photothermal, and photoelectrochemical processes or electricity derived from light, the definitions of which are provided in Section 1.2. The strategies will be expanded upon in Section 1.4.

Depicted in **Figure** **2** are the various current pathways to solar MeOH. Ideally, light can be used directly in the process to convert feedstocks CO_2_ and water through solar thermochemistry, photochemistry, or photoelectrochemistry. Light energy can also be incorporated indirectly by first converting it to electricity, using photovoltaic (PV) materials, or heat, via photothermal materials; this electricity and heat could then be used to drive CO_2_ conversion through electrochemistry, photothermochemistry, or traditional thermal catalysts. Direct and indirect biological approaches also exist that could enable the incorporation of light into MeOH synthesis, primarily involving genetically modified organisms and hybrid bioinorganic systems, respectively. However, these remain at the lab scale. Refs. 15, 16, 17, 18, 19, 20 are recommended for more information. Given the various approaches, the viability of each traditional and solar MeOH technology strategy will be evaluated and compared from a technoeconomic analysis (TEA) perspective. The TEA plant capacities are for a production rate of 1 kg_MeOH_ s^−1^. Comparison will focus on energy efficiency and process economics performance metrics that are resulting from the activity, selectivity, yield, energy efficiency, scalability, and relative cost of each process. The most promising strategy based on the TEA will also be compared to the traditional commercial‐scale thermochemical (TCST) MeOH synthesis process from an environmental perspective.

### 1.2 Definition of Technologies

In order to improve accessibility to a broad readership, it is helpful here to clarify some commonly used terminology. In this review, we use the following terms to describe the various routes by which feedstocks CO_2_, water, and light (or NG/biomass and water for the traditional strategies) can be converted into MeOH:

- “Thermochemistry” refers to a technology where the primary activation energy to drive the reaction is a result of thermal (heat) energy input into the process. This can also include “solar thermochemistry” in which solar heat is used to provide the heat of reaction for conversion of CO_2_ and water into syngas.
- “Photochemistry” refers to a technology in which light is absorbed by a photocatalyst to generate excited electronic states and activate the desired reaction. In the case of a semiconductor photocatalyst, the incident photon must have energy greater than the bandgap of the material.
- “Photothermochemistry” refers to a technology in which absorbed light generates heat (typically generated via nonradiative electronic relaxations) locally on the surface of the catalyst, whereupon the reaction is activated thermally.
- “Electrochemistry” or “PV–electrochemistry” refers to a technology where the reactants are activated by the application of an electric field, including one produced by PVs.
- “Photoelectrochemistry” refers to a technology whereby an electric field is used to promote charge separation of electrons and holes generated through photochemistry. Again, this electric field could also be sourced from PVs.

### 1.3 Outline

The remainder of this review covers the present and future of the MeOH industry. It begins by reviewing the TCST MeOH process using a CO‐rich syngas feed made via conventional syngas production methods. Following this section, the prospect of various solar‐assisted MeOH synthesis processes using feedstocks CO_2_ and water is investigated. The feed gas preparation methods using these raw materials are discussed, including water electrolysis to produce H_2_, the reverse water‐gas shift (RWGS) for producing CO, and solar‐thermochemical methods for producing CO and H_2_ from CO_2_ and water. The principle effects of switching from the commercial feed to one containing greater amounts of CO_2_ are also explored. In later sections, the state‐of‐the‐art technologies for solar MeOH production, including photochemical, photothermal, PV–electrochemical, and photoelectrochemical (PEC), are presented. These technologies are then evaluated in a comparative TEA in Section 7, which is a system‐level analysis investigating the viability of a commercial‐scale solar MeOH process. Following this, the major challenges associated with scaling up such a solar‐assisted process will be addressed. This includes the design and engineering of both catalysts and solar reactors. Next, the prospect of harnessing existing PV technology to meet the energy needs of thermochemical MeOH processes will be discussed. Finally, a brief comparison of the CO_2_ utilization to produce MeOH versus DME will be examined, followed by an assessment of the viability of the most promising TEA solar strategy in an international context.

### 1.4 Definition of Technologies for TEA

The TEA strategies utilize the best available catalysts and technologies for the purposes of comparison. Individual strategies titled as traditional strategy, biomass strategy, and prospective solar strategies A–F are summarized in **Table** **1**. Strategies A and B adopt a syngas production process for MeOH synthesis, but unlike the TCST process, where syngas is produced through steam methane reforming (SMR), in strategy A, H_2_ is produced via water electrolysis and CO is produced via RWGS, while in strategy B, syngas is produced by solar‐thermochemically splitting both CO_2_ and water. In strategies C and D, H_2_ is produced from water electrolysis and MeOH is synthesized from direct hydrogenation of CO_2_, through conventional thermochemical and photochemical technologies, respectively. In strategies E and F, CO_2_ and water are directly converted to MeOH in a single pass, through photochemical and PV–electrochemical technologies, respectively. The six strategies produce solar MeOH from starting feedstocks CO_2_ and water.

**Table 1:** Listing of the technologies used in TEA strategies including the catalyst and feed SN to the MeOH reactor

Strategya)	Section in this review	Technologies	MeOH catalyst	Feed information to MeOH reactor
Traditional commercial strategy	2	TCb) MeOH synthesis, TC syngas from NG	CZA	Syngas 29.00:2.85:68.15 mol% CO:CO_2_:H_2_, SN = 2.05
Traditional biomass strategy	3	TC MeOH synthesis, TC syngas from O_2_ biomass gasifier	CZA	Syngas 29.00:2.85:68.15 mol% CO:CO_2_:H_2_, SN = 2.05
A	4	PV–TC MeOH synthesis, PV–ECc) water splitting, solar‐TC RWGS	CZA	Syngas 29.00:2.85:68.15 mol% CO:CO_2_:H_2_, SN = 2.05
B	4	PV–TC MeOH synthesis, solar‐TC water splitting, solar‐TC CO_2_ splitting	CZA	Syngas 29.00:2.85:68.15 mol% CO:CO_2_:H_2_, SN = 2.05
C	4	PV–TC MeOH synthesis, PV–EC water splitting	CZA	CO_2_ + H_2_ 3.00:22.43:74.57 mol% CO:CO_2_:H_2_ in feed, maximum feed CO_2_ conversion, SN = 2.05 CO produced in situ
D	5	PCd) MeOH synthesis, PV–EC water splitting,	In_2_O_3−_ *_x_*(OH)*_y_*	CO_2_ + H_2_ 3.00:22.43:74.57 mol% CO:CO_2_:H_2_ in feed, SN = 2.05 CO produced in situ
E	5	PC MeOH synthesis	Cu@TiO_2_	CO_2_ + water ≈1:1 CO_2_:water
F	6	PV–EC MeOH synthesis	Mo–Bi	CO_2_ + water ≈1:6 CO_2_:water
Technoeconomic analysis	7	Summary and results for all strategies		

^a)^ All feeds to each strategy are CO_2_ + water, except traditional case NG (natural gas) or biomass + water

^b)^ TC, thermochemical

^c)^ EC, electrochemical

^d)^ PC, photochemical.

Detailed analysis of each technology is presented in Section 7.

## 2 The TCST MeOH Process: Traditional Commercial Strategy

In this section, we review the TCST MeOH process that uses a CO‐rich syngas as feed and the commercial CZA catalyst. First, we will discuss the conventional routes to obtain syngas from NG. Thereafter, the thermodynamics followed by the reaction kinetics of the TCST process are discussed. Finally, a brief discussion of the commercial CZA reaction mechanism will follow.

### 2.1 Conventional Syngas Preparation from NG and Commercial CZA Catalyst Description

The predominant method for current commercial‐scale MeOH production is via thermochemical production from a CO‐rich syngas feed. A typical commercial (empirically optimized) CO‐rich syngas feed composition has a CO:CO_2_ ratio of about 10:1 and a H_2_:CO*_x_* ratio of 2.14. This is on the order of where Klier et al. found their maximum MeOH synthesis rate at 14:1 CO:CO_2_ and H_2_:CO*_x_* of 2.33.^21^ This is also in the region of maximum MeOH production as shown later in Figure 4.

#### 2.1.1 Production of Syngas Feed from NG

The syngas used in MeOH production is a mixture of CO, CO_2_, and H_2_ gases, produced from methane primarily sourced from reforming NG. Its manufacture is a major part of the TCST process and can account for 50–70% of operating costs and 60% of the capital investment.^22^, ^23^, ^24^

Syngas is generally characterized by its stoichiometric number (SN), which is a measure of the reduction potential of the gas mixture. At the commercial scale, an SN of 2.05 has been found to be optimal and maintains the Cu in the CZA catalyst in a redox state.^25^, ^26^ The SN is defined in Equation (1)

(1) $$\mathrm{SN}=\frac{H_{2}-{\mathrm{CO}}_{2}}{\mathrm{CO}+{\mathrm{CO}}_{2}},\;\mathrm{all}\;\mathrm{in}\;\mathrm{mol}\;\mathrm{or}\;\mathrm{mol}\%$$

There are three basic processes for syngas production, shown in **Table** **2**:

- steam methane reforming,
- CO_2_ and methane, or “dry” methane reforming (DMR), and
- partial oxidation of methane (POX).

**Table 2:** Three processes for the production of syngas from methane

Process	Reaction	H_2_/CO ratio	Δ*H*_1173 K_ [kJ mol^−1^]
Steam reforming	CH_4_ + H_2_O ⇌ CO + 3H_2_	3:1	+225.7
Dry reforming	CH_4_ + CO_2_ ⇌ 2CO + 2H_2_	1:1	+258.8
Partial oxidation	CH_4_ + ½O_2_ ⇌ CO + 2H_2_	2:1	−23.1

In general, SMR is the most common process of syngas production. DMR is most interesting from a carbon sequestration standpoint as it consumes both GHGs CO_2_ and methane, and also offers a lower operating cost than SMR and POX. However, since its initial proposal by Fischer and Tropsch in 1928, it has seen limited commercial application as the process suffers from rapid catalyst deactivation through carbon deposition.^23^, ^27^ POX is the most promising from an energy efficiency standpoint;^23^ however, it uses NG exclusively as a carbon feedstock and does not provide the opportunity to valorize any low‐value GHGs like CO_2_.

So‐called “autothermal” reforming or ATR, takes advantage of the varying heats of reaction for these three processes by combining them into one system in which the exothermicity of the POX process complements the endothermicity of the SMR and DMR processes to create a self‐sustaining reaction system. Typically oxygen (O_2_) is fed into the upper stage to combust some of the hydrocarbons, and in the lower stage the generated heat is used in a catalytic bed, driving endothermic reactions cooling the reformed gas as it passes through.

Standard commercial syngas production for a process with capacity of 2500–5000 metric tonnes of MeOH per day (MTPD_MeOH_) usually involves two‐step reforming that consists of an SMR reactor followed by a POX reformer.^22^ Since SMR typically produces more H_2_ than desired for a syngas SN of 2.05, a POX reformer is able to compensate as it typically produces syngas with high CO:CO_2_ ratio, with small amounts of H_2_.

**Figure 3:**
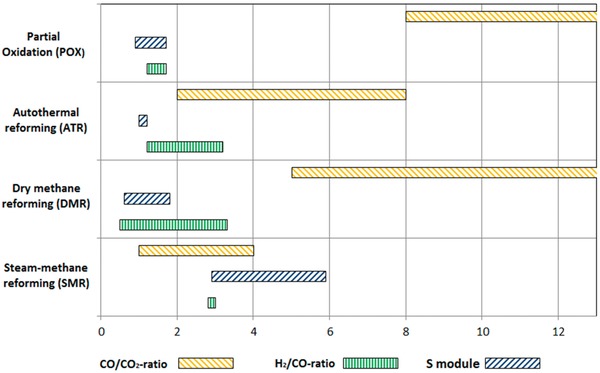
Range of syngas compositions resulting from different reforming technologies. Reproduced with permission.^22^ Copyright 2017, MDPI.

Some key parameters are shown for different syngas technologies in **Figure** **3**. The figure shows each technology compared in terms of CO:CO_2_ ratio, H_2_:CO ratio, and SN (shown as S module) of the product gas. POX and DMR produce high ratios of CO:CO_2_, while ATR and DMR produce syngas with a H_2_:CO ratio near the stoichiometric target of 2:1 H_2_:CO. Of note, however, is that no single technology produces the optimal SN of 2.05.

**Figure 4:**
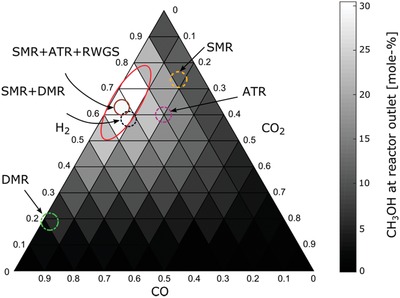
Ternary diagram for optimal CO:CO_2_:H_2_ composition (red ellipse) for the maximum MeOH production at conditions of 5 MPa and 250 °C. The overlayered circles in red, yellow, and green indicate different syngas production technologies that when combined achieve the optimal syngas composition. Adapted with permission.^22^ Copyright 2017, MDPI.

**Figure** **4** shows the optimal syngas composition (red ellipse or lighter area of the ternary diagram) to maximize the MeOH yield (mol% at the reactor outlet), for a reaction taking place at 250 °C and 5 MPa with the CZA catalyst.^22^ As shown, a combination of processes is required, and particular technology adoption is dependent generally on the process economics and local markets.

In terms of process economics, the lowest production cost of MeOH can be achieved by maximizing the process exergetic efficiency (η^exergy^), that is, ensuring that the process is maximizing the available energy to do work based on the energy input. As a trade‐off, higher η^exergy^ often results in higher NG consumption per unit of MeOH produced, also referred to as the “NG intensity.” NG intensity is defined as kg_NG,consumed_ kg_MeOH,produced_^−1^.

An analysis by Blumberg et al. indicated that a combined SMR + ATR syngas system gave the best η^exergy^ of 55.6%, while SMR + DMR and SMR alone produced η^exergy^ of 41% and 28.2%, respectively.^22^ The NG intensities of these three processes follow the same trend of 0.81, 0.78, and 0.53 kg_NG_ kg_MeOH_^−1^ for SMR + ATR, SMR + DMR, and SMR, respectively. Unfortunately, the most efficient processes correlated with NG consumed, making syngas generation less renewable as efficiency increases.

In another study, Luu et al. proposed a combined DMR + H_2_ process, in which H_2_ derived from a renewable source is added to the product of a DMR to achieve the correct SN syngas feed for MeOH synthesis.^9^ They estimated the NG intensity of SMR to be 22% higher than the Blumberg analysis. By scaling the SMR NG intensity to be equivalent to those used in the Blumberg study, after correction, it is shown that the DMR + H_2_ process improves the NG intensity to 0.41 kg_NG_ kg_MeOH_^−1^ (η^exergy^ not given although likely comparable to or higher than SMR + DMR). Furthermore, the DMR + H_2_ process was shown to emit 12.7% less CO_2_ on average than all other reforming systems considered (DMR, SMR + DMR, SMR, ATR). The emission reduction is less than expected, however, because the more CO_2_ in the reformer feed, the less calorific value the gas has, requiring more thermal energy input in the form of NG.

The TCST MeOH process has been extensively optimized for production from NG. Another way to quantify how optimized a process is, besides the η^exergy^, is via an efficiency measuring the so‐called carbon efficiency (η^carbon^). As the name implies, this efficiency is a measure of how much feed NG carbon ultimately results as carbon in the MeOH product. The current highest η^carbon^ uses another type of reforming combination known as gas heating reforming (GHR) and ATR discussed earlier. The GHR uses the hot exhaust gas of the ATR to assist the steam reforming first step lowering its steam requirement and hence the NG intensity.^28^ Please refer to ref. 7 for a recent discussion of NG reforming technologies. The η^carbon^ for this combination is greater than 90%.^7^

#### 2.1.2 Description of the Commercial CZA catalyst

**Figure 5:**
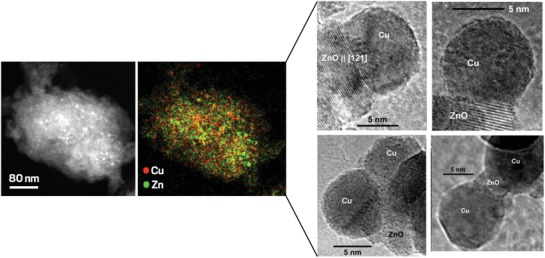
Left: Scanning transmission electron microscopy high‐angle annular dark‐field imaging and energy‐dispersive X‐ray spectroscopy. Cu:Zn atomic ratio is 63:37. Average Cu particle size 7.4 ± 2.1 nm. Reproduced under the terms of the Creative Commons 4.0 license.^36^ Copyright 2016, Springer Nature Publishing AG. Right: High‐resolution transmission electron microscopy images of a Cu/ZnO catalyst obtained from a copper zinc hydroxycarbonate precipitate aged for 120 min. Reproduced with permission.^37^ Copyright 2005, John Wiley and Sons.

There are three main components of the commercial CZA catalyst: Cu metal nanoparticles, ZnO nanoparticles, and the bulk alumina support. It consists of a high Cu‐to‐Zn ratio of approximately spherical Cu nanoparticles of a size around 10 nm, and similarly sized ZnO nanoparticles. They are arranged such that ZnO acts as a Cu spacer forming porous aggregates in the activated state.^10^, ^29^ Effective interaction of Cu with ZnO is thought to prevent Cu sintering, and maintain Cu/ZnO interfacial strain shown to be correlated with catalytic activity.^29^ Furthermore, these two components exhibit what is called a strong metal–support interaction (SMSI) through electronic and morphological changes upon reduction. For the former, electrons are transferred from the partially reduced metal oxide support to the metal via the interface, and for the latter the oxide becomes mobile and with an increased wetting affinity migrates over the Cu particles.^30^ The other supporting material, alumina, is understood to act as a structural promoter.^31^, ^32^ The commercial catalyst aggregates expose a large Cu surface area typically 40 m^2^ g^−1^.^10^ Transmission electron microscopy images of this catalyst are shown in **Figure** **5**.

The atomic Cu:Zn ratio (and molar ratio) is generally greater than 2.5:1, with a Süd‐Chemie patented example between 2.8:1 and 3.8:1, as defined in US Patent 4535071.^33^ A typical unreduced commercial MK‐121 CZA catalyst composition from Haldor Topsøe contains a minimum of 55 wt% CuO, 21–25 wt% ZnO, and 8–10 wt% Al_2_O_3_ (Cu:Zn molar ratio of 2.3–2.7).^10^ A CZA catalyst from the chemical supplier Alfa Aesar contains 63.5 wt% CuO, 25 wt% ZnO, 10 wt% Al_2_O_3_, and 1.5 wt% MgO (Cu:Zn molar ratio of 2.6).^34^ The ICI catalyst, originally developed in the mid‐1960s, is expected to be of similar composition, and as of 2009 contributes to 60% of TCST MeOH production, while other major processes like Lurgi, MGC, and Kellogg make up the bulk of the remainder 27%, 8%, and 3%, respectively.^1^ The pellet sizes are roughly 5 mm × 5 mm and bulk densities of unreduced catalyst are 1200–1300 kg m^−3^.^10^

Commercial CZA catalysts have a productive lifetime of roughly 4–5 years at commercial operating conditions.^35^ Catalyst deactivation becomes important when a significant amount of water is present in the system typically from higher CO_2_ in the feed, a topic that will be discussed in Section 4.3.2.

An overview of commercial catalyst compositions, as well as conversion rates and space–time yields (STYs), are shown in **Table** **3**.^10^ The last row of the table shows how our model using fresh catalyst (the results of which will be discussed later) compares to other STYs using commercial CZA catalyst systems.

**Table 3:** Commercial CZA catalyst compositions and activities (adapted from ref. 10)

Process	Catalyst composition [wt%]	Conditions: Temperature [°C] Pressure [MPa] Space velocity [h^−1^]	Rate [kg_MeOH_ kg_cat_^−1^ h^−1^]	STY [kg_MeOH_ L_cat_^−1^ h^−1^]	Yield MeOH [mol%]
Shell International Research^38^	Cu–Zn–Ma) 40:18:4	300 5.3 10 900	–	1.01	–
Ammonia Casale^39^	Cu–Zn–Cr–Al 30:50:16:3	250 10 12 500	–	1.00	–
Süd‐Chemie AG^40^	Cu–Zn–Al 65.2:23.8:11	300 10 4000	–	0.82	–
Süd‐Chemie AG^41^	Cu–Zn–Al 63:27:10	250 6 22 000	1.144	1.19	–
Lonza AG^42^	Cu–Zn–Zr 40:20:40	250 5 8000 L kg^−1^ h^−1^	0.54	–	12.7
AIST, RITE^43^	Cu–Zn–Al–Zr–Si 45.2:27.1:4.5:22.6:0.6	250 5 10 000	–	0.72	–
YYK Corp^44^	Cu–Zn–Al 76.3:11:12.7	250 5 1.7 g h^−1^ mol^−1^	1.548	–	–
Kinetics model (this review)^45^	ICI 51‐2 Cu–ZnO–Al_2_O_3_ (fresh catalyst)	250 8 9900	2.14	2.83	33.5

^a)^ M is a metal.

### 2.2 Thermodynamics of the TCST MeOH Process

In the TCST process, there exist a number of chemical reactions occurring in parallel. While input syngas contains a large portion of CO, MeOH is produced primarily from CO_2_ in an exothermic reaction (Equation (2))

(2) $${\mathrm{CO}}_{2}+{\mathrm{3H}}_{2}⇌{\mathrm{CH}}_{3}\mathrm{OH}+H_{2}\mathrm{O,}\;\Delta H_{\mathrm{298}\;K}=-\mathrm{49}.6\;\mathrm{kJ}\;{\mathrm{mol}}^{-1}$$

The water‐gas shift (WGS) reaction also occurs as a side reaction (Equation (3))

(3) $$\mathrm{CO}+H_{2}O⇌{\mathrm{CO}}_{2}+H_{2},\;\Delta H_{\mathrm{298}\;K}=-41.3\;\mathrm{kJ}\;{\mathrm{mol}}^{-1}$$

The presence of these two reactions results in the overall reaction equation from syngas (Equation (4))

(4) $$\mathrm{CO}+2H_{2}⇌{\mathrm{CH}}_{3}\mathrm{OH},\;\Delta H_{\mathrm{298}\;K}=-90.9\;\mathrm{kJ}\;{\mathrm{mol}}^{-1}$$

The WGS reaction occurring in parallel sets up a feedback loop wherein water is consumed, producing additional reactants for the MeOH synthesis reactions (Equations (2) and (4)), driving the equilibrium to favor higher MeOH yields.

**Figure 6:**
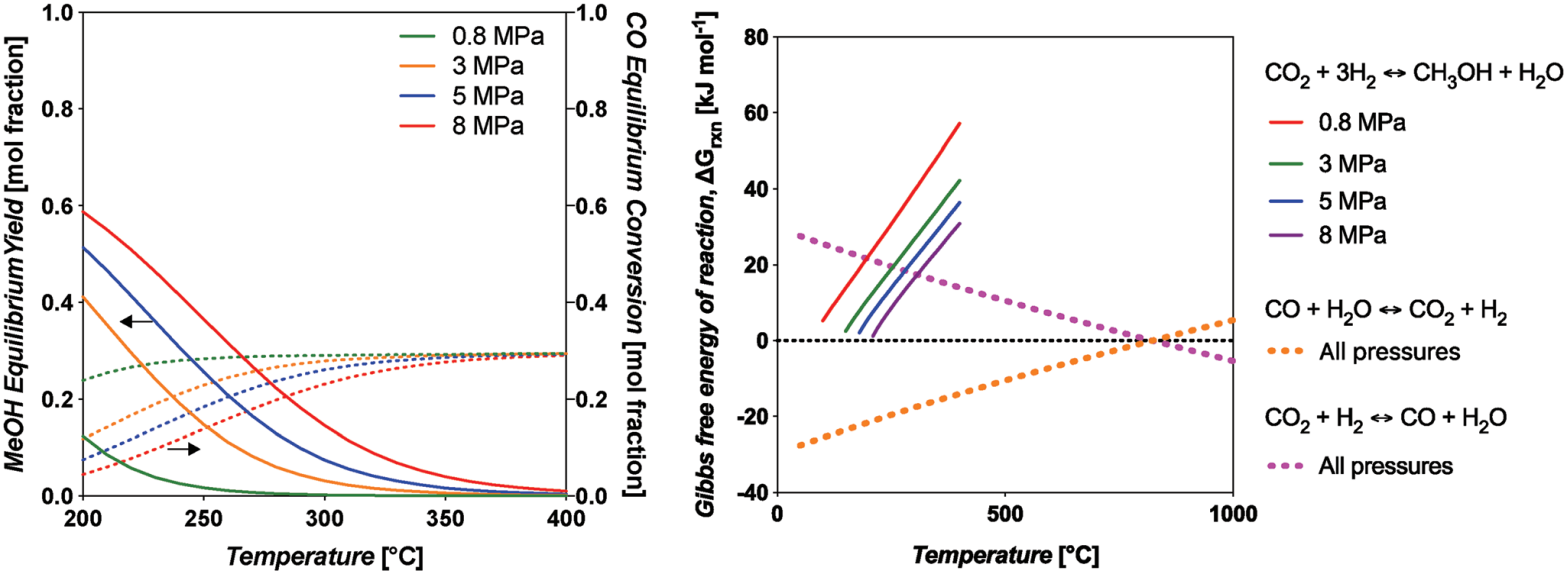
Left: Equilibrium yields of MeOH and CO in the standard CO‐rich TCST MeOH synthesis process. Feed syngas utilized was 29:3:68 mol% CO:CO_2_:H_2_. CO is consumed by the WGS side reaction. This plot shows that MeOH yield increases with decreasing temperature and increasing pressure. The WGS reaction is more prevalent at lower temperatures and higher pressures. Right: Free energy diagram at various pressures for the main MeOH synthesis reaction and side reactions WGS and RWGS for CO‐rich syngas feed.

Thermodynamic equilibrium analysis was conducted using Aspen Plus V9 software, using a Gibbs reactor block with the Peng–Robinson equation of state. The Gibbs reactor considers all possible reactions capable of producing the inputted products (H_2_, water, CO, CO_2_, MeOH) from reactants (CO, CO_2_, H_2_). The Peng–Robinson property package is applicable to equilibrium systems containing MeOH.^46^ The equilibrium yield of MeOH is shown in **Figure** **6**, left, and indicates that it increases with decreasing temperature and increasing pressure. At 200 °C and 8 MPa constant pressure, the yield is 58.8 mol%. When the temperature is increased to 250 °C, the yield decreases to 36.6 mol%.

Further, the thermodynamic analysis shows that the equilibrium conversion of CO increases with increasing pressure and decreasing temperature, and by being consumed it improves the equilibrium conversion of MeOH. The Gibbs free energy diagram for Equations (2)–(4) is shown in Figure 6, right. It shows that the WGS is favorable at all temperatures up to ≈1000 °C, but becomes less so as *T* increases; alternatively, the RWGS becomes more favorable as temperature increases. For the main MeOH synthesis reaction (Equation (2)), the Gibbs free energies equate to fractional equilibrium constants, *K*'s, at temperatures above 100 °C.

### 2.3 Reaction Kinetics of the TCST MeOH Process

The TCST MeOH process using the CZA catalyst has been optimized from over 60 years of process experience. Adding a small quantity of CO_2_ to the syngas achieves the optimal SN and has been empirically determined to augment the MeOH synthesis reaction (primarily Equation (2)). The reason for this augmentation has been determined experimentally, by noting that as the small quantity of CO_2_ produces water via MeOH synthesis and RWGS reactions, the water then acts to have an autocatalytic effect on these same reactions and possibly the WGS as well.^10^, ^47^ By adding a small amount of CO_2_ into the syngas feed, the water can be produced in situ.

To illustrate the rates and selectivity of the commercial CZA catalyst using a CO‐rich feed, reaction kinetics modeling was conducted using E‐Z Solve by Intellipro, Inc., a numerical ODE integration software. The model is 1D plug flow, isothermal (250 °C), in the reaction regime (no transport limitations), isobaric (0.8, 3, 5, 8 MPa), with pressure drop neglected and 9900 h^−1^ contact velocity. The integration method used for the model is backward differentiation formula order 1–5, step size 0.01 g of CZA catalyst up to 1 g basis. This reaction kinetics model adapted from ref. 45 is valid over the experimental range of Klier et al.^21^ for *P*_CO_:*P*_CO2_ ratios of 0.5–10. This model was validated against %CO*_x_* (the sum of CO + CO_2_ mol%) conversion data from ref. 21 at their maximum experimental conversion at 250 °C, 7.5 MPa, and 28:2:70 mol% CO:CO_2_:H_2_.

For synthesis from a CO‐rich syngas, the syngas feed used in the model had a composition of 29.00:2.85:68.15 mol% CO:CO_2_:H_2_ (SN = 2.05). This composition also satisfies the additional commercial practice of maintaining a high CO:CO_2_ ratio to increase the reaction rate and %CO*_x_* conversion to MeOH.^48^

**Figure 7:**
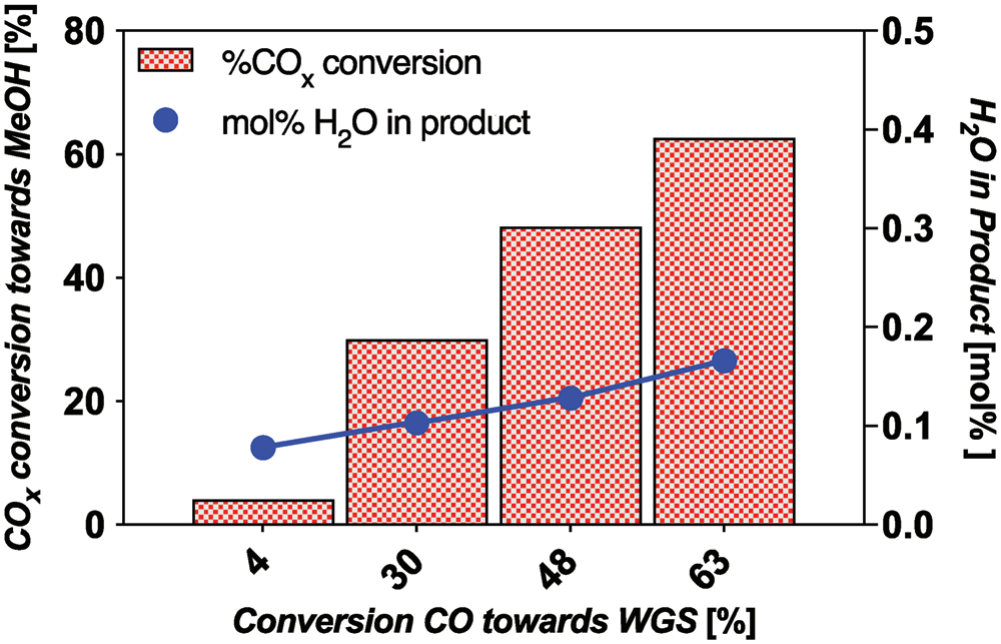
The percent conversion of carbon sources in the system (CO + CO_2_ or feed %CO*_x_*) that goes toward both the main MeOH synthesis reactions, and those that go toward the WGS side reaction for the TCST MeOH process from a CO‐rich syngas feed. Each bar represents a pressure starting at 0.8 MPa to 3, 5, and 8 all at 250 °C and SV 9900 h^−1^. The mol% H_2_O resulting in the product stream is also shown.

The results for the %CO*_x_* conversion to MeOH (Equations (2) and (4)) and %CO conversion to CO_2_ (Equation (3)) are shown in **Figure** **7**. The figure shows there is a synergistic effect of water consumption by the WGS reaction on promoting the total carbon conversion toward MeOH.

Percent feed %CO*_x_* conversion becomes as high as 63% at commercial conditions of 8 MPa, 250 °C, CO‐rich feed, and space velocity (SV) of 9900 h^−1^ (space velocity is discussed further below). This indicates that with significant CO in the feed, at high pressure, the system consumes excess water, driving conversion to ≈92% of the theoretical equilibrium conversion, as per the Le Châtelier principle.

The SV, mentioned earlier, is a parameter that indicates the reactant gas–catalyst contact time in a flow reactor system. It is defined as the flow of feed gas, *q* (m^3^ h^−1^) at standard conditions (STP, 0 °C and 0.1 MPa) per bed volume of catalyst, *V*_cat_ (m^3^), calculated using the apparent density of the material. This results in units of inverse time (h^−1^). In the model, a typical SV of near 10 000 h^−1^ (9900 h^−1^) is used, calculated with an apparent catalyst density of 1320 kg m^−3^ for as‐purchased CZA (Alfa Aesar).

Efficient operation of the TCST process is a balance of a number of parameters. They are yield, water production or consumption, cooling requirements, and throughput. The typical yield of the TCST process is 14–20 mol% MeOH (partially deactivated CZA) usually achieved with a SV near 10 000 h^−1^. With the high CO content in the commercial feed, the water content in the product gas is limited to ≈0.2 mol% (see Figure 7). Longer contact times generally result in higher %CO*_x_* conversion and therefore water in the catalyst bed. This higher conversion would be at the expense of catalyst lifetime, due to water‐induced deactivation (to be discussed) as the CO concentration decreases along the bed. Furthermore, a lower yield is chosen in order to maintain manageable cooling requirements due to the exothermicity of both the MeOH (Equation (2)) and WGS (Equation (3)) reactions. Longer contact times also have the effect of reducing reactor throughput, and necessitate the construction of additional reactors to compensate.

#### 2.3.1 Fundamental Reaction Mechanism for MeOH Synthesis on a Commercial CZA Catalyst

To begin, it has been observed experimentally that the carbon atom incorporated into the MeOH molecule can originate from either CO or CO_2_ depending on the reaction temperature.^47^ At lower temperatures (below 240 °C), the carbon contained in the MeOH was demonstrated to originate predominantly from CO rather than CO_2_ via ^13^C labeling. As the temperature is increased above 240 °C, in the same experiment it was similarly shown that the CO_2_ carbon becomes the dominant reactant.^47^ At the commercially relevant temperature of 250 °C, 80% of the carbon in the MeOH produced is from CO_2_ at 0.6 MPa pressure.^47^

There has been debate in the field over the predominant reaction pathways responsible for MeOH synthesis. A key point of contention is the identity of the main intermediate species. One pathway involves the formation of the formate intermediary at the catalyst surface, whereas the alternative proposal claims a carboxyl intermediate. Both of these pathways will be discussed herein.

**Figure 8:**
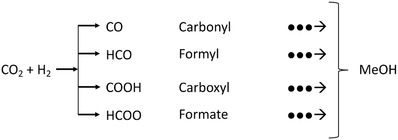
Possible reaction pathways that a CO_2_ species can take for conversion to MeOH. Identification of the predominant mechanism remains of considerable debate. Adapted with permission.^49^ Copyright 2018, Wiley‐VCH.

The main challenge has been to identify, under realistic temperatures and pressures, which of the possible surface intermediates (carbonyl (CO), formyl (HCO), carboxyl (COOH), or formate (HCOO)) participates in the dominant reaction pathway. This is shown in **Figure** **8**.

An additional challenge has been to comprehend the synergistic role of the Cu and metal oxide support in the catalyst, as a result of metal–support interactions. In this regard, a particularly elegant and deeply analytical recent study by Larmier et al. of MeOH synthesis uses ZrO_2_ supported Cu nanoparticles of around 2 nm in size.^50^

This study used a combination of in situ Fourier‐transform infrared spectroscopy (FTIR) and ex situ magic‐angle spinning NMR together with ^2^D and ^13^C isotope labeling to show that hydrogenation of the surface reaction intermediate, formate, was the dominant reaction pathway. Their experimental findings were corroborated through the use of density functional theory (DFT) computational modeling.

DFT calculations on this system have concluded that a metal–support interaction occurs via charge transfer from the Cu nanoparticles to CO_2_ adsorbed on the ZrO_2_ (opposite charge direction to the partially reduced metal oxide SMSI mentioned earlier). This accumulation of negative charge on the C of surface‐bound CO_2_ activates the CO_2_ on the surface of the ZrO_2_ toward reaction with H species that have spilled over onto the ZrO_2_ support from dissociation of H_2_ on the Cu surface. This reaction results in a surface‐bound formate species, thought to be located at the interface between the Cu and ZrO_2_.^50^

**Figure 9:**
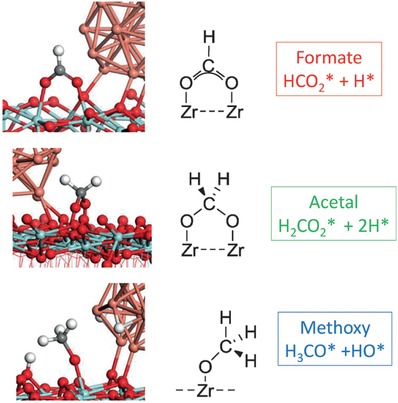
Proposed reaction mechanism determined via DFT modeling for MeOH synthesis from syngas, on a Cu/ZrO_2_ catalyst. Reproduced with permission.^50^ Copyright 2017, John Wiley and Sons.

The favored reaction scheme is depicted in **Figure** **9**, for the transformation of this surface‐bound formate intermediate to a surface methoxy, determined from combined IR–NMR spectroscopic measurements. Through use of kinetic isotope labeling, an interpretation of the isotope labeling results indicates that the observed H–D exchange of the formate C—H bond to form DCOO is faster than its hydrogenation to surface methoxide (OCHD_2_), verifying the DFT prediction.

To complement, the DFT calculations showed that there exists a small activation energy barrier for interconversion between formate and acetal species, implying they exist in equilibrium, thereby enabling the observed H–D exchange of D_2_ with the HCOO intermediate. The reaction of the acetal that follows involves a concerted transfer of H from the Cu to the acetal C, which results in the formation of surface methoxy and hydroxyl groups. They can react further with H spilled over from the Cu to form MeOH and water that desorb from the ZrO_2_ to complete the catalytic cycle, with desorption energies determined through DFT modeling.

Conversely, there are recent studies that suggest an alternative to the formate pathway. Yang et al.^47^ attempted to titrate surface formate species on Cu and on supported Cu/SiO_2_ present under reaction conditions (0.6 MPa, <200 °C) with H_2_, but failed to produce MeOH, possibly ruling out a hydrogenation pathway via formate in the synthesis mechanism. However, they remarked that this pathway may be more favorable at higher surface coverages of formate (e.g., through use of higher *P*).

The times to initiate the reaction, or the induction times, of both MeOH synthesis and RWGS reactions were found in this study to be influenced by the amount of water in the feed.^47^ However, the presence of water did not affect the formate coverage, indicating that this species is likely a spectator decoupled from the two main reaction paths, a conclusion of ref. 51 also for an industrially relevant CZA catalyst. This suggests that water did not have an autocatalytic effect on a hypothetical active formate intermediate.

Based on the Yang et al.'s study, water is thought to contribute to the creation of active intermediates that may function catalytically with regard to the hydrogenation steps in both CO and CO_2_.^47^ The above assumption suggests a common intermediate in both the MeOH synthesis and RWGS reactions. A carboxyl intermediate instead of the usual formate (for CO_2_) and formyl (for CO) is thought to answer the question related to the influence of water on the induction time.

**Figure 10:**
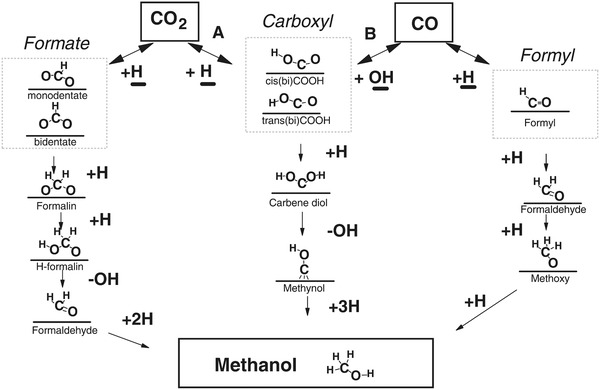
Postulated reaction mechanisms for MeOH synthesis from a CO and CO_2_ syngas on a Cu/SiO_2_ catalyst. Reproduced with permission.^47^ Copyright 2013, Elsevier.

They continued investigating using DFT studies, showing that the presence of water was found to greatly assist the hydrogenation of CO_2_ to form carboxyl species. The hydrogenation barrier was 1.08 eV for the carboxyl pathway versus 1.8 eV for the formate pathway with water. The effect of water was hypothesized to assist the CO hydrogenation, as the OH groups from the water are necessary to form the carboxyl intermediate. Corroborating DFT data failed to find a water‐assisted step for the reaction pathway involving a formate intermediate, further supporting the evidence of a water‐assisted shared carboxyl intermediate species in the reaction pathway. Although the carboxyl intermediate species had not yet been observed experimentally, there is growing evidence for this water‐assisted intermediate that is shared between the CO and CO_2_ reaction pathways. The mechanistic details are illustrated in **Figure** **10**.

Recently, a study by Chernyshova et al. using surface‐enhanced Raman spectroscopy identified that CO_2_ activates as a carboxyl intermediate on a 0.5 mm thick Cu foil surface.^52^ This was confirmed by DFT simulations. This evidence of a carboxyl species lends credence to Yang et al.'s proposed mechanism of a dual pathway consisting of both formate and carboxyl intermediates. This result also implies a direction of electron charge transfer via SMSI from a partially reduced metal oxide to activating CO_2_ on Cu particles.^30^ H_2_ for hydrogenation of CO_2_ can originate from spillover of H^+^ from ZnO,^53^, ^54^ as it was found through experimental observation that absorbed H coverage on metallic Cu hinders more dissociative H_2_ adsorption, but not CO_2_.^55^ Oxidized Cu has been found to provide more H spillover to ZnO than metallic Cu (where C oxygenates are proposed to reside) lending empirical understanding of the redox SN of the syngas.^54^

## 3 Thermochemical MeOH Production from Biomass: Traditional Biomass Strategy

The modern production of syngas from gasified biomass remains the sole means of commercial‐scale MeOH production that incorporates both sunlight and biological processes, with overall biomass‐to‐MeOH mass conversions reaching as high as 45%.^56^ Though respectable, this represents only about one‐fourth of the mass conversion expected when producing MeOH from NG (based on stoichiometric calculation and assuming a methane‐to‐MeOH η^carbon^ of 90%). The term “biomass” refers to any carbonaceous plant or animal material, including that derived from wood, agricultural crops, municipal and animal waste, aquatic plants, and algae.^1^ While conversion of biomass to fuel is theoretically carbon‐neutral, it is not highly efficient in terms of the amount of initially captured solar energy that remains available in the final chemical product. Losses occur at several steps of the process, including those due to low photosynthetic efficiency (1–2% for plants^57^ and up to 3% for microalgae,^58^ as opposed to up to 13% for hybrid systems consisting of microorganisms coupled to PVs^59^), biomass processing, and fermentation steps. Depending on the source of material, large‐scale production of biomass may also compete with the availability of arable land for food production.

### 3.1 Syngas Production from Biomass

Using a thermochemical process called “gasification,” biomass can be converted into syngas, which is in turn used in catalytic MeOH synthesis. As a result, the combination of these two processes can be considered an applicable use of solar energy in MeOH production.

The gasification of biomass may be accomplished via single‐step or two‐step processes. In both cases, the biomass is first dried to reduce its moisture content to between 15 and 20 wt%.^1^ In a one‐step process, the dry biomass is pressurized in an O_2_/water mixture and then partially combusted to provide the heating required to drive the reaction.^1^ While such a simple process is very appealing, a more involved, two‐step process typically achieves more complete conversion to products.^1^ In the first of the two steps, the biomass is pyrolyzed at between 400 and 600 °C in an O_2_‐deficient atmosphere to form both charcoal (weighing 10–25% of the initial mass) and a gaseous mixture of small molecules that includes H_2_, methane, water, CO, CO_2_, and volatile tars.^1^ The charcoal product is then gasified at 1300–1500 °C in a process called “char conversion,” effecting its conversion into CO.^1^ For both of these processes, the major disadvantage and challenge facing the gasification of biomass is the proper recovery of heavier tar compounds that can condense and clog various components of the chemical reactor; proper planning and control of reaction conditions are required in order to avoid production of these compounds.^1^

While all biomass is capable of being gasified, not all sources achieve the same biomass production rates or final conversion to MeOH.^1^ Although industrial facilities have been proposed for the production of biomass using algae,^60^ their high water content makes them less appealing for gasification.^1^ These organisms also tend to be rich in oils that are better suited for conversion into biodiesel,^61^ and so less effort has been directed toward their use in MeOH production.^1^ As is reflected by their prevalence in the literature, dry wood and stalk plants (switchgrass, rice, corn straw, etc.) are typically better suited to gasification and subsequent incorporation into the process.^56^, ^62^, ^63^ Crops such as switchgrass are also an attractive option for generating biomass in countries with arable land, but run the risk of competing with food crops for land use.^1^ As such, the farming of fast‐growing trees is a promising approach to biomass generation for subsequent gasification; however, there is considerable variability in the growth of different sources of biomass, with each MT_dry wood_ biomass requiring 0.025–0.1 hectares of land (250–1000 m^2^).^1^ To feed a hypothetical 24 MTPD_MeOH_ reactor for an entire year would require ≈48 MT_biomass_ to be processed, necessitating the farming of ≈220–880 hectares of land.^1^ On a commercial scale, syngas production from biomass is impaired by its relatively low energy density (in terms of the products it generates), making its harvesting and transport over long distances less economical.^1^ The development of novel technologies such as “fast pyrolysis,” which can convert biomass into a higher‐density “biocrude” oil prior to transport, could help offset this disadvantage, but will require significant further research in order to transition from the laboratory to the commercial scale.^64^ The bioliq process spearheaded by the Karlsruhe Institute of Technology in partnership with company Air Liquide was fully commissioned in 2013. The process concentrates dry biomass by converting it into a “biosyncrude,” increasing its energy density by an order of magnitude (≈2 to ≈25 GJ m^−3^). The biosyncrude is collected and transported to a centralized facility that then converts it into diesel and gasoline‐type fuels (≈36 GJ m^−3^). This is another route to liquid chemicals being pursued in Germany.^65^, ^66^

The syngas produced from biomass is similar to that obtained from NG in that both typically have low concentrations of impurities (such as sulfur and heavy metals) relative to syngas derived from coal.^1^ As mentioned previously, an SN slightly greater than 2 is optimal for the production of MeOH from syngas, which is greater than the ratio of that obtained from biomass.^56^ The value of SN can be increased through either the addition of H_2_ (which can also be derived from biomass via fermentation and other reactions)^67^, ^68^, ^69^ or the utilization of the WGS to convert some of the excess water to H_2_.^56^

Methods of using solar energy for the gasification of biomass have also been proposed, further contributing to the coupling of solar energy with MeOH production.^70^ In this process, biomass is heated either directly, through the use of a transparent (e.g., quartz) reactor, or indirectly, by heat transfer through the exterior walls of an opaque reactor.^70^ Each approach offers advantages and disadvantages: a transparent reactor must be diligently cleaned to enable the transmission of light, whereas an opaque reactor must contend with limitations of heat transfer from the exterior of the reactor to the biomass inside.^70^ Using solar concentrators in conjunction with complementary focusing elements intensifies the sunlight incident on the biomass gasification reactor. The temperatures thereby achieved should be sufficient to effect biomass gasification (i.e., up to 850 °C) without the need for external heating.^70^ It is proposed that 3 MW of solar energy would need to be harnessed by the solar concentrators in order to process 24 MTPD_biomass_ of dry (i.e., 15–20 wt% moisture) pulverized biomass.^70^ Assuming a near‐equatorial location and full utilization of the available solar spectrum,^71^ this would require (at first approximation) a minimum area of ≈1.7 hectares, corresponding to a 146 m diameter circular array of solar concentrators.

In recent years, systems have also been designed in which biomass is further converted from syngas to MeOH, within the same facility.^60^, ^62^, ^72^, ^73^ The theoretical maximum energy efficiency of photosynthesis (light to biomass) is estimated to be 4.6–6.0%.^57^ Here a 5% photosynthetic efficiency is assumed. It should be noted, however, that syngas produced via the gasification of biomass is significantly enriched in CO_2_, relative to the optimal CO:CO_2_:H_2_ compositions used in the TCST process. This discrepancy can be quite large, with CO:CO_2_:H_2_ compositions of ≈20:30:50 mol% (SN of 0.4) being produced from a variety of waste biomass from various grasses, trees, and crops.^72^

Thus, syngas produced from biomass contains excess CO_2_ and insufficient H_2_. Fortunately, the relative ratios of the three constituent gases can be modified to more closely match those of the ideal feed case via various processes; these include the removal of excess CO_2_ using membranes or amine scrubbing, conversion of excess CO_2_ to CO using the RWGS reaction, and addition of H_2_ produced by other processes.^60^, ^62^, ^72^ In order to achieve maximum yields, such an approach was used in the “traditional biomass strategy” discussed in this section, wherein removal of excess CO_2_ was used to obtain an optimal syngas composition for MeOH production.^72^ These extra steps do contribute to the energetic and capital costs of the overall synthesis process, however, and would ideally be avoided, thereby allowing CO_2_‐rich syngas (such as that produced via biomass gasification) to be used directly and without further modification. For this reason, there exists considerable interest in incorporating such syngas into more traditional MeOH production systems.

## 4 Thermochemical MeOH Production Using a CO_2_‐Rich Feed: Strategies A–C

Incorporation of more CO_2_ into the thermochemical process and other useful chemical processes using novel catalysts or process designs is an aspirational goal, to close the carbon cycle in which fuels are recycled from the atmosphere instead of derived from nonrenewable fossil resources. However, replacing CO in the thermochemical process syngas feed with CO_2_ has implications for the TCST process. In this section, we review the less conventional routes to obtaining syngas from CO_2_ and water, which feeds a thermochemical MeOH synthesis process using the commercial CZA catalyst. First, we will introduce light‐assisted syngas production technologies (RWGS, water electrolysis, photoelectrochemical water electrolysis, and solar‐thermal), and discuss the fundamental basis and materials underlying the processes (photothermal, electrochemical, photochemical, and solar‐thermochemical). We will then discuss the thermodynamic and reaction kinetic consequences of increasing the CO_2_ feed concentration on the thermochemical process. Thereafter, the effects of increased water production (due to CO_2_‐rich feed) and the advantages of tuning selectivity for a solar‐assisted reaction will be discussed. Finally, a description of a novel type of low‐temperature thermochemical catalyst will be discussed.

### 4.1 Solar‐Assisted Syngas Production: From CO_2_ and Water to CO and H_2_

#### 4.1.1 Photothermal CO Production via the RWGS Reaction: Utilized in Strategy A

Syngas is the main feedstock for current MeOH production, and CO represents a major component thereof. Due to the high cost of building and operating the syngas producing process in the TCST process, it is worthwhile exploring various technologies that could supplant this step for less overall capital expenditure and operating costs. The RWGS reaction is a key candidate for making solar syngas to valorize CO_2_.

The RWGS reaction was first observed by Carl Bosch and Wilhelm Wild in 1914, when they attempted to produce H_2_ from steam and CO on an iron oxide catalyst.^74^ It is currently utilized at the pilot plant scale in the production of syngas for producing “blue crude,” a CO_2_‐derived crude oil similar to fossil crude oil in carmaker Audi's e‐diesel process.^75^

**Figure 11:**
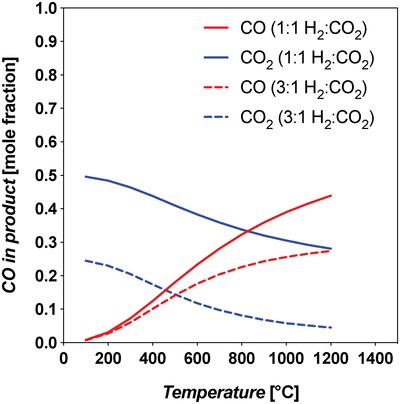
RWGS equilibrium product syngas composition on a dry basis (Aspen Plus V9) at two different feed compositions and varying temperature

The RWGS reaction is equilibrium limited and favored at high temperatures, due to the endothermic nature of the reaction. Thermodynamic evaluations at atmospheric pressure showed that CO_2_ conversion is enhanced when excess H_2_ is added (92% versus 62% CO_2_ conversion for 3:1 H_2_:CO_2_ versus 1:1 at 1200 °C) and the reactor temperature is high.^76^ The equilibrium concentrations for 1:1 H_2_:CO_2_ and 3:1 H_2_:CO_2_ on a dry basis (product water removed) are shown in **Figure** **11**.

It is possible to drive traditional thermochemical reactions with solar energy through the use of photothermal catalysts. In photothermally catalyzed reactions, incident photons are absorbed by the photocatalyst and converted to heat. This results in a temperature spike localized to the catalytic sites on the surface of the catalyst. The generation of high local temperatures at the surface of the catalyst can result from localized surface plasmons, recombining electron/hole pairs, and relaxing interband and/or intraband transitions. Nanoscale thermal transport also plays an important role in either localizing or distributing the generated heat to catalytic sites and/or nanoscale thermal transport effects.

The extent of the photothermal effect depends on the structure and composition of the photothermal catalysts. A composite material hybrid configuration offers several advantages by drawing on the electronic, optical, catalytic, structural, and chemical properties of two materials, rather than one. Generally, a strongly absorbing material is coupled with a (photo)catalyst. Both of these materials can act as either the catalyst or the catalyst support. An ideal photothermal catalyst should be a strong light absorber across the whole spectral range from ultraviolet to infrared. At the same time, it should efficiently convert this radiation into thermal energy and catalyze the desired surface reaction at high rate and selectivity for the desired product.

When a photothermal catalyst absorbs light, a local temperature spike is observed. Hence, the temperature experienced at the active site increases, which leads to increased reaction rates. At the same time, photochemical surface reactions are initiated as a result of illumination of the catalyst.

The synergistic effects of photo‐ and thermochemical processes experienced during photothermal catalysis can lead to increased rates of reaction, thereby contributing to enabling higher CO_2_ conversion, and lower demand for high‐grade, high‐temperature heat.

**Figure 12:**
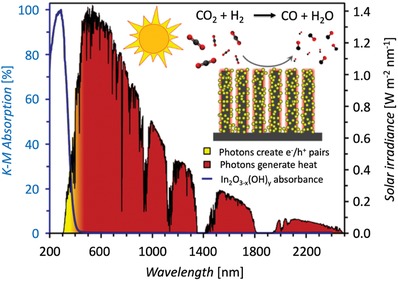
Schematic representation of photothermal catalysis using In_2_O_3−_ *_x_*(OH)*_y_* photocatalyst supported on Si nanowires. The yellow shading represents the photons of energy greater than the bandgap of In_2_O_3−_ *_x_*(OH)*_y_* photocatalyst. The red shading represents the photons of energy below the bandgap of In_2_O_3−_ *_x_*(OH)*_y_*. Photons of energy above the bandgap of the In_2_O_3−_ *_x_*(OH)*_y_* are absorbed and generate electron/hole pairs, while the photons of energy below the bandgap of In_2_O_3−_ *_x_*(OH)*_y_* are absorbed by the Si nanowire support to generate heat. Reproduced with permission.^85^ Copyright 2016, American Chemical Society.

Photothermal RWGS catalysts oftentimes consist of metal or metal oxide catalysts, which exhibit strong optical absorption across the full spectral range, and semiconductor supports. The metal/metal oxide catalysts have a dual purpose: they act as efficient light absorbers, and at the same time catalyze the reaction. The semiconductor support can also act as light absorber, or it can provide active surface sites for the adsorption of reactants and reaction intermediates. Metals such as Fe,^77^, ^78^ Co,^78^ Pd,^78^, ^79^, ^80^, ^81^ Ni,^78^, ^82^ and Rh^83^ have been reported as efficient photothermal catalysts for the RWGS reaction. Supports, which have been studied, include Al_2_O_3_,^78^ Nb_2_O_5_,^80^, ^84^ Si nanowires,^85^ TiO_2_,^78^ and WO_3_.^79^ Photothermal RWGS over the aforementioned photochemical catalyst In_2_O_3−_ *_x_*(OH)*_y_* deposited on Si nanowires will be discussed in more detail. To expand, In_2_O_3−_ *_x_*(OH)*_y_* has been discovered recently to efficiently photocatalyze the RWGS reaction.^86^, ^87^, ^88^, ^89^ The optical bandgap of In_2_O_3−_ *_x_*(OH)*_y_* is 2.9 eV.^86^ It has been shown through a combination of experimental and theoretical work that the absorption of ultraviolet and visible radiation with an energy above the optical bandgap of In_2_O_3−_ *_x_*(OH)*_y_* elevates the photocatalyst into an excited state. This causes a reduction in kinetic barriers for the RWGS reaction, which manifests itself in higher observed catalytic rates.^87^, ^90^ Appreciable rates in the range of µmol CO g_cat_^−1^ h^−1^ have been observed only when the photocatalyst temperature is elevated to above 100 °C. Experimentally, temperature elevation is achieved through bringing the reactor to the desired temperature through auxiliary heating. The desired working temperature can also be achieved through dispersing the In_2_O_3−_ *_x_*(OH)*_y_* catalyst on Si nanowires. The Si nanowires efficiently absorb broadband light across the whole spectral range, which causes temperature elevation. Hence, the temperature needed to achieve appreciable rates can be achieved through light‐induced heating of the Si nanowire support, as illustrated in **Figure** **12**.^85^ This photothermal effect increases the efficiency of the light‐induced RWGS catalytic reaction.

The yellow and red shading in the figure illustrates the different light harvesting processes for photons with energy greater and less than the bandgap of In_2_O_3−_ *_x_*(OH)*_y_*, respectively. The yellow shading is the creation of an electron–hole pair within the nanocrystals and enables the solar‐powered RWGS reaction, whereas the red‐shaded components are absorbed and, via the photothermal effect, converted into heat within the SiNW support. These studies showed that the hybrid In_2_O_3−_ *_x_*(OH)*_y_*@SiNW made more efficient use of the solar spectrum than In_2_O_3−_ *_x_*(OH)*_y_* alone. Collectively these effects enhanced the photocatalytic reduction rate of CO_2_ relative to nonuniform coatings and unsupported In_2_O_3−_ *_x_*(OH)*_y_*.

The champion photothermal RWGS rate of 18.8 mol CO g_cat_^−1^ h^−1^ has been achieved using Pd‐decorated Nb_2_O_5_ catalysts.^84^ In this study, a library of Pd@Nb_2_O_5_ catalysts of various Pd loadings was prepared. The selectivity between the CO RWGS reaction and the CH_4_ Sabatier reaction can be tuned by varying the Pd loading between 0.1 and 15 wt% Pd. At low Pd loading of 0.1 wt%, the RWGS is catalyzed at a rate as high as 18.8 mol CO g_cat_^−1^ h^−1^ and at selectivity of greater than 99%. If the loading is increased, the selectivity shifts toward CH_4_ formation. At a Pd loading of 10 wt%, CH_4_ is formed at a rate of 0.11 mol g_cat_^−1^ h^−1^ and at a selectivity of 13%. This effect has been rationalized through a combination of electronic and size effects. As the loading increases, the average Pd nanoparticle size increases. This effects stronger electronic interaction between the Pd nanocrystals and the Nb_2_O_5_ support, which causes the Pd nanocrystal surface to be more positively charged than for smaller Pd nanocrystals (lower loadings). The binding energy between CO and Pd increases for a larger Pd particle size, which causes the CO molecule to be more strongly adsorbed to the Pd surface. It is hence further reduced to CH_4_.^84^

Photothermal catalysis is still in its relative infancy. Considerable research and development efforts are still necessary to generate knowledge about the relationships between catalyst composition, size, shape, electronic structure and support with the observed catalytic rates, product selectivity, photon utilization, thermal transport properties, and quantum yield. Additional difficulties arise to accurately measure the local temperature of the catalytic sites, due to various parameters that govern the temperature evolution and distribution within the catalyst bed, including photon penetration, thermal properties of the gas and catalyst, and the thermal energy released or consumed by the chemical reaction (heat is consumed (endothermic) or produced (exothermic) during reactions, respectively, which can influence the local temperature at the active site). Integrating photothermal heterogeneous catalysts successfully into photoreactors will require further research, enabling photons to be efficiently brought into contact with photothermal catalysts while minimizing heat losses.

It should be noted (as will be discussed in Section 4.2) that direct hydrogenation of CO_2_ is thermodynamically less favorable as compared to CO‐rich syngas hydrogenation. The incorporation of a separate RWGS reaction step into the process furthermore represents an additional reaction step including reactor and peripheral components, which requires higher investment cost than direct CO_2_ hydrogenation.

However, the so‐called “CAMERE” (CO_2_ hydrogenation to form MeOH via RWGS reaction) process revealed a relative 20% higher yield when CO_2_ was converted to CO (through RWGS) in a first step, due to the CO feedback effect resulting in improved yields.^25^ Hence, the integration of the RWGS reaction step producing even moderate quantities of CO is beneficial to improve the CO_2_‐to‐MeOH product yield.

High CO_2_ conversion using the RWGS reaction can be achieved at high temperatures, higher than 500 °C. Process waste heat of such high temperature is generally not readily available in common commercial processes (the temperature level of the MeOH synthesis reaction is 250 °C).

The are other strategies being explored to reduce the energy demand of the RWGS reaction step. One such approach is the so‐called sorbent‐enhanced RWGS (SERWGS), a process in which a sorbent is added that can selectively remove water from the reactor. This drives the equilibrium toward CO production, and as high as 100% CO can be achieved at temperatures as low as 250 °C.^91^ It is certain that redundancy is needed due to sorbing/desorbing alternation required for this type of a system. Notwithstanding, the authors estimated that with a SERWGS efficiency of 90%, a high‐pressure water electrolysis step supplying H_2_ efficiency of 85% and a PV efficiency of 19%, a solar energy to liquid fuel efficiency of 8.2% is possible. With a CO_2_ capture energy penalty of 150 kJ mol^−1^, the efficiency becomes 7.7%.

#### 4.1.2 Water Electrolysis: Utilized in Strategies A, C, and D

Investigations of MeOH synthesis from CO_2_ and H_2_ are much more common that direct use of water as the H_2_ source. However, this approach requires one to consider the source of H_2_ gas. Currently, most H_2_ gas is produced by SMR discussed earlier. While this process can be combined with recycled CO_2_, it is counterproductive in terms of GHG emissions, as the H_2_ is primarily fossil‐derived. Therefore, the ideal solar synthesis process should incorporate the use of renewable H_2_ through electrolysis, whereby water is dissociated into H_2_ and O_2_ using electrical energy (Equation (5))

(5) $${\mathrm{2H}}_{2}O⇌\;{\mathrm{2H}}_{2}\;+\;O_{2},\;\Delta G_{\mathrm{298}\;K}^{o}=228.7\;\mathrm{kJ}\;{\mathrm{mol}}^{-1}$$

Of course, using this pathway to produce MeOH is not without its own challenges. While the overall reaction of CO_2_ and water to MeOH is endothermic, the synthesis reaction from CO_2_ and H_2_ is exothermic. Therefore, the electrolysis reaction takes over for steam reforming as the most energetically intensive part of the overall process. The working principles and state of the art of the various types of electrolysis have been reported in refs. 92, 93, 94.

The respective half reactions occurring during the evolution of H_2_ and O_2_ are listed in Equations (6) and (7)

Oxygen evolution reaction (OER)—acidic conditions

(6) $$H_{2}O_{\left(l\right)}\to \;1/2O_{2}(g)\;+\;{\mathrm{2H}}^{+}{}_{\left(\mathrm{aq}\right)}\;+\;{\mathrm{2e}}^{-},\;E{}^{\circ}=\;+\;1.\mathrm{23}\;V$$

H_2_ evolution reaction (HER)—acidic conditions

(7) $${\mathrm{2H}}^{+}{}_{\left(\mathrm{aq}\right)}\;+\;{\mathrm{2e}}^{-}\to H_{2(g)},\;E{}^{\circ}=0.\mathrm{00}\;V$$

where the standard reduction potentials are with respect to the reversible H_2_ electrode. The above reactions assume acidic conditions; under basic conditions, the half reactions are listed in Equations (8) and (9).

OER—basic conditions

(8) $${\mathrm{2OH}}^{-}\to 1/2O_{2}(g)\;+\;H_{2}O\;+\;{\mathrm{2e}}^{-},\;E{}^{\circ}=\;-0.\mathrm{40}\;V$$

HER—basic conditions

(9) $${\mathrm{4H}}_{2}O\;+\;{\mathrm{4e}}^{-}\to {\mathrm{2H}}_{2}\;+\;{\mathrm{4OH}}^{-},\;E{}^{\circ}=-0.83\;V$$

#### 4.1.3 Solar‐Assisted Water Electrolysis for the Production of H_2_ Gas

Here we present a brief summary of light‐assisted or photoelectrochemical water electrolysis processes forming H_2_.

*Photoelectrochemical Water Electrolysis*: PEC water electrolysis has been a popular research topic since Honda and Fujishima demonstrated the reaction over TiO_2_ in 1972.^95^ Since then, the mechanisms of the process have been investigated thoroughly.

**Figure 13:**
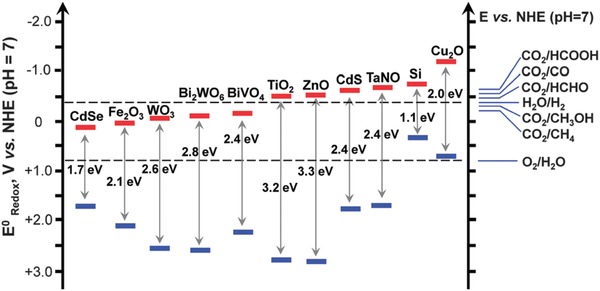
Band alignment of several water splitting catalysts, relative to the reduction potential of the water splitting half reactions. Reproduced with permission.^104^ Copyright 2016, Royal Society of Chemistry.

Semiconductors used to collect light and drive the OER/HER (Equations (6)–(9)) must have valence and conduction band edges that correctly align with the half‐reaction potentials. That is, the conduction band edge of the semiconductor should be more positive than the potential of the OER, and/or the valence band edge must be more negative than the reduction potential of the HER. **Figure** **13** shows the band alignment of several commonly studied semiconductor photocatalysts, relative to the standard reduction potentials of the HER and OER. Photocatalysts for water electrolysis have been reviewed recently.^96^, ^97^, ^98^, ^99^, ^100^, ^101^, ^102^, ^103^

Bolton et al.^105^ classified single‐bandgap and dual‐bandgap water electrolysis systems into type S and type D systems, respectively, and determined efficiency limits on ideal PEC water electrolysis systems. Single‐bandgap systems use either a HER or OER photocatalyst, most often coupled to a nonabsorbing counter electrode, which offers simplicity in design compared to type D (also called Z‐scheme) systems, which use two photocatalysts with complementary bandgaps. The maximum achievable solar‐to‐H_2_ (STH) efficiency is 30.7% for a type S system, and 42.6% for a Z‐scheme system.^105^, ^106^ Additional semiconductor junctions do not improve the theoretical efficiency.^107^

Real PEC systems suffer additional losses from resistances in electronic and ionic conductivity, charge transfer losses, nonideal band alignments of semiconductors, and electron–hole pair recombination. Recombination is particularly challenging to overcome, and remains the focus of a number of studies.^96^, ^108^, ^109^, ^110^, ^111^ Improved charge separation has been achieved through the use of nanostructured catalyst surfaces,^96^, ^112^, ^113^, ^114^ as well as direct contact between reduction and oxidation sites through cocatalysts.^109^, ^115^, ^116^ Electrolyte resistance is also a significant challenge. Nafion is the best solid‐state electrolyte at room temperature, but requires the use of acidic pH, which limits the choice of catalyst material significantly.^102^ Anion‐exchange membranes are used under basic conditions, which allows the use of materials that would degrade at low pH, but generally have lower conductivity.^117^ Vargas‐Barbosa et al. have explored the use of bipolar membranes, which combine Nafion with an anion exchange.^118^ The offered improvements to the system's lifetime are enabled by allowing the use of favorable pH conditions for each electrode.

For comparison, some existing commercial water electrolysis systems have reached ≈44 kWh kg^−1^ of H_2_ produced, representing ≈81% efficiency in the conversion of electrical energy to H_2_. The most efficient solar panels commercially available at present reach values of ≈22.5%. Therefore, combining the two currently available systems results in an overall STH efficiency of ≈18%. The National Renewable Energy Laboratory maintains the record STH efficiency for a PEC device under controlled conditions at 16%,^119^ indicating that PEC water splitting has the potential to be a feasible option for H_2_ production at scale.

#### 4.1.4 Solar‐Thermochemical CO_2_ and Water Splitting: Utilized in Strategy B

Solar‐thermochemical water splitting utilizes high temperatures generated by concentrated sunlight to drive the thermochemical splitting of water into H_2_ and O_2_ (Equation (5)). Temperatures in excess of 3000 °C are necessary to overcome the thermodynamic barrier to dissociate water directly, which results in the need for high light intensities and hence significant solar concentration ratios, and the associated prohibitively high thermal losses.^120^, ^121^ This makes direct thermally driven water dissociation less favorable as compared to other PV–electrochemical or photoelectrochemical methods. Multistep redox cycles have been shown to be a thermodynamically more favorable method to utilize solar heat for splitting water, as the cycles lower the temperature requirements significantly.^120^, ^122^ Conceptually, high‐temperature water electrolysis is similar to thermochemical CO_2_ splitting and syngas production.

Endothermic reactions can be driven by high‐temperature heat using focused sunlight.^123^ The thermochemical splitting of CO_2_ and water via two‐step metal oxide redox reactions operates at high temperatures and utilizes the entire solar spectrum, and thus has been identified as a thermodynamically favorable path to solar fuel production.^124^, ^125^, ^126^, ^127^

The thermochemical redox cycle for the production of solar CO, H_2_, or syngas is represented by Equations (10)–(12)

High‐temperature reduction of a metal oxide

(10) $${\mathrm{MO}}_{x}\to {\mathrm{MO}}_{x-\delta }\;+\;\frac{\delta }{2}O_{2}$$

Low‐temperature oxidation with water

(11) $${\mathrm{MO}}_{x-\delta }\;+\;\delta H_{2}O\to {\mathrm{MO}}_{x}\;+\;\delta H_{2}$$

Low‐temperature oxidation with CO_2_

(12) $${\mathrm{MO}}_{x-\delta }\;+\;\delta {\mathrm{CO}}_{2}\to {\mathrm{MO}}_{x}\;+\;\delta \mathrm{CO}$$

where δ represents the nonstoichiometry in the metal oxides and therefore denotes the reduction extent.

A full thermochemical redox cycle consists of two separate half cycles. The first high‐temperature solar endothermic reduction step occurs at 1450–1600 °C. During this step, O_2_ is evolved, and the metal oxide is partially reduced to a nonstoichiometric or metallic state. Subsequently, the temperature is lowered to 700–1200 °C, and the reduced metal oxide is brought into contact with water and/or CO_2_ to generate H_2_ and/or CO, respectively. This event, during which the metal is reoxidized, is an exothermic process. Solar fuel such as H_2_ or CO can be produced by periodically alternating between the high‐temperature reduction and low‐temperature oxidation steps.

Although isothermal processes have been studied,^128^ most redox cycles operate in a temperature cycling mode. High‐temperature redox cycles have the benefit of inherently separating the product gases, preventing the formation of explosive H_2_/O_2_ mixtures and eliminating the need to separate the products, but are challenged by some level of efficiency loss due to additional process steps.^120^

Another approach that utilizes high‐temperature redox cycles is a method to improve the RWGS reaction described in Section 4.1.1. Wenzel looked at the solar‐thermal cycling of iron and iron oxide,^129^ however, with the reductant being H_2_ rather than simply solar heat. They called this process RWGS chemical looping. The three key reactions are shown in Equations (13)–(15)

RWGS

(13) $$H_{2}\;+\;{\mathrm{CO}}_{2}⇌H_{2}O+\mathrm{CO},\;\Delta H_{R,\mathrm{1073}\;K}=\mathrm{36}.\mathrm{8 kJ}\;{\mathrm{mol}}^{-1}$$

Oxidation

(14) $$\frac{3}{4}\mathrm{Fe}\;+\;{\mathrm{CO}}_{2}⇌\;\frac{1}{4}{\mathrm{Fe}}_{3}O_{4}\;+\;\mathrm{CO},\;\Delta H_{R,\mathrm{1073}\;K}=8.98\;\mathrm{kJ}\;{\mathrm{mol}}^{-1}$$

Reduction

(15) $$\frac{1}{4}{\mathrm{Fe}}_{3}O_{4}\;+\;H_{2}\;⇌\;\frac{3}{4}\mathrm{Fe}\;+\;H_{2}O,\;\Delta H_{R,\mathrm{1073}\;K}=27.86\;\mathrm{kJ}\;{\mathrm{mol}}^{-1}$$

**Figure 14:**
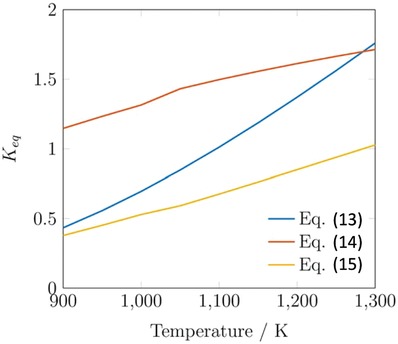
Equilibrium constants (*K*_eq_) for three main reactions, Equations (13)–(15). Reproduced with permission.^129^ Copyright 2016, John Wiley and Sons.

As shown in **Figure** **14**, the idea here is that higher CO_2_‐to‐CO conversion is possible, which can result in high CO:CO_2_ ratios, together, however, with a lower reduction H_2_‐to‐water efficacy than the standard RWGS. Nevertheless, because H_2_–water separation is easier than CO–CO_2_, achieving higher yields of CO in the former is better from an overall efficiency standpoint, as it eliminates the costly CO–CO_2_ separation step.

*Materials for Thermochemical Redox Cycles*: Several redox‐active metal oxides have been studied for thermochemical CO and/or H_2_ production. These include iron oxide, zinc oxide, cerium oxide, and perovskites. Iron oxide has already been demonstrated for thermochemical cycles in the 1970s.^130^ For the case of iron, the oxidation and reduction reactions between Fe^2+^ and Fe^3+^ of FeO, Fe_2_O_3_, and Fe_3_O_4_ are typically used.^130^, ^131^ For zinc oxide, the high‐temperature reduction of Zn^2+^ in ZnO to metallic zinc showed promising results, and besides basic research,^120^, ^132^ the system was also studied up to pilot scale.^133^ However, several challenges exist such as the high‐temperature range, phase transition, and the reactivity of metal vapors. For those reasons, other metal oxides, which remain solid and in the same phase during the whole thermochemical cycle, have been receiving increasing attention.

Cerium oxide fulfills these requirements as a reactor material, as both the oxidized and reduced forms of CeO_2−_ *_δ_* have the same crystal structure. Numerous studies were published on ceria‐based systems on efficiency,^124^, ^126^, ^127^ doping,^134^, ^135^, ^136^, ^137^ structural design,^138^ and different reactor designs.^139^, ^140^ To date, ceria and its solid solutions represent the state of the art for thermochemical redox cycles and set the benchmark for new material developments for this application. This is due to the high efficiency and high chemical and thermal stability of ceria and its solid solutions.^141^

Perovskite oxides have only recently prompted research interests for thermochemical solar‐to‐fuel cycles. Due to the energetically very stable perovskite structure, which is versatile to structurally accommodate a large variety of elements from the periodic table, these materials exhibit potential advantages as compared to binary metal oxides. Due to their structural and compositional versatility, it is possible to design perovskite oxides with tunable nonstoichiometry, and catalytic and photo‐ and electrochemical properties.^141^, ^142^ First studies on solar‐to‐fuel conversion for perovskites were performed on manganite‐based perovskite compositions^143^, ^144^, ^145^, ^146^ and Y–Fe‐based systems.^147^ Further research is necessary to identify perovskite compositions that exhibit thermodynamic and reaction kinetic properties suitable for thermochemical cycles. The large number of possible compositions and versatility of the perovskite structure make these materials promising for thermochemical redox cycles.

### 4.2 Thermodynamic Implications of Using a CO_2_‐Rich Feed for Thermochemical MeOH Production

**Figure 15:**
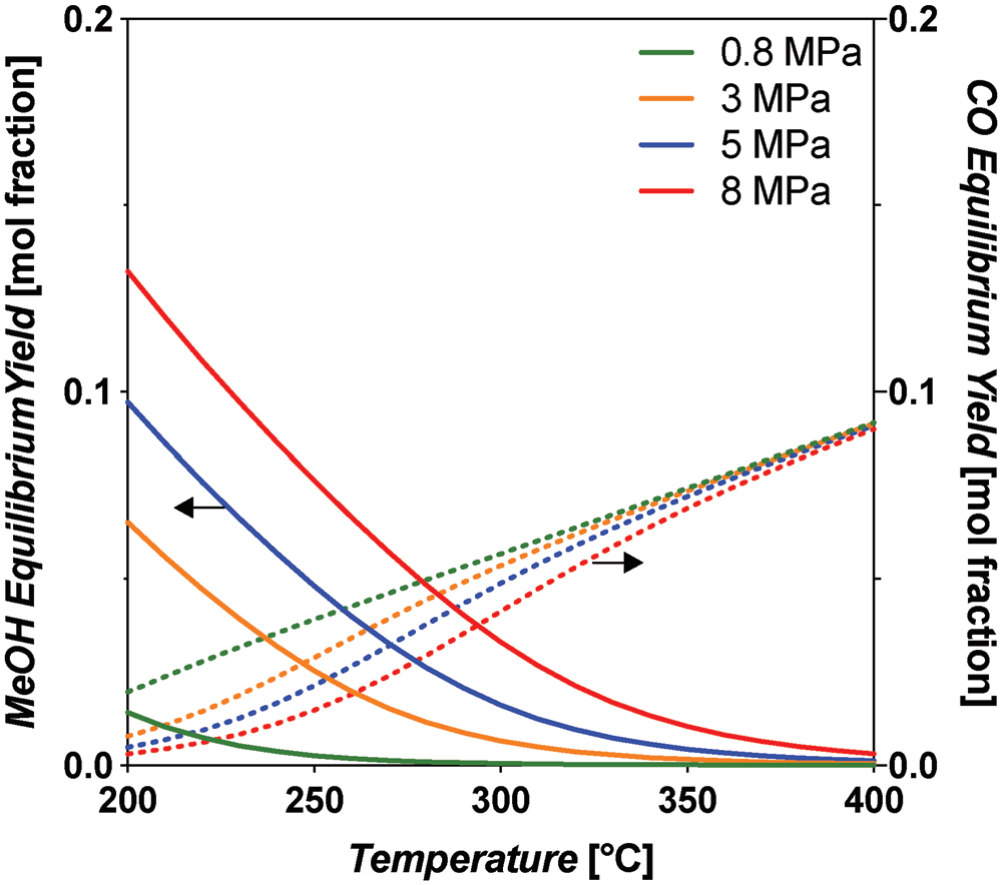
Equilibrium yields of MeOH and CO for a process using CO_2_‐rich feed; in this case, a 3:1 ratio of H_2_ to CO_2_ in the feed. Obtained using Aspen Plus V9 equilibrium analysis.

An Aspen Plus V9 equilibrium analysis similar to that in Section 2 was conducted for a CO_2_‐rich feed of 3:1 H_2_:CO_2_, resulting in an SN of 2. The results, shown in **Figure** **15**, indicate that the MeOH synthesis process with this feed favors higher equilibrium yields at low temperatures (becoming comparable to TCST production at ≈150 °C) and high pressures, similar to the CO‐rich case. Notice, however, that the yields for the CO_2_‐rich feed are lower in magnitude compared to the commercial feed case. This is primarily due to the presence of the RWGS reaction occurring in parallel, rather than the forward WGS.

Unlike the WGS reaction (Equation (3)), which consumes the water produced by the CO_2_‐to‐MeOH synthesis reaction (Equation (2)), the RWGS (Equation (13)) produces water and therefore limits the equilibrium yield rather than providing the complementary feedback effect. At 200 °C and a constant pressure of 8 MPa, the yield from the commercial CO‐rich feed case is 58.8 mol%, while at the same conditions using CO_2_‐rich feed, the yield is only 13.2 mol%, declining to 7.6 mol% at 250 °C. Due to the endothermic nature of the RWGS reaction, its propensity only intensifies as the temperature of the process in increased.

Two general strategies exist for increasing the yield in the CO_2_‐rich feed case. First, adding CO to the feed can help facilitate the WGS reaction. Second, the yield can be increased by physically removing water from the system in situ through the use of a membrane reactor.^148^ A similar approach to removing water to drive the reaction forward is combining MeOH synthesis with its dehydration reaction, via the use of a bifunctional catalyst producing DME.

One advantage, however, of using a feed with increased CO_2_ content is the potential to decrease the heat load that must be managed by auxiliary cooling systems in a scaled‐up process due to the endothermic RWGS reaction occurring in parallel. In the case of the commercial‐scale synthesis using CO‐rich syngas, −90.9 kJ mol_MeOH_^−1^ of heat is released when the WGS parallel reaction removes the equimolar by‐product water from the system. In the absence of the WGS reaction, the heat released is reduced to −49.6 kJ mol_MeOH_^−1^. By using a CO_2_‐rich feed, the heat generated in the presence of the RWGS reaction is −8.3 kJ mol_MeOH_^−1^ (if the RWGS and MeOH selectivities are equal).

By controlling the selectivity of the system for the RWGS reaction, the cooling load for the process can be decreased. This comes at the expense of the MeOH yield, so an analysis of this trade‐off is required.

### 4.3 Implications of Using a CO_2_‐Rich Feed for Thermochemical MeOH Production

Key objectives in processing more CO_2_ are to

- transform CO_2_ into CO to augment MeOH yield using commercial catalysts (Sections 4.1 and 4.3.1),
- develop more water‐stable nanostructured catalysts for processing more CO_2_ (Section 4.3.2),
- tune the selectivity of MeOH production versus CO production via RWGS to allow for selective production (Section 4.3.3) or design for thermal‐neutral conditions that could be solar‐assisted (Sections 4.3.3 and 10.1), and
- develop highly active low‐temperature catalysts to take advantage of favorable thermodynamics at low temperatures, possibly solar‐assisted (Sections 4.4 and 5).

#### 4.3.1 Reaction Kinetics Analysis to Determine the Effect of Increased CO_2_ in Feed

To demonstrate the effect that CO_2_ in the feed has on the reaction kinetics of the synthesis process, the E‐Z Solve model described in Section 2.3 was extended to examine feeds with increasing CO_2_ content. This reaction kinetics model adapted from ref. 45 was fitted to experimental results for *P*_CO_:*P*_CO2_ ratios of 0–4.1, and validated against ref. 21 for ratios up to 10 and greater.

**Figure 16:**
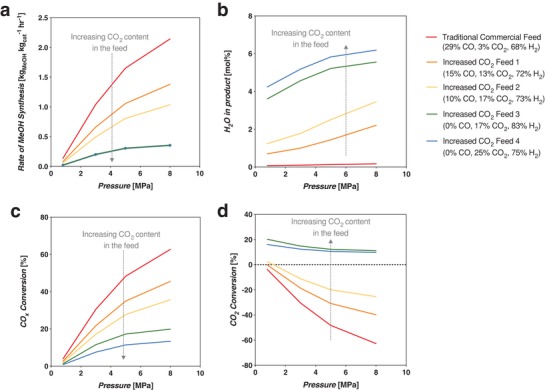
Implications of increasing the mol% CO_2_ in the feed gas for MeOH synthesis. a) Rate via MeOH synthesis reactions decreasing with more CO_2_ in feed. b) Yield of water formed with increasing CO_2_ content in the feed, proportionately increases with increasing CO_2._ c) Feed %CO*_x_* or CO + CO_2_ converted to MeOH with increasing CO_2_ in the feed, less with increasing CO_2_. d) Percent feed CO_2_ conversion (%CO_2_) via RWGS (negative indicates WGS occurring) showing that less and less WGS occurs as CO_2_ increases favoring RWGS and positive CO_2_ conversion

The results of this analysis, shown in **Figure** **16**, indicate that increasing the fraction of CO_2_ in feed

- reduces the MeOH formation rate (Figure 16a),
- increases the mol% of water in the product stream and catalyst bed (Figure 16b),
- decreases the %CO*_x_* conversion toward MeOH (Figure 16c), and
- increases the %CO_2_ conversion from a negative value (producing CO_2_ from WGS) to a positive one (consuming CO_2_ via RWGS) (Figure 16d).

Increasing the amount of CO_2_ in the feed from that of the commercial CO‐rich feed composition (3 mol% CO_2_) to feed 1 (13 mol% CO_2_) leads to a higher %CO_2_ conversion (see **Table** **4**), though a lower rate of MeOH production, lower WGS contribution, and a greater amount of water in the product (2.2 mol% at 8 MPa). These trends continue as the amount of CO_2_ in the feed increases further.

**Table 4:** Selectivity to MeOH, carbon consumption, and STY at different feed compositions, SV: 9900 h^−1^, *T*: 250 °C, *P*: 8 MPa, and catalyst: CZA

Feed composition, CO:CO_2_:H_2_ [mol%]	SN	Selectivity of CO_2_ to MeOH, *Ŝ*_MeOH_ [%]	CO_2_ in feed consumed making MeOH [abs % inlet]	CO in feed consumed making MeOH [abs % inlet]	STY [kg_MeOH_ L_cat_^−1^ h^−1^]
0.0:16.7:83.3	3.99	63.9	3.32	(−1.87%, CO produced)	0.47
0.0:25.0:75.0	2.00	57.7	3.33	(−2.44%, CO produced)	0.47
5.00:20.93:74.07	2.05	100.0	4.08	2.19	0.89
10.00:17.16:72.84	2.05	100.0	2.79	6.92	1.37
15.00:13.40:71.60	2.05	100.0	1.64	11.29	1.82
20.00:9.63:70.37	2.05	100.0	0.78	14.99	2.23
25.00:5.86:69.14	2.05	100.0	0.3	18.03	2.59
29.00:2.85:68.15	2.05	100.0	0.093	19.96	2.83

**Figure 17:**
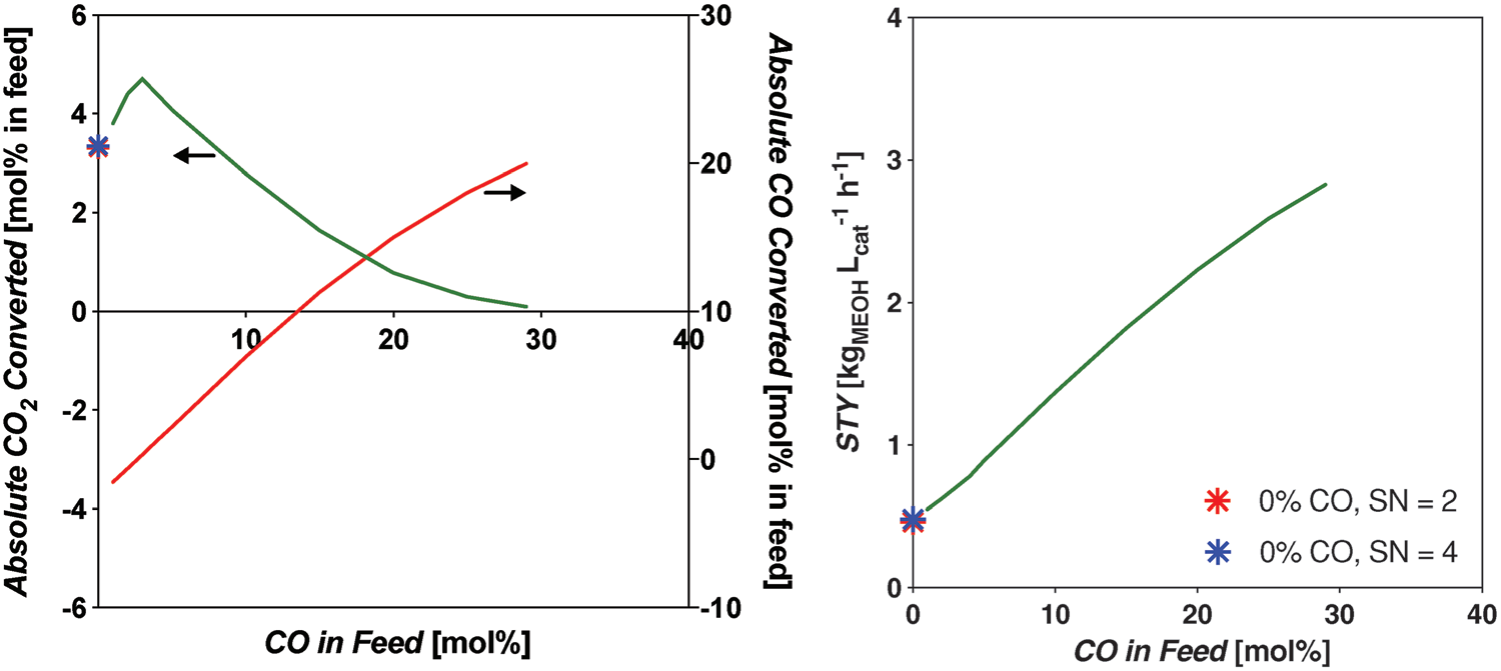
Left: Absolute mol% CO_2_ and CO in feed converted to MeOH as a function of mol% CO in the feed at 8 MPa, 250 °C, and SV 9900 h^−1^. Right: STY as a function of mol% CO in feed (all with an SN of 2.05) at the same conditions.

As the CO content in the feed increases, the carbon in the MeOH product is shown to increasingly derive from CO_2_ generated from the WGS reaction, rather than from CO_2_ initially present in the feed (the previous Table 4 showing a summary of the effect of varying feed composition and **Figure** **17**, left). In the TCST feed case (with minimum CO_2_ in the feed), CO_2_ species in the feed appears to act as a spectator, and its role in the system is relegated to maintaining the optimal CZA activity.

Figure 16b shows that increased CO_2_ content from feed 1 (13 mol% CO_2_) to feed 4 (25 mol% CO_2_) increases the percentage water in the product stream from 2.2 to 6.2 mol% at 8 MPa. Furthermore, there is more than an order of magnitude increase from the CO‐rich case, which has ≈0.17 mol% water in the product.^149^, ^150^, ^151^

The selectivity of the system toward MeOH production is also especially sensitive to increasing the fraction of CO in the feed. Increasing the amount of CO from 0 to 5 mol% increases the selectivity of the system toward MeOH by 42.3%, improving it to 100%. The selectivity remains at 100% when the percentage of CO in the feed is increased from 5 to 29 mol%. The CO_2_ converted increases by 23% from 0 to 5 mol% CO in the feed as well. At 29 mol% CO, the CO_2_ is essentially a spectator. Therefore, at an intermediate 15 mol% CO in the feed, its effect is to decrease the amount of CO_2_ consumed, but offers optimal selectivity and roughly quadruples the rates when a feed composition with 0 mol% CO is utilized.

There is also an increase in selectivity with higher H_2_ concentrations, and the model predicts a 3.5% increase in MeOH selectivity for every increase of 5 mol% H_2_ in the feed. This can be inferred from the rate law as well, showing a dependence of *f*_H2_^3/2^ for MeOH synthesis from CO or CO_2_, and *f*_H2_ for RWGS.^45^

Using feed 1 (15:13:72 mol% CO:CO_2_:H_2_) as a target composition can provide a good compromise by taking advantage of the reduced cooling load requirements of a system with increased CO_2_ content (see Section 4.2) and reduced water load, while maintaining a reasonably high production rate and yield associated with the presence of CO in the feed.

The STY production is a measure industry uses to compare processes as discussed earlier. With the commercial feed, the model predicts a rate of 2.14 kg_MeOH_ kg_cat_^−1^ h^−1^ or STY of 2.83 kg_MeOH_ L_cat_^−1^ h^−1^. This is slightly higher in the range of the quoted commercial‐scale rate values of 0.54–1.55, for various CZA‐based catalyst compositions and similar space velocities (3600–22 000 h^−1^) due to the rate law being for a catalyst that was freshly prepared (see Table 3). Figure 17, right, shows that increasing the CO content of the feed improves the STY_MeOH_ proportionately. It is also evident that the STY is low in the absence of CO.

From a CO_2_ utilization point of view, Figure 17, left, shows there is a maximum in CO_2_ consumption as a function of CO in the feed. The maximum is due to the added CO impeding CO_2_ consumption via RWGS. Comparing an intermediate CO feed 1 (15 mol% CO) to a CO_2_‐rich feed, feed 3 (0:17:83 mol% CO:CO_2_:H_2_), introduced CO_2_ is consumed twice as much as feed 1 (15:13:72 mol% CO:CO_2_:H_2_). If the goal of the process is to consume a maximum amount of CO_2_ as a GHG reduction strategy, it may be economically advantageous to employ a two‐stage reactor, first producing a gas mixture containing 15 mol% CO via the RWGS reaction, and then feeding the resulting mixture of CO:CO_2_:H_2_ to the synthesis reactor. In conclusion, CO remains a vital and valuable feedstock for effective and efficient CO_2_ utilization schemes using the commercial CZA catalyst.

#### 4.3.2 Consequences of Increased Water Content in the Reactor Bed: Catalyst Deactivation

The amplified water content in the catalyst bed as a result of processing more CO_2_‐rich feeds has consequences on the catalyst lifetime. It is found that water deactivates the catalyst over time, and as a result can negatively affect catalyst performance. The water content in the reaction system can reach 5–6 mol% in processes using CO_2_‐rich feeds, while conventional CO‐rich systems experience only ≈0.17 mol% of water in the catalyst bed.

The effect of water on the CZA catalyst can be understood by its effect on the catalyst nanostructure. To quantify the effect of water, the findings of Kuld et al.^152^ are considered. They related the size of the Cu and ZnO nanoparticles to the catalyst activity expressed through its turnover frequency (TOF). They found that the TOF was more sensitive to the size of ZnO particles than the Cu particles, by demonstrating that the TOF increased exponentially with decreasing ZnO particle size, whereas the TOF plateaued with decreasing Cu particle size. This suggests that the high ZnO/Cu interfacial area when ZnO is highly dispersed plays an important role in the CZA catalyst activity, and that the rate is more sensitive to ZnO agglomeration. Behrens et al.^35^ have shown that ZnO not only serves its well established role of stabilizing and spacing Cu islets but also layers on top of Cu particles forming disordered deposits of a few atomic layers in thickness. These more active surface morphologies and interfacing Cu–ZnO sites could occur more frequently as ZnO size decreases. A review summarizing the literature of the active site was conducted by Hansen and Højlund Nielsen^153^ Lunkenbein et al. found that ZnO was the more dynamic phase during a 148‐day deactivation study at industrially relevant conditions (230 °C, 6 MPa, 6:8:59:27 mol% CO:CO_2_:H_2_:inert).^154^ In the two deactivation regimes they found, in the first ≈50 days the ZnO restructuring dominated (densification of ZnO on Cu and Zn, Al–spinel formation) which they associated with loss of oxygen vacancies (ZnO_1−_ *_x_*) as shown by N_2_O reactive frontal chromatography measurements. From 50 to 148 days, they found a “slow” Cu surface area decrease from 10 to 5 m^2^ g^−1^ (which they attributed to loss of interphase and increase in particle size). Their work also showed that ZnO is a cocatalyst, instead of merely a support preventing Cu sintering.

Based on these findings, it is likely water predominantly deactivates the CZA catalyst by causing agglomeration in the ZnO phase, thus removing the availability for the ZnO to provide a beneficial SMSI on the Cu species. However, water has been found to contribute to Cu crystallinity growth as well.^155^

Competing rates of MeOH production and catalyst deactivation need to balance over the catalyst lifetime. In order to maximize the productive lifetime of the commercial CZA catalyst, typically the operating temperature is steadily increased from 250 to +260 °C as the catalyst deactivates. This practice maintains the STY at a constant value while minimizing the rate of catalyst sintering.

In closing, the prospect of introducing feed 1 (15:13.4:71.6 mol% CO:CO_2_:H_2_) as the syngas feed (similar the “CAMERE” process) has the effect of increasing the water content by approximately an order of magnitude (0.2 to ≈2.2 mol% water). This could be a reasonable target for modifying CZA to be more water stable at these levels; however, given the currently affordable laboratory‐grade cost of CZA (US$0.11 g^−1^),^156^ it may make economic sense to continue making use of it (albeit less efficiently) without modification.

#### 4.3.3 Tuning Catalyst Selectivity

An objective listed at the start of Section 4.3 for a CO_2_‐rich catalyst was to tune the selectivity of MeOH production versus CO production via RWGS. This objective can be further subdivided into a) control the selectivity to target products and b) manage the cooling requirements to be moderate or nonexistent.

**Figure 18:**
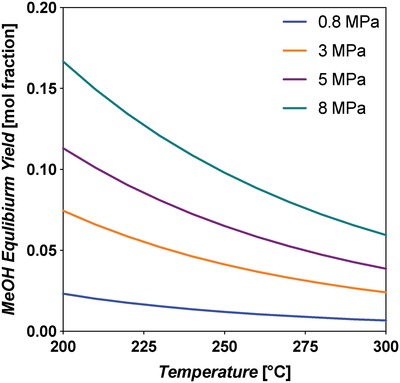
Equilibrium MeOH yield from 3:1 H_2_:CO_2_ feed with no RWGS (Aspen Plus V9, equilibrium reactor block).

For the objective (a), the catalyst could be engineered for the MeOH synthesis reaction (Equation (2)) only. This would have the effect of eliminating the additional water supplied by the RWGS. The main MeOH synthesis reaction without RWGS was modeled by specifying the reaction equation in an equilibrium reactor block in Aspen Plus V9 using the same parameters for the previous equilibrium analyses. If a catalyst can be developed that curtails the RWGS, for CO_2_‐rich feeds there is the potential to take advantage of the higher equilibrium yields of MeOH at higher temperatures compared to the multireaction system. The result of this is shown in **Figure** **18**. Under these conditions, 8.8 mol% MeOH is achievable at 260 °C at 8 MPa, compared to 6.6 mol% for the two‐reaction system (MeOH synthesis and RWGS), or roughly half the typical achievable yield for the CO‐rich CZA system (35–44% CO*_x_* conversion or 14–20 mol% MeOH yield). With higher temperatures up to 300 °C, the main reaction yield is higher at 5.9 mol% versus 3.3 mol% (with RWGS) and the associated rates as well (a typical doubling every 10 °C). Sintering resistant catalysts will be needed to unlock this potential. A highly selective (*Ŝ*_MeOH_ = 50%) MeOH catalyst has been shown at low pressure (0.1 MPa) by Wang et al.^157^

For objective (b), thermochemical MeOH synthesis from a CO_2_‐rich feed can take advantage of the parallel exothermic MeOH synthesis reaction (Equation (2)) and endothermic RWGS reaction (Equation (13)), by balancing the activity by tuning the selectivity between the CO and MeOH reaction products to achieve a thermal‐neutral process. There exists a thermal‐neutral temperature for the CZA catalyst for CO_2_‐rich feeds when the RWGS is the preferred direction, where heats of reaction cancel out and the system is thermoneutral.^148^ This temperature is near 340 °C for CO_2_:CO*_x_* ratios in the feed between 0.25 and 0.5. As such, this applies to CO_2_‐rich feeds predominantly. Thermal energy from light or localized heat as a result of the photothermal effect could be used to maintain this temperature; however, the benefit of this is doubtful due to low oxygenate yield and thermal stability issues of current commercial CZA catalysts above 300 °C. If a catalyst could be developed that could take advantage of the thermal‐neutral point, a potential solar process could be developed whereby solar energy could be harnessed to maintain the system at reaction temperature only compensating for heat losses. This would make an otherwise exothermic system of reactions able to be potentially sustained by renewable solar heat.

The selectivity needed for a thermal‐neutral MeOH‐from‐CO_2_ and RWGS catalyst is 55:45% CO:MeOH, and occurs near 4 MPa for the CZA catalyst. At 8 MPa, the selectivity is better to MeOH with the CZA catalyst at 42:58% CO:MeOH, but further from the thermal neutral point. If the selectivity of a catalyst could be tuned to be 55:45% CO:MeOH at high pressure, then MeOH could be produced at the associated higher rates and with heat input via solar heat only. The generated CO could be separated and used to augment H_2_ production via a separate WGS step.

Developing catalysts with tuned dual‐reaction capability would need to be optimized for oxygenate yields at the thermal‐neutral temperature. This can be achieved by developing a catalyst that has a RWGS rate 1.2× faster compared to MeOH at 250–260 °C, at 55:45% selectivity for CO:MeOH. Kattel et al.^158^ have described recent progress of tuning selectivity on metal/oxide interfaces where CO_2_‐to‐MeOH formation is proposed to occur.^159^

Enabling catalysts to have WGS capability may be beneficial to unlock the higher thermodynamic equilibrium yields of the two‐reaction system. However, drawbacks of the TCST process include the high capital cost of producing CO and H_2_,^22^ the dangers of utilizing CO, and low non‐fossil‐derived CO*_x_* converted. A non‐fossil RWGS stage could be an option to utilize CO_2_ directly as CO, but H_2_ must be conserved well enough due to the additional H_2_ demand in addition to that needed for subsequent MeOH synthesis.

### 4.4 Other Thermochemical Methods

Another key objective listed at the start of Section 4.3 is to develop catalysts that exhibit low‐temperature activity with comparable rates to commercial CZA catalysts at higher temperatures. For a MeOH‐synthesis‐only catalyst at moderate temperatures of 200 °C, maximum yields of 16.6 mol% are possible with CO_2_‐rich feeds (Figure 18). If the reaction could be driven toward equilibrium by removing water, this would allow comparable yields to current CO‐rich feeds and commercial catalysts. Such a catalyst would need to be water stable, and if operating at lower rates, the process would need a not unreasonable amount of earth‐abundant and affordable catalyst.

For ease of integration with existing thermochemical systems, most of the technologies discussed herein focus on the use of heterogeneous catalysts. Some recent efforts to moderate the currently high temperature and pressure of the commercial process are to embrace new catalysts for solving these issues and are exemplified by substituting Cu with other metals like Au, Ni, and Pd–Cu alloy,^160^ or using ZrO_2_ instead of Al_2_O_3_ as the support, aided by various oxide promoters (CeO_2_, Ga_2_O_3_, La_2_O_3_, MnO, MgO, etc.).^161^ The future of thermochemical production of MeOH from CO_2_ will certainly continue to depend on the development and improvement of such metal and metal oxide systems, but here we also would like to review a class of nonmetal nanomaterials, which stands out from the broadly studied metal‐containing catalysts.

**Figure 19:**
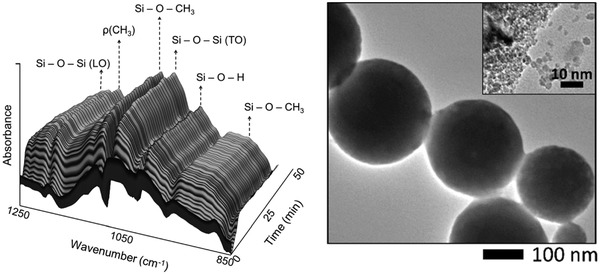
Left: In situ monitoring of FTIR peaks associated with Si—O—Si, Si—O—H, Si—O—CH_3_, and C—H. Right: Si nanoparticles obtained via magnesiothermic reduction. The inset shows the porous nature of the particle. Reproduced with permission.^166^ Copyright 2017, Royal Society of Chemistry.

Silicon, as the second most abundant element in the Earth's crust, has great advantage over rare or expensive elements in being employed in catalyst materials. The large surface area and reducing power of the surface hydride of nanosize silicon contribute further to the great reactivities,^162^ especially toward CO_2_ conversion to fuels, not observed with the bulk silicon. Sun et al. discovered that the hydride‐terminated nanosilicon could heterogeneously reduce gaseous CO_2_ selectively to CO with heating at low pressure.^163^ Dasog et al. first observed acetal group formation at high pressure with hydride‐terminated silicon nanocrystals,^164^ which yielded HCHO, as reported by others employing silicon.^165^ The hydride was consumed, while Si—O—CH_3_ emerged over time, accompanied by oxidation of Si (**Figure** **19**, left). They later demonstrated MeOH could be produced at a minimum temperature and pressure of 100 °C and 1 MPa, respectively, with the porous Si nanoparticles synthesized by reduction of sand using Mg.^166^ The porous structure in Si (Figure 19, right) possibly trapped CO_2_ for efficient H_2_ transfer toward MeOH formation.

Unfortunately, current observations confirmed the conversion of CO_2_ was stoichiometric rather than catalytic. However, unlike MeOH formation using molecular silanes with other catalysts like N‐heterocyclic carbenes,^167^ nanosilicon itself obtained from a grain of sand can thermally reduce CO_2_. With further development, however, it may be possible to transform these silicon nanoparticles into truly catalytic materials. Future efforts toward solar fuel production involving the well‐known efficient absorption of sunlight and the strong photothermal effect,^163^, ^168^, ^169^, ^170^ doping nanosilicon with supporting or active elements,^171^, ^172^ and silicon's versatility to structurally combine with other catalytic materials,^173^, ^174^, e.g., in a manner analogous to making graphene composite catalysts for CO_2_ reduction,^175^, ^176^ may render nanosilicon very promising for catalytic solar MeOH production.

## 5 Photochemical MeOH Production: Strategies D and E

The prospect of using solar energy to drive large chemical processes is an aspirational goal to enable energy and chemical industries to be more sustainable, owing to the decreased reliance on fossil fuels and carbon emissions such a scheme could afford.

In this section, we review several approaches that incorporate light by direct activation of the reaction through absorption of photons. First, we will discuss the theory of how semiconductor photocatalysts operate. Thereafter, we will review both heterogeneous and homogeneous photochemical catalysts starting from CO_2_ and H_2_, and subsequently the ideal scenario starting from CO_2_ and water.

### 5.1 Theory of Photochemical or Photocatalytic Materials

**Figure 20:**
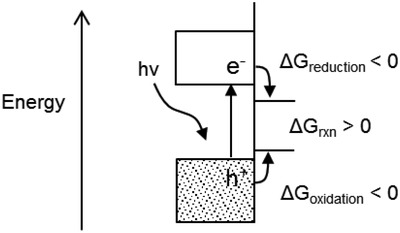
Relative Gibbs free energies of the reduction, oxidation, and overall reaction for a photocatalytic reaction. Reproduced with permission.^177^ Copyright 2011, Elsevier.

Photochemistry can utilize the energy of photons in a chemical reaction by absorbing light to generate excited charge carriers. Photocatalysts can facilitate endergonic reactions through a combination of surface oxidation and reduction reactions. As discussed by Ohtani, endergonic reactions can proceed through the additional input of light energy because the electron and hole pair produced from light absorption of a semiconductor can facilitate two energetically favorable reactions: the oxidation and reduction reactions.^177^ **Figure** **20** shows the relative Gibbs free energies of the reduction, oxidation, and overall reaction.

The interpretation presented here for photocatalysis ultimately implies that an electrochemical process occurs on the surface. This suggests that the reaction proceeds via a coupled electric charge transfer and chemical transformation. Furthermore, as with electrochemical cells it can also be the case that the separate redox reactions are carried out on different surfaces, which will depend on the photocatalyst architecture. The author suggests referring to refs. 177, 178, 179 for a thorough review on the topic.

### 5.2 Photochemical Conversion of CO_2_ and H_2_ to MeOH: Utilized in Strategy D

Photochemical hydrogenation of CO_2_ is promising because it is more thermodynamically favored and avoids many competing reaction problems when using water.^5^ Hydrogenation of CO_2_ using H_2_ and light is, so far, the more thermodynamically favorable route compared to the direct CO_2_ and water to chemical products such as MeOH; this can be seen by comparing the free energy diagram of Figure 6, right, with the standard Gibbs free energy given in Equation (16). Herein, we will discuss solar‐assisted heterogeneous and homogeneous routes for producing MeOH from CO_2_ and H_2_

(16) $${\mathrm{CO}}_{2}\;+\;{\mathrm{2H}}_{2}O\;\to \;{\mathrm{CH}}_{3}\mathrm{OH}\;+\;\frac{3}{2}O_{2},\;\Delta G_{\mathrm{298}}^{o}=\mathrm{702}\;\mathrm{kJ}\;{\mathrm{mol}}^{-1}$$

#### 5.2.1 Heterogeneous Gas‐Phase Photocatalysts

Tsai et al. developed a Ni/NiO core–shell structure over N‐doped InTaO_4_.^180^ In their work, they compared the InTaO_4_ compounds, with and without the additional cocatalyst and N doping. They demonstrated that increasing the level of N‐doping increased the amount of MeOH produced. They also reported a production rate of 0.2 mmol_MeOH_ g_cat_^−1^ h^−1^.

Despite growing activity in the field and encouraging results from computational modeling, very few photocatalysts have been proven experimentally to efficiently and stably hydrogenate gaseous CO_2_ with H_2_ to MeOH at ambient pressure with high selectivity.^5^ It should be noted that selectivity in addition to activity toward MeOH deteriorates with lower pressure and improves with increasing pressure. Recently, it has been reported that a defect‐laden indium oxide, In_2_O_3−_ *_x_*(OH)*_y_*, with a rod‐like nanocrystal structure, photocatalyzed the hydrogenation of CO_2_ to MeOH with 50% selectivity under simulated solar irradiation at a rate of 0.06 mmol_MeOH_ g_cat_^−1^ h^−1^ at 250 °C and atmospheric pressure.^157^ In comparison with In_2_O_3−_ *_x_*(OH)*_y_* nanocrystals, the higher production rate using nanorod structures under solar illumination could be attributed to the prolonged lifetime of excited charge carriers in the nanocrystal superstructures. This catalyst will be considered in strategy D of the TEA.

**Figure 21:**
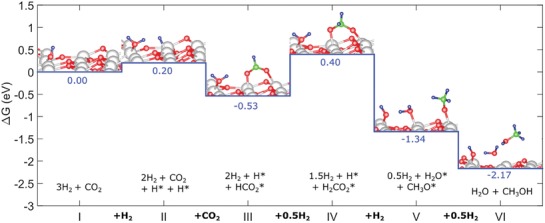
Molecular‐level mechanism for CO_2_ hydrogenation into MeOH. Reaction energy profile from DFT calculations for MeOH production via CO_2_ hydrogenation over In_2_O_3−_ *_x_*(OH)*_y_*(111). Gray atoms denote In, red atoms denote O, green atoms denote C, and blue atoms denote H. Reproduced with permission.^157^ Copyright 2018, Elsevier.

Earlier investigations of the solar‐powered RWGS reaction on In_2_O_3−_ *_x_*(OH)*_y_* proposed that surface frustrated Lewis pairs (SFLPs) formed by a surface hydroxide as Lewis base and a proximal coordinately unsaturated indium site as Lewis acid (denoted InOH∙∙∙In) could be the active sites responsible for the excited state conversion of CO_2_ to CO.^86^, ^90^ A DFT computational analysis was used to investigate the possible CO_2_‐to‐MeOH pathways of the defective laden hydroxylated indium oxide surface via the SFLP site (**Figure** **21**). In the first step, I) H_2_ is heterolytically split on the SFLP and produces a hydride bound to the surface In as an In—H, and a proton bound to the surface InOH group as InOH_2_; II) next, two hydrides interacted with CO_2_ to first form a surface‐bound formate (HCO_2_*), and then an acetal intermediate (H_2_CO_2_*); III) the addition of molecular H_2_ results in the C—O bond splitting, with the proton binding to the carbon fragment to form a methoxide (CH_3_O*) intermediate bound to a neighboring surface In; and IV) the final addition of H_2_ (hydride and proton pair) completes the surface reaction resulting in desorbed MeOH and water.

Although further experimental studies are still required to fully elucidate the reaction mechanism, this distinctive SFLP surface chemistry, in conjunction with the longer carrier lifetimes that arose from the nanocrystal superstructure nature of the material, enabled the remarkably high formation rates observed under ambient conditions.^157^ In the area of metal‐free catalysis, organometallic FLP systems have also been applied in CO_2_ reduction to MeOH, however, without light irradiation.^181^

#### 5.2.2 Homogeneous Photocatalysts

**Figure 22:**
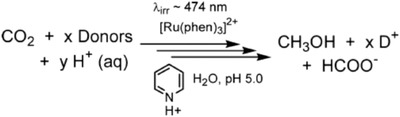
Photochemical catalytic CO_2_ reduction to formate and MeOH in an aqueous homogeneous system at pH 5.0 using visible light irradiation (470 ± 20 nm). Reproduced with permission.^181^ Copyright 2013, American Chemical Society.

The pyridine molecule has been used as a selective electrocatalyst in converting CO_2_ directly to MeOH when a particular electrode (i.e., Pd or Pt) was used.^181^ Boston et al.^181^ have, for the first time, successfully extended this system to a pure photochemical process with visible light irradiation at 470 ± 20 nm by using pyridine as the CO_2_ reductant, [Ru(phen)_3_]^2+^ as the chromophore, and ascorbate as the sacrificial donor (**Figure** **22**). Mechanistically speaking, [Ru(phen)_3_]^2+^ was excited by absorbing light and localized the electron on one of the phen or bpy ligands and the hole on the metal center (i.e., [Ru^III^(phen)_2_(phen^•−^)]^2+^*). Then the pyridinium ion was reduced by the ruthenium chromophore and the hole was quenched by ascorbate. The sacrificial donor plays an important role in realizing this photochemical MeOH production solution, and ascorbate was chosen due to tight pH range limits.

However, the system is inactive after 6 h of irradiation, which appears largely to be due to chromophore degradation. This is a disadvantage of homogeneous MeOH catalysis, which provides good support that the heterogeneous gas‐phase approach is a promising alternative. In addition, formic acid was still the dominant reduction product. Thus, it is important to find more stable chromophores such as metals and metal oxides, and a better catalyst that is more selective for MeOH production. Compared with homogeneous catalysis, heterogeneous systems, especially in the gas phase, are preferable in terms of cost, stability, separation, handling, and reuse of the catalyst, and are common for high‐throughput industrial processes.^5^

**Figure 23:**
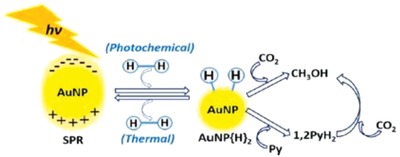
Scheme of the proposed catalytic reduction of CO_2_ to MeOH and Py to 1,2 PyH_2_ by AuNP/AuNP{H}_2_ redox couple. Reproduced with permission.^182^ Copyright 2017, American Chemical Society.

**Figure 24:**
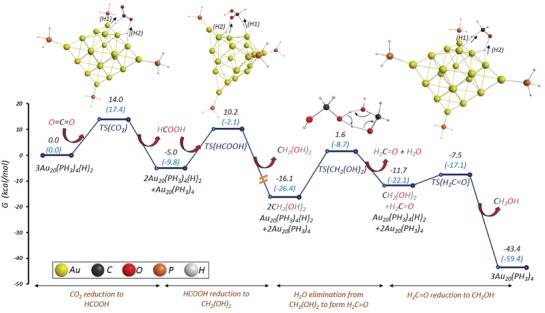
Gibbs free‐energy profile of CO_2_ to MeOH and water conversion process. Reproduced with permission.^182^ Copyright 2017, American Chemical Society.

Ganguly et al. reported computational investigations predicting that hydrogenation of CO_2_ to MeOH on the (111) surface of a 20‐atom Au nanocluster could occur at room temperature without using a sacrificial agent.^182^ Based on this model, the H_2_ molecules physiosorbed onto the gold surface in the ground state. When the gold surface was excited by light absorption, hot electrons (i.e., energetic electrons not in thermal equilibrium with the rest of the material) were generated through the surface plasmon resonance, which assisted H_2_ dissociation. The dissociated H atoms, now at a higher chemical potential, were initially partially negatively charged and could be used to drive several endergonic hydrogenation reactions (**Figure** **23**). During the reaction with CO_2_ to formic acid through the concerted hydride–proton transfer step, the H_2_ bound to O_2_ gradually gained protic character along the reaction pathway. As shown in **Figure** **24**, formic acid underwent two more hydride–proton transfer steps to form hydrated formaldehyde and then MeOH.

**Figure 25:**
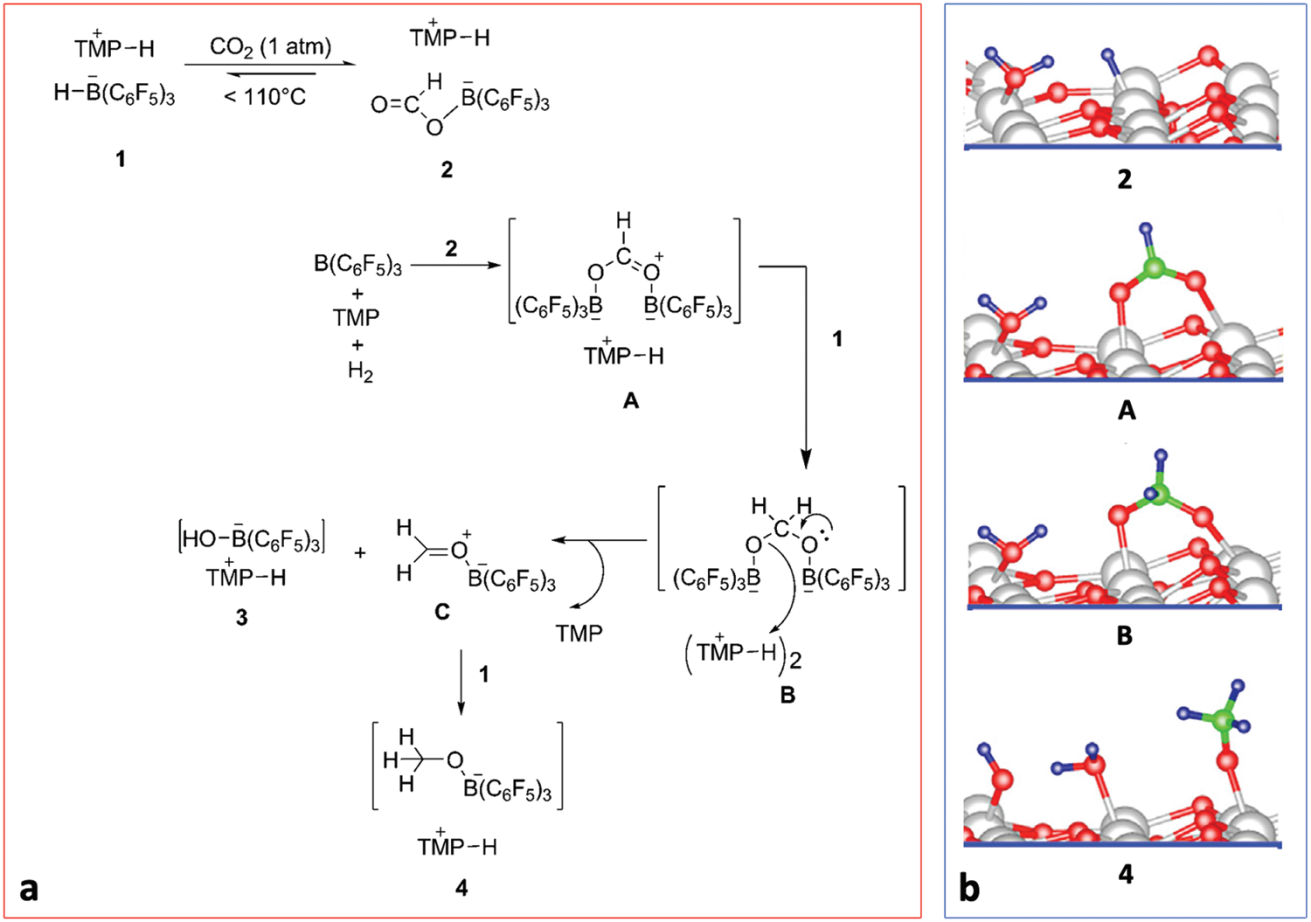
a) Proposed mechanism for the disproportionation of **2** into **4** and CO_2_. (Each intermediate was assigned with a representative number from **1** to **4**.) b) similar intermediate to the In_2_O_3−_ *_x_*(OH)*_y_* system from Figure 21. Gray atoms denote In, red atoms denote O, green atoms denote C, and blue atoms denote H (the symbol below each figure represents corresponding intermediates in part (a)). Reproduced with permission.^183^ Copyright 2009, John Wiley and Sons.

In a similar pathway as the In_2_O_3−_ *_x_*(OH)*_y_* system, H_2_ was heterolytically split by the sterically encumbered frustrated Lewis acid–base pair TMP/B(C_6_F_5_)_3_ (TMP = 2,2,6,6‐tetramethylpiperidine) giving [TMPH]^+^[HB(C_6_F_5_)_3_] (intermediate **1** in **Figure** **25**).^183^ CO_2_ then reacts with [TMPH]^+^[HB(C_6_F_5_)_3_] to form formatoborate derivative (intermediate **2**) that subsequently undergoes a stepwise cleavage and reduction leading to a MeOH precursor, a methyl borate derivative (intermediate **4**).

### 5.3 Direct Photochemical Conversion of CO_2_ and Water to MeOH: Utilized in Strategy E

**Figure 26:**
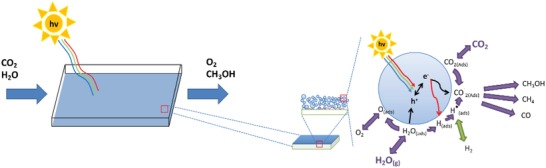
Schematic overview of a direct CO_2_ and water photochemical conversion process. On the left‐hand side, a simplified model of a photocatalyst that absorbs light and activates CO_2_ and water. On the right‐hand side, a representation of the processes involved in the photocatalytic conversion of CO_2_ and water into MeOH and O_2_.

Of all the approaches to directly convert CO_2_ and water into MeOH, the most promising method utilizes heterogeneous, gas‐phase photocatalysis. In this approach, a reaction between CO_2_ and water is facilitated by a photocatalyst in a photoreactor, as shown in **Figure** **26**.

This process is conceived to occur in a single step, wherein a photocatalyst absorbs solar irradiation and then facilitates the conversion of CO_2_ and water vapor into MeOH and O_2_. The reaction between CO_2_ and water is highly endergonic (as shown earlier in Equation (16)), and as such, requires energy input to drive the reaction, which can be provided in the form of light. This can be seen in the case of semiconductor photocatalyst, as defined in Section 1.2.

To drive a photocatalytic reaction, the solar energy input must be sufficient to overcome the thermodynamic and kinetic requirements of the reaction. If the energy input is sufficient, and the reaction proceeds, then the products of this reaction will have captured the solar energy input in the bonds of the product molecules. While MeOH is the focus of this review, there are several other reduction products and reaction intermediates that may also be considered, as shown in Equations (17)–(20)

(17) $${\mathrm{CO}}_{2}\;\to \;\mathrm{CO}\;+\;\frac{1}{2}O_{2},\;\Delta G_{\mathrm{298}\;K}^{o}=257\;\mathrm{kJ}\;{\mathrm{mol}}^{-1}$$

(18) $${\mathrm{CO}}_{2\;}\;+\;H_{2}O\;\to \;\mathrm{HCOOH}\;+\;\frac{1}{2}O_{2},\;\Delta G_{298\;K}^{o}=270\;\mathrm{kJ}\;{\mathrm{mol}}^{-1}$$

(19) $${\mathrm{CO}}_{2}\;+\;H_{2}O\;\to \;\mathrm{HCHO}\;+\;O_{2},\;\Delta G_{\mathrm{298}\;K}^{o}\;=529\;\mathrm{kJ}\;{\mathrm{mol}}^{-1}$$

(20) $${\mathrm{CO}}_{2}\;+\;{\mathrm{2H}}_{2}O\;\to \;{\mathrm{CH}}_{4}\;+\;{\mathrm{2O}}_{2},\;\Delta G_{\mathrm{298}\;K}^{o}\;=\;818\;\mathrm{kJ}\;{\mathrm{mol}}^{-1}$$

#### 5.3.1 Heterogeneous Photocatalysts

The primary advantage of producing MeOH directly from CO_2_ and water is that it can combine the separate water splitting and CO_2_ reduction steps into one step and thereby reduce the number of separate energy conversion steps. As to whether such endergonic processes are viable at large scales, consider the process of plant photosynthesis, in which solar energy ultimately transforms CO_2_ and water into sugar using biological catalysts (enzymes).^184^ Although the reaction between CO_2_ and water to form glucose is highly endergonic (Equation (21)), the process is carried out globally by photosynthetic flora that collectively capture and convert 120 TW of solar energy as reduced carbon.^185^ In terrestrial plants alone, this amounted to ≈13 Gt CO_2_ sequestered annually, as of 2009.^186^ Therefore, it is feasible to drive highly endergonic reactions at significant rates and efficiencies at a global scale

(21) $${\mathrm{6CO}}_{2}\;+\;{\mathrm{6H}}_{2}O\overset{hv}{\to }C_{6}H_{\mathrm{12}}O_{6}\;+\;{\mathrm{6O}}_{2},\;\Delta G_{\mathrm{rxn}}=\mathrm{2880}\;\mathrm{kJ}\;{\mathrm{mol}}^{-1}$$

Wu applied photocatalyst‐coated fiber optics for the CO_2_ and water to MeOH conversion approach.^187^ Cu@TiO_2_ (anatase) composite photocatalysts were used under UV irradiation to achieve a peak rate of 0.45 µmol_MeOH_ g_cat_^−1^ h^−1^ with 1.2 wt% loading of Cu. This catalyst technology is also the basis for strategy E presented in the TEA (Section 7). Additional details regarding catalytic reactor design for this catalyst are presented in Section 8.2.

Herron and Maravelias^188^ investigated the photochemical CO_2_ + water‐to‐MeOH system from a theoretical standpoint. They identified certain process benchmarks that would allow realization of a MeOH minimum selling price (MSP) at or near the fossil‐derived prices. For their analysis, the CO_2_ reduction subsystem depended on activity and per‐pass conversion while the remaining plant was a function of conversion only. Two scenarios were highlighted: for MSPs of US$1 kg_MeOH_^−1^ and US$0.6 kg_MeOH_^−1^. For the former, a minimum benchmark of 2.5 mmol g_cat_^−1^ h^−1^ and 40–70% conversion was determined corresponding to a solar‐to‐fuel (η^SFE^) efficiency of 2%. For the latter, a 20 mmol g_cat_^−1^ h^−1^ and 70–85% conversion was determined, or a η^SFE^ of 15% (or 1.3‐fold the MeOH market price they considered of US$0.44 kg_MeOH_^−1^).

## 6 PV–Electrochemical and Photoelectrochemical MeOH Production: Strategy F

Both PV–electrochemical and photoelectrochemical technologies have the potential to close the carbon cycle if powered by solar energy.^189^ In these processes, both CO_2_ and water can be converted at the electrode surface, with H_2_, alcohols, and hydrocarbons being considered as net products.^190^ With in‐depth understanding of reaction thermodynamics, kinetics, and infrastructure, the transformation of CO_2_ into chemical precursors or high‐energy‐density liquid fuels (e.g., MeOH) via PV–electrochemical or photoelectrochemical technologies offers the great potential of large‐scale and long‐term energy storage.^189^, ^190^

In this section, we review these technologies that incorporate solar electricity (via PV) electrochemically, and solar electricity (via PV) and light photoelectrochemically, in the production of MeOH. We will discuss the PV–electrochemical approach, and thereafter the photoelectrochemical approach both starting from CO_2_ and water.

The synthesis of MeOH from CO_2_ and water is challenging especially relative to the synthesis of H_2_, CO, or HCOOH, as it requires the transfer of six electrons.^191^, ^192^, ^193^, ^194^, ^195^, ^196^, ^197^ Furthermore, there is always the question of whether the product originates from the CO_2_ feed or from contaminant C‐containing species, which could result in spurious MeOH yields. Therefore, a ^13^CO_2_ isotope experiment is highly desirable for all CO_2_‐based catalytic experiments.^194^, ^196^, ^198^, ^199^ However, owing to the difficulty in synthesizing MeOH and the expense of ^13^CO_2_ feed gas, incorporation of ^13^C labeling into experiments has been significantly hindered. Some examples of ^13^CO_2_ tested catalysts that could efficiently convert CO_2_ and water into MeOH are presented in the following subsections.

### 6.1 PV–Electrochemical MeOH Production: Utilized in Strategy F

The electrochemical reduction of CO_2_ is an effective and scalable technology for the production of solar MeOH. With renewable electricity from solar energy becoming more abundant, the PV–electrochemical reduction of CO_2_ is becoming a sustainable approach.

In general, the electrochemical reduction of CO_2_ offers several advantages including i) ambient reaction conditions, ii) renewable electricity from solar energy, and iii) numerous products such as methane, CO, formic acid, MeOH, ethylene, and higher hydrocarbons.

Around 16 different organics, one of which is MeOH, are accessible by the electrochemical reduction of CO_2_. Balanced redox reactions for the formation of some of these organics are given in Equations (22)–(28)

(22) $${\mathrm{CO}}_{2(g)}\;+\;{\mathrm{2H}}^{+}\;+\;{\mathrm{2e}}^{-}\to {\mathrm{HCOOH}}_{\left(l\right)},\;E_{0}=-0.250\;V_{\mathrm{SHE}}$$

(23) $${\mathrm{CO}}_{2(g)}\;+\;{\mathrm{2H}}^{+}\;+\;{\mathrm{2e}}^{-}\to {\mathrm{CO}}_{\left(g\right)}\;+\;H_{2}O_{\left(l\right)},\;E_{0}=-0.106\;V_{\mathrm{SHE}}$$

(24) $${\mathrm{CO}}_{2(g)}\;+\;{\mathrm{4H}}^{+}\;+\;{\mathrm{4e}}^{-}\to {\mathrm{CH}}_{2}O_{\left(l\right)}\;+\;H_{2}O_{\left(l\right)},\;E_{0}=-0.070\;V_{\mathrm{SHE}}$$

(25) $${\mathrm{CO}}_{2(g)}\;+\;{\mathrm{6H}}^{+}\;+\;{\mathrm{6e}}^{-}\to {\mathrm{CH}}_{3}{\mathrm{OH}}_{\left(l\right)}\;+\;H_{2}O_{\left(l\right)},\;E_{0}=0.016\;V_{\mathrm{SHE}}$$

(26) $${\mathrm{CO}}_{2(g)}\;+\;{\mathrm{8H}}^{+}\;+\;8e^{-}\to {\mathrm{CH}}_{4(g)}\;+\;{\mathrm{2H}}_{2}O_{\left(l\right)},\;E_{0}=0.169\;V_{\mathrm{SHE}}$$

(27) $${\mathrm{2CO}}_{2(g)}\;+\;{\mathrm{12H}}^{+}\;+\;{\mathrm{12e}}^{-}\to \;C_{2}H_{4(g)}\;+\;{\mathrm{4H}}_{2}O_{\left(l\right)},\;E_{0}=0.064\;V_{\mathrm{SHE}}$$

(28) $${\mathrm{2CO}}_{2(g)}\;+\;{\mathrm{12H}}^{+}\;+\;{\mathrm{12e}}^{-}\to C_{2}H_{5}{\mathrm{OH}}_{\left(l\right)}\;+\;{\mathrm{3H}}_{2}O_{\left(l\right)},\;E_{0}=0.084\;V_{\mathrm{SHE}}$$

Notably, since the earliest 1986 report of electrochemical reduction of CO_2_ to MeOH, which employed a molybdenum electrode, only a few materials, mainly metals and alloys, can enable this conversion.^194^, ^195^, ^198^, ^200^, ^201^, ^202^, ^203^, ^204^, ^205^, ^206^, ^207^, ^208^ A promising new material in this regard focuses attention on a molybdenum–bismuth bimetallic chalcogenide (Mo–Bi BMC) composition with nanosheet morphology. It reduces CO_2_ to MeOH electrochemically with high efficiency and selectivity.^198^

The catalyst, prepared via a one‐pot solvothermal reaction, was mixed in ethanol with Nafion, and then coated onto carbon paper (CP) to obtain the BMC/CP electrode. The experiment was conducted in a H‐type cell with an electrolyte of CO_2_‐saturated anhydrous acetonitrile (MeCN) with 0.5 m 1‐butyl‐3‐methylimidazolium tetrafluoroborate ([Bmim]BF_4_) under ambient conditions. The ionic liquid, [Bmim]BF_4_, played different roles in this MeOH producing system: i) increased the electrical conductivity of the electrolyte, ii) enhanced the CO_2_ solubility, and iii) stabilized the intermediate radical anion, CO_2_^•−^.

Several CO_2_ reduction products were detected at different applied potentials, including H_2_, methane, CO, and MeOH. The best Faradaic efficiency of CO_2_ to MeOH was observed at −0.7 V (vs SHE), namely, 71.2% with a current density of 12.1 mA cm^−2^. Proof of product was obtained using ^13^CO_2_ isotope labeling with ^13^C NMR detection of ^13^CH_3_OH.

**Figure 27:**
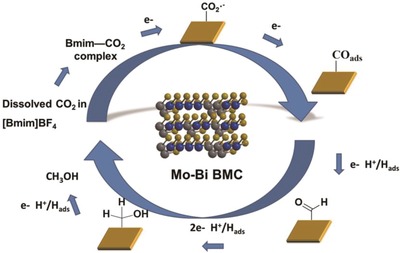
Schematic pathway for electrocatalytic CO_2_ reduction to MeOH over a molybdenum–bismuth bimetallic chalcogenide electrode. Reproduced with permission.^198^ Copyright 2016, John Wiley and Sons.

To probe the mechanism of CO_2_ reduction on the Bi–Mo BMC electrode, the composition of the electrolyte phase at different reaction times was probed by infrared spectroscopy. Diagnostic fingerprint modes at 1085, 1140, and 1420 cm^−1^ were assigned to C—O stretching and CH_3_ deformation vibrations, respectively. The intensity of the CH_3_ fingerprint mode increased with reaction time signaling the formation of MeOH. The origin of the high selectivity toward MeOH was proposed as a synergistic effect between Mo and Bi reaction sites, the Bi sites envisioned to drive the reduction of CO_2_ to CO, while the Mo sites bind CO where it is reduced into MeOH,^195^ the reaction pathway being shown in **Figure** **27**.

To expand on the mechanism, the complex [Bmim–CO_2_]^+^ is formed through the H_2_‐bonding interaction between CO_2_ and the [Bmim]^+^ cation.^209^ The complex so formed is adsorbed onto the Bi–Mo BMC electrode where the H_2_‐bonded CO_2_ is first reduced to CO_2_^•−^ and then to adsorbed CO_ads_, which in a sequence of subsequent electron–proton reduction steps passes through intermediates CHO_ads_ and CH_2_O_ads_ and results in the formation of MeOH.

Herron and Maravelias^188^ also investigated the PV–electrochemical CO_2_ + water‐to‐MeOH system from a theoretical standpoint. For this system, they looked at the current density and cell potential that yielded a specific MSP of MeOH. They found for a cell potential of 2 V, which they stated is an optimistic yet reasonable target, the solar electricity alone would be US$2.44 kg_MeOH_^−1^. For an optimized current density of ≈200 mA cm^−2^ that minimized capital costs, the MSP remained over fourfold the market price (US$0.44 kg_MeOH_^−1^). At 100% selectivity and efficiency, the solar electricity was found to be US$0.91 kg_MeOH_^−1^. Their conclusion is that solar PV must reach parity with fossil‐derived electricity (US$0.06 kWh^−1^) for the MSP to be US$1 kg_MeOH_^−1^

### 6.2 Photoelectrochemical MeOH Production

In general, photoelectrochemistry studies the effect of exposing the electrode(s) of an electrochemical system to light.^210^ Using an applied electric field, a photoelectrochemical system allows for photogenerated charge carriers to be separated between two electrodes (one or both of which may be photoactive), which can then each catalyze a half reaction to realize overall conversion of CO_2_ to fuels.^211^, ^212^ Splitting electrons and holes spatially can greatly improve the lifetime of these charge carriers, thus potentially increasing the catalytic activity. In other words, photoelectrochemical systems can compartmentalize the discrete steps of the redox processes, while photocatalytic systems rely on them occurring at the same surface or adjacent surfaces. Both photocatalysis and electrocatalysis show promise for use in CO_2_ reduction; photo‐electrochemistry is essentially a combination of those two constituents, and can be expected to show similar performance. Owing to the multi‐electron transfer process of CO_2_ reduction, numerous products are often found, for instance, H_2_, methane, CO, MeOH, and CHOOH (Equations (22)–(28) in Section 6.1). While MeOH is the most desired product, the similarity in potentials for these reactions makes selectivity for this product a challenge. The first studies on the use of photo‐electrochemistry for CO_2_ reduction were first performed in 1978; using a p‐type GaP electrode afforded maximum solar energy conversion efficiencies for combined production of MeOH and formaldehyde equal to 0.97% at −0.8 V and 0.61% at −0.9 V (vs SCE). The MeOH selectivity was about 3.6%.^213^ Unfortunately, the very high reaction barrier (resulting in high overpotentials) and poor MeOH selectivity hindered further development.^20^, ^81^, ^191^, ^192^, ^193^, ^199^, ^214^, ^215^, ^216^

Recently, a detailed photoelectrochemical study was performed over a TiO_2_‐based electrode at −0.6 V (vs Ag/AgCl) with a rate of 0.11 mmol_MeOH_ cm^−2^ h^−1^ and quantum efficiency of 0.95% (typically defined as the number of electrons produced per photon input).^199^ Fluorine‐doped tin oxide was used as the support electrode and coated by TiO_2_ films. The TiO_2_ film was then modified with 3‐aminopropyl‐triethoxysilane and incorporated with Nile red (NR) to form NR@TiO_2_. Finally, Pd nanoparticles were deposited onto the prepared NR@TiO_2_ to form the final photoelectrode, Pd/NR@TiO_2_. During the measurement, CO_2_‐saturated KHCO_3_ was used as the electrolyte, −0.6 V was chosen as the applied potential, and a stability test was performed via 100 continuous cycles of cyclic voltammograms. To further confirm the source of the MeOH, ^13^CO_2_ was used as feedstock and the resulting ^13^CH_3_OH was successfully detected by ^13^C NMR.

**Figure 28:**
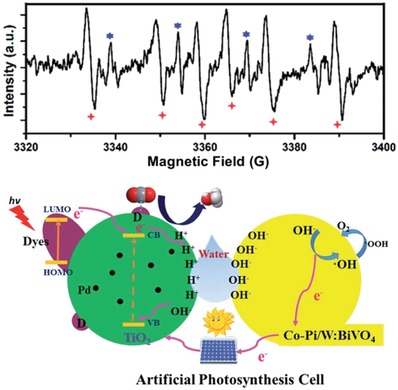
Top: Electron paramagnetic resonance result from catalyst material where *OH marked as blue hexagram and *OOH marked as red star. Bottom: Proposed mechanism for the artificial photosynthesis of MeOH in the photoelectrochemical system. Reproduced with permission.^85^ Copyright 2017, Royal Society of Chemistry.

Electron paramagnetic resonance (EPR) experiments were performed to detect the formation of short‐lived radicals and further understand the mechanism of production. To accomplish the EPR measurements, 5,5‐dimethyl‐1‐pyrroline‐*N*‐oxide (DMPO) was used as a spin trap for OH radicals. EPR detected DMPO—OH and DMPO—OOH, suggesting *OH (blue hexagram) and *OOH radicals (red star) are the main active species for CO_2_ reduction (**Figure** **28**, top). Therefore, a reaction pathway has been proposed in which the CO_2_ molecules were trapped by N atoms and protons were absorbed by decorated Pd nanoparticles (Figure 28, bottom). Meanwhile, the photogenerated electrons from the dye can transfer to the protons, forming H atoms that further reduce CO_2_ to MeOH.

## 7 System Analysis of the Traditional Strategies and Strategies A–F

Based on the discussion of strategies in previous sections, in this section we perform technoeconomic analyses on the six strategies (see Table 1).

All reactions in the six strategies, as well as their selected catalysts for all reactions, corresponding one‐pass conversion, selectivity, operating conditions, and solar‐to‐chemical efficiency (η^S‐C^) are summarized in **Table** **5**.

**Table 5:** Selected catalysts, one‐pass conversion, selectivity, operating conditions, and solar‐to‐chemical efficiency for each reaction (energy for separation not included)

Reaction	Strategy	Catalyst	Conversion [%]	Selectivity [%]	*T* [°C], *P* [MPa]	η^S‐C^ [%]
CO + 2H_2_ ↔ CH_3_ OH with 3% CO_2_ in feed	A, B	CZA^11^	63	100	250, 8	70
CO_2_ + H_2_ ↔ CO + H_2_ O with 3% CO in feed	C	CZA^11^	18	100	250, 8	70
	D	In_2_O_3−_ *_x_*(OH)*_y_* 157	5	100a)	250, 0.1	5
CO_2_ + 2H_2_O → CH_3_OH + 1.5 O_2_	E	Cu@TiO_2_ 217	5	40	25, 0.1	1
	F	Mo–Bi^198^	20	70	25, 0.1	5
H_2_O↔H_2_ + 0.5 O_2_	A, C, D	PEM^218^	100	100	25, 0.1	11
	B	CeO_2_ 127	100	100	1500, 0.1	5
CO_2_ ↔ CO + 0.5 O_2_	B	CeO_2_ 127	80	100	1500, 0.1	5
CO_2_ + H_2_ ↔ CO + H_2_O	A	Ni@Al_2_O_3_ 219	100	100	750, 0.1	70

^a)^ Wang et al. reported a 50% MeOH selectivity with only CO_2_ and H_2_ in feed.^157^ We expect that with 3% CO in the feed, the RWGS side reaction should be counteracted and a higher selectivity similar to that of the CZA catalyst can be achieved.

### 7.1 Process Model

A general solar MeOH synthesis process can be divided into five subsystems.^220^ In subsystem 1, CO_2_ is captured from the source and transported to the MeOH production subsystem 2, where CO_2_ and water are converted to MeOH through one‐ or multistep reactions. The reactor effluent is sent to the gas/liquid separation subsystem 3. The vapor outlet is then sent to the CO_2_ separation subsystem 4, where the unreacted CO_2_ is separated from gas products (e.g., O_2_, methane) and recycled. In this study, membranes instead of cheaper amine‐based CO_2_ separation are used in subsystem 4 due to the need of intermittent operation. Finally, the liquid outlet is sent to the MeOH purification subsystem 5 to achieve the desired product purity.

The main difference among the six strategies lies in the reaction subsystem 2, as shown in Table 5. **Table** **6** summarizes the energy and cost parameters of each subsystem for this analysis.

**Table 6:** Energy and cost (all in US$) parameters of technologies used in each subsystem

Subsystem	Technology	Unit energy use [MJ kg^−1^ of outlet product]			Cost		Ref.
		Power	Heat	Photon	*C*_0_ [US$ million]	${\dot{m}}_{0}$ [kg s^−1^]a)	
1	MEA absorption	0.66	3.09		7.62	4.44	220
2b)	Syngas to MeOH	3.33			23.57	19.70	221
	CO_2_ + H_2_ to MeOH (thermal)	12.4			23.57	19.70	221
	CO_2_ + H_2_ to MeOH (photo)			458	258	77.97	188
	CO_2_ + H_2_O to MeOH (photo)			2288	2461	305.44	188
	CO_2_ + H_2_O to MeOH (elec)	107			712	161.12	188
	H_2_O electrolysis (elec)	180			US$7.2 kg_H2_^−1^		218
	H_2_O splitting (thermal)		1088		2.31	60.6 MW reactor	221
	CO_2_ splitting (thermal)		91				221
	RWGS (thermal)		1.84		7.86	19.70	222
3	Flash tanks	0.20c)			25.24	72.30	d)
4	Membrane	0.30			24.35	28.84	223
5	Distillation		2.1e)		0.69	56.30	220

^a)^ ${\dot{m}}_{0}$ is the total inlet mass flow. For MEA absorption technology, ${\dot{m}}_{0}$ refers to the captured CO_2_

^b)^ Full sensible heat recovery is assumed for all reaction units

^c)^ The inlet pressure of flash tank series is 1 MPa. The compression power of subsystem 3 depends on the pressure of subsystem 2, e.g., if MeOH conversion is at 8 MPa, no power is needed for subsystem 3

^d)^ Based on process calculations performed in Aspen Plus (V8.6)

^e)^ Distillation heating duty depends on inlet MeOH wt% (*x*_MeOH_): $Q_{\mathrm{distil}}=2.1x_{\mathrm{MeOH}}^{-0.706}$.

#### 7.1.1 Energy Analysis

We develop an energy consumption model assuming that all reaction unit operations in subsystem 2 utilize solar energy. The required primary solar energy $\left(E_{i}^{\mathrm{Solar}}\right)$ of unit *i* is proportional to the mass flow rate entering the unit $\left({\dot{m}}_{i}\right)$

(29) $$E_{i}^{\mathrm{Solar}}=\lambda_{i}\;\cdot \;{\dot{m}}_{i}\;\cdot \;\left(\frac{W_{i}}{\eta^{\mathrm{S‐E}}}\;+\;\frac{Q_{i}}{\eta^{\mathrm{S‐H}}}\;+\;E_{i}^{\mathrm{Photon}}\right)$$

where $W_{i},\;Q_{i},\;\mathrm{and}\;E_{i}^{\mathrm{Photon}}$ are the unit power, heat, and photon requirements of unit *i*, respectively, as given in Table 6; η^S‐E^ and η^S‐H^ are the solar‐to‐power and solar‐to‐heat efficiency, given in **Table** **7**; and λ_*i*_ is a factor accounting for intermittent operation (e.g., λ_*i*_ = 0.42 for solar reactors operating for 10 h during daytime).

**Table 7:** Utility generation efficiency and price

Utility	Efficiency	Price [US$ kWh^−1^]
Fossil–power	η^F‐E^ = 37%	0.06
Fossil–heat	η^F‐H^ = 86%	0.01
Solar–power	η^S‐E^ = 16%	0.14
Solar–heat	η^S‐H^ = 45%	0.07

We assume that all separation units in subsystems 1 and 3–5 utilize fossil‐fuel‐derived utilities. The required primary fossil fuel energy $\left(E_{i}^{\mathrm{Fossil}}\right)$ of unit *i* is given as

(30) $$E_{i}^{\mathrm{Fossil}}=\lambda_{i}\cdot {\dot{m}}_{i}\cdot \left(\frac{W_{i}}{\eta^{\mathrm{F‐E}}}\;+\;\frac{Q_{i}}{\eta^{\mathrm{F‐H}}}\right)$$

To assess the energetic feasibility of the process, we use a metric introduced by Herron et al.^220^ called energy incorporation efficiency (EIE), defined as follows

(31) $$\mathrm{EIE}\;=\;\frac{{\dot{m}}_{\mathrm{MeOH}}\cdot {\mathrm{HHV}}_{\mathrm{MeOH}}-\left(\sum_{i}E_{i}^{\mathrm{Fossil}}-{\dot{m}}_{{\mathrm{CH}}_{4}}\cdot {\mathrm{HHV}}_{{\mathrm{CH}}_{4}}\right)}{{\dot{m}}_{\mathrm{MeOH}}\cdot {\mathrm{HHV}}_{\mathrm{MeOH}}}$$

where HHV is the high heating value, which is 22.9 MJ kg^−1^ for MeOH and 55.5 MJ kg^−1^ for methane. Here, we give credit to by‐product methane, if produced, by subtracting its HHV from the total fossil energy consumption. The EIE must be positive for a sustainable fuel, which means that less fossil fuel is consumed than the energy content of the MeOH product.

The primary energy efficiency is defined as the ratio of the product chemical energy to the total primary energy input, including both fossil energy and solar energy

(32) $$\eta^{\mathrm{Primary}}=\frac{{\dot{m}}_{\mathrm{MeOH}}\cdot {\mathrm{HHV}}_{\mathrm{MeOH}}}{\sum_{i}\left(E_{i}^{\mathrm{Solar}}\;+\;E_{i}^{\mathrm{Fossil}}\right)-{\dot{m}}_{{\mathrm{CH}}_{4}}\cdot {\mathrm{HHV}}_{{\mathrm{CH}}_{4}}}$$

**Figure 29:**
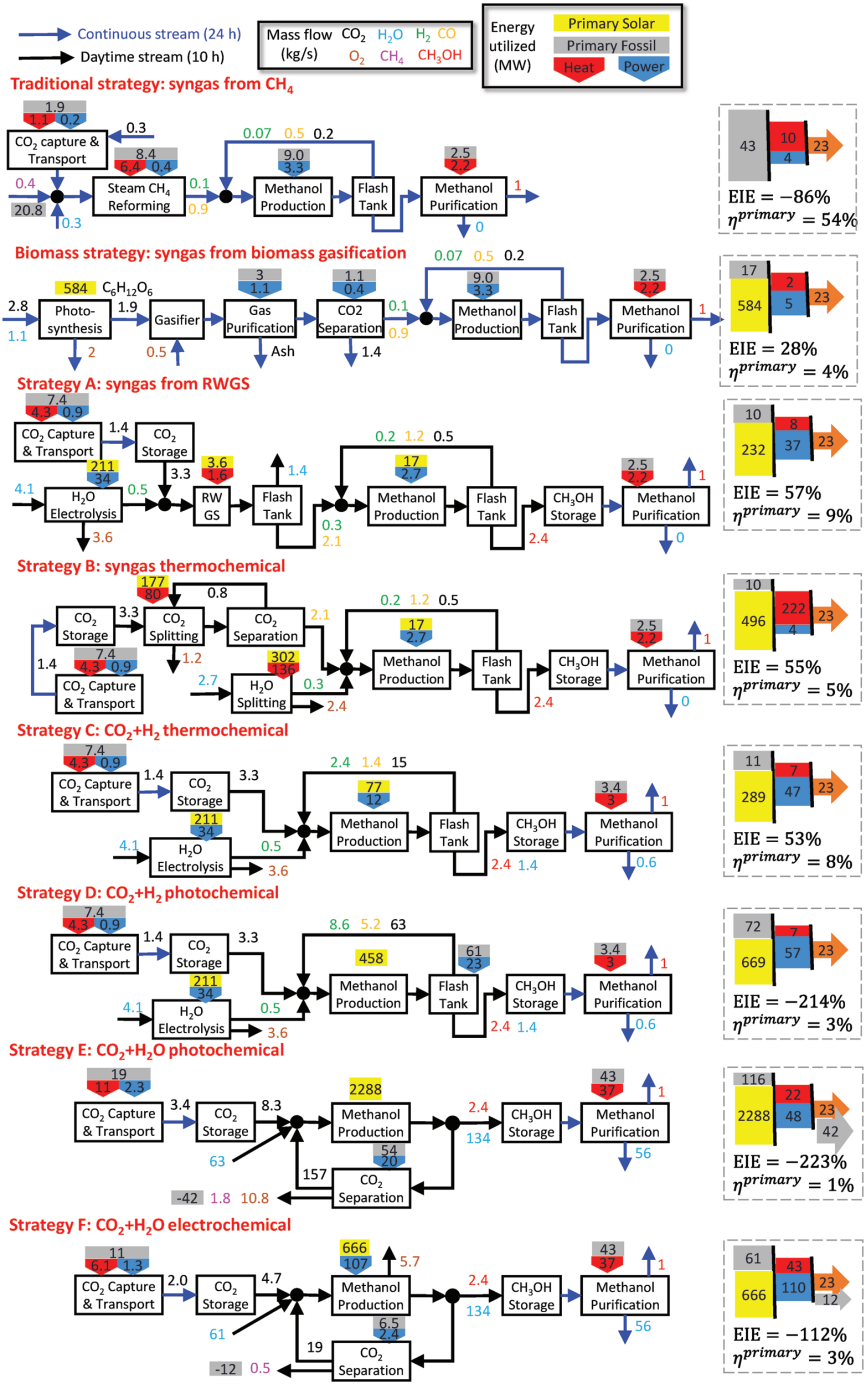
BFDs of different MeOH production strategies. Streams active continuously and during daytime are shown as blue and black arrows, respectively, with mass flows of main components numbered above; process energy utilized by major blocks is shown above corresponding block using the color convention in the label (top). The energy incorporation efficiency and primary energy‐to‐chemical efficiency (η^Primary^) are shown in blocks to the right of each BFD.

**Figure 30:**
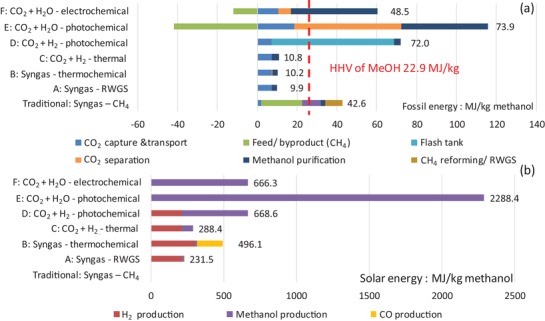
Primary energy usage (MJ kg_MeOH_^−1^) in six strategies and traditional MeOH process: a) fossil energy and b) solar energy.

**Figure** **29** shows the block flow diagrams (BFDs) of the traditional strategy, biomass strategy, and the six proposed solar MeOH strategies. Detailed mass and energy balances are calculated based on the energy model and shown. The solar MeOH process is divided into intermittent and continuous blocks. The solar‐based intermittent block (e.g., solar reactors) only operates during daytime (10 h) while the fossil‐fuel‐based continuous block (e.g., MeOH purification) operates for the entire day (24 h). In order to guarantee continuous operation of process blocks downstream to the intermittent block, intermediates are overproduced and stored during daytime. For example, if the continuous block requires 1 kg s^−1^ inlet flow from the storage tank, the outlet stream of the intermittent block entering the storage tank has to be 2.4 kg s^−1^ during daytime. Therefore, the entire intermittent block has to be overdesigned based on the 2.4 kg s^−1^ and its capital cost is calculated accordingly. For ease of comparison, high‐level primary fossil and solar energy requirements by units are summarized in **Figure** **30**.

The traditional strategy requires 42.6 MJ kg_MeOH_^−1^, with a negative EIE (−86%). The NG feedstock is the major energy driver, accounting for ≈50% of the total energy input. The remaining energy consumption comes mainly from heating and compression for steam reforming at 2 MPa (20%), MeOH production at 8 MPa (21%), and MeOH purification (6%).

The biomass strategy uses CO_2_ instead of NG as feedstock and thus has a positive EIE (28%). However, η^Primary^ is only 4% as a result of the less efficient solar energy uptake in the biomass growth process. Thus, it is important to develop new strategies that enable solar energy to be more efficiently incorporated into chemical products.

The fossil energy consumption in strategies A and B is reduced due to 1) syngas production from CO_2_, instead of methane, and 2) using solar‐derived utilities to drive MeOH synthesis. Although an extra 7.4 MJ kg_MeOH_^−1^ of energy is now required for CO_2_ capture and transportation, the fossil energy usage is reduced by 75% when compared to the traditional strategy, which leads to a positive EIE. The solar energy usage in strategy A is less than 50% of that in strategy B because currently PV–electrochemical water electrolysis has higher solar‐to‐chemical efficiency (11%) than solar‐thermochemical splitting (5%). While the experimental benchmark efficiency for solar‐thermochemical splitting is 5%,^126^ a maximum theoretical efficiency of 29.5% has been calculated if 50% of the sensible heat is recovered.^224^

Strategy C employs the same CZA catalyst for thermochemical MeOH production, but instead of feeding a CO‐rich syngas, it uses a CO_2_‐rich syngas, which eliminates the need for a RWGS unit as in strategy A. However, the CO_2_‐rich syngas has two drawbacks: 1) lower one‐pass conversion (18% vs 63% of using CO‐rich syngas), which leads to high compression cost for recycling the unreacted gas; and 2) higher water production, which leads to higher heating demand for MeOH purification. While strategies A and C use different feeds, they have similar efficiencies. This highlights that the efficiency is buffered by the major contributor to energy demand: H_2_ production (87% and 70% of input energy, respectively). Strategy D has the same process configuration but uses a photocatalyst instead of the CZA catalyst for MeOH production. The photochemical reaction happens at ambient pressure, which due to the low one‐pass CO_2_ conversion (5%), resulting in a high flash tank compression cost (61 MJ kg_MeOH_^−1^) and thus negative EIE.

The direct conversion of CO_2_ and water to MeOH in strategies E and F eliminates the electricity needed for electrolysis. Thus, even though strategy E starts from a less aspirational feedstock, its energy efficiency is comparable to that of strategy D.

The reactions in both systems happen in aqueous solution where MeOH product stays in the solution and the product gas bubbles out. Although this configuration does not require a flash for gas/liquid separation, it has two drawbacks. First, in the gas phase, the unreacted CO_2_ needs to be separated from by‐product methane, which requires high compression power when the CO_2_ conversion and MeOH selectivity are low. Second, the MeOH concentration in the aqueous solution is very low, which leads to high MeOH purification cost. In this analysis, we assume that the MeOH concentration is 1 wt% but acknowledge that this concentration in current experiments is lower. We also note the credit from by‐product methane reducing the net fossil energy consumption. However, considering the energy costs for CO_2_ capture and separation, higher MeOH selectivity should be the future research target in both strategies E and F.

### 7.2 Solar MeOH Process Economics

Energy applications account for 40% of annual MeOH consumption, and are likely to increase in coming years.^22^ The current North American market price for MeOH is US$495 MT_MeOH_^−1^ (June 30, 2018),^225^ or US$0.39 L_MeOH_^−1^. If MeOH from captured CO_2_ is to be competitive with the 75% stake of the market of NG to MeOH^9^ (other sources include coal, biomass, CO_2_), the cost of producing it will need to fall in line with existing technology. Estimates of coal and biomass costs to MeOH versus from NG are two‐ and fourfold, respectively.^9^

**Figure 31:**
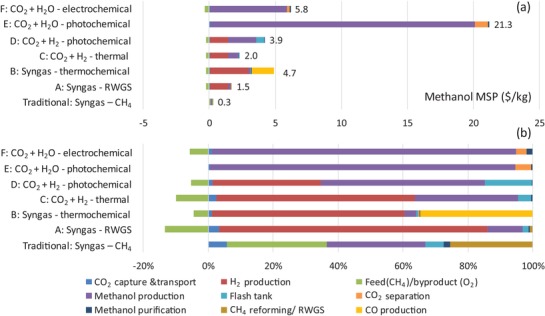
Cost breakdown of MeOH MSP in six strategies and traditional MeOH process: a) MSP (US$ kg_MeOH_^−1^) and b) percentage (%).

To identify the major cost drivers of each strategy, we develop a simple cost model for the estimation of the MeOH MSP. The major cost assumptions are given in **Table** **8**. The MeOH production rate for all strategies is chosen at 1 kg_MeOH_ s^−1^ so that the electricity requirement of the corresponding plant matches with the capacity of today's solar power plants. Specifically, solar MeOH plants with 1 kg_MeOH_ s^−1^ production rate require 1–110 MW solar electricity (depends on the strategy) and the current concentrated solar power and concentrated photovoltaic plants have capacities in the range of 1–400 MW.^226^ Moreover, our choice of production rate is consistent with previous technoeconomic studies on solar MeOH production.^188^, ^221^, ^222^ The direct capital cost of an equipment is calculated through the following equation^220^

(33) $$\mathrm{direct}\;\mathrm{cost}=C_{0}\cdot {\left(\frac{\dot{m}}{{\dot{m}}_{0}}\right)}^{0.67}$$

where ${\dot{m}}_{0}$ and *C*_0_ are the base size (inlet mass flow) and base cost, respectively, as shown in Table 6, and $\dot{m}$ is the mass flow through the unit. The indirect capital cost, including engineering, construction, and contingency, is assumed to be 50% of the direct capital cost. The operating cost includes both utility cost (price given in Table 7) and fixed operating cost. **Figure** **31** summarizes the cost breakdown of the MeOH MSP of the six strategies. For this analysis, the market price of MeOH was taken as US$0.43 kg_MeOH_^−1^.

**Table 8:** Major assumptions for economic analysis

Project economic life	30 years
MeOH production rate	1 kg s^−1^
Solar resource	10 h, 689 W m^−2^
Total capital cost	150% direct cost
Fixed operating cost	10% direct cost
Capital recovery factor	10%

Strategies A and C currently appear to be the most promising options (MSP = US$1.5–2 kg^−1^) with significant reduction in fossil energy consumption over the tradition syngas process. The main cost driver for these two strategies is H_2_ production, the cost of which is largely determined by the solar electricity price and, to a lesser extent, by the electrolyzer cost.^218^ Thus, improvements in solar power technologies (e.g., PVs, concentrating solar power) and electrolysis system efficiency are expected to make these strategies competitive. A further note about strategy A is that the annualized CO_2_ capture and transportation cost is US$0.556 million per year at a CO_2_ flow rate of 0.34 kg_CO2_ s^−1^, which translates into a cost of US$51 MT_CO2_^−1^. This order of magnitude is consistent with the 12‐year United States Government 45Q tax credits of US$35 MT_CO2_^−1^ for CO_2_ captured and put to use for projects started by 2024.^227^ Under this scheme, if the CO_2_ is sequestered, the credit is US$50 MT_CO2_^−1^.

In strategy B, the solar infrastructure (solar collectors and reactors) is the dominant cost component. Improvements in two key process parameters, CO_2_ one‐pass conversion and solar‐to‐chemical efficiency, can lead to cost reductions. Increase in CO_2_ conversions can lead to reduced recycle flow rates, and thus smaller reactors and CO_2_ separation units, but cannot reduce the solar collector cost, which is mainly determined by the solar‐to‐chemical efficiency. In that respect, the discovery of new materials as well as better reactor designs for higher heat recovery can lead to cost decreases.

Strategies D and E have significantly higher MeOH MSP because of the high photoreactor cost, which is affected by the currently low CO_2_ conversion, catalytic activity, and solar‐to‐chemical efficiency. Considerable efforts have to be made in photocatalytic and photoreaction engineering to achieve high reaction rates and maximum solar light utilization.^188^

The major cost driver for strategy F is the solar electricity for PV–electrochemical conversion. Therefore, improving the system Faradaic efficiency and solar electricity generation technology is the key target.

To understand how catalyst deactivation affects the process economics, we calculate the MeOH MSP assuming a 30% deactivation in the CZA catalyst and compare it with previous results based on the fresh CZA catalyst. With the 30% deactivation, the conversion of the MeOH production reaction decreases from 63% to 44% for the CO‐rich syngas feed and from 18% to 14% for the CO_2_‐rich syngas feed. The MeOH yield is now at the high end of the typical industry quoted range for CO‐rich feeds of 14–20 mol% MeOH. The decrease in conversion then leads to 9.4%, 5.5%, 1.5%, and 15% increases in MeOH MSP for the traditional strategy and strategies A, B, and C, respectively. This analysis indicates that the MeOH MSP is relatively insensitive to catalyst deactivation.

Finally, we close with an interesting insight regarding O_2_ separation. The synthesis of MeOH from CO_2_ and water always involves O_2_ generation. In strategies A–D, O_2_ is generated during CO_2_/water electrolysis and splitting, and obtained as pure gas. In strategy F, the two gas products, O_2_ and methane, are generated at different electrodes; thus, pure O_2_ can also be obtained. In strategy E, however, O_2_ and methane are mixed, so extra separation is needed to acquire pure O_2_. In the analysis presented here, we do not consider O_2_ separation—we consider the burning of methane/O_2_ mixture as fuel. However, it is worth exploring separation technologies that would allow selling O_2_ separately since the credit from O_2_ sales can be up to 13% of the total MeOH production cost in some cases.

## 8 Scaling Solar MeOH

The prospect of scaling solar‐assisted chemical processes presents some unique challenges in comparison to traditional process design. In this section, we will review concepts toward scaling particular catalytic materials and photoreactor designs. We will focus primarily on scalability principles for heterogeneous, gas‐phase catalytic systems.

### 8.1 Catalyst Engineering

Catalyst engineering is the first step in developing a heterogeneous catalysis process. Finding a catalytic material that is inexpensive and easy to synthesize, while simultaneously providing adequate activity and selectivity is the first hurdle. Once the catalytic material itself has been developed, determining its optimal macroarchitecture with which it is placed into a photoreactor is the next design step.

#### 8.1.1 Scaling Catalyst Synthesis

Scaling a catalyst synthesis method is crucial for the commercial development of an emerging technology. As we look forward to the development of the technologies described in this review, we acknowledge that many of the current developments have yet to be commercialized, and so they do not have well‐known commercial catalyst synthesis methods. Each technology comes with its own specific requirements for scaling, which impacts the approach for producing catalysts at the kilogram to tonne scale. There are some basic principles that can be applied generally for scaling catalyst production. It is important to understand the key attributes of the catalyst, the synthesis method, and its final form when determining its viability. For more information, the author suggests referring to ref. 228. Herein, a summary of general considerations for scaling catalyst synthesis is presented.

Translating a bench‐scale synthesis into a commercial‐scale production plant is not a trivial process. For this, one must consider how such a process would be achieved at large scales, and whether each process step is practical or commercially available. Many of the processes described within this review are synthesized for bench‐scale, proof‐of‐concept, or small demonstration units. In these instances, the syntheses often 1) use high‐purity reagents, 2) use highly specialized equipment, 3) accept low yields, and 4) ignore the economics. To assess the practicality, one must consider the following:

(1) Potential unsafe conditions for a large‐scale production plant (i.e., temperatures, pressures, toxicity, explosion potential, etc.): If a process step requires high pressures and temperatures, this might be easily achieved in a small lab, but either costly or unsafe at a larger scale.
(2) Toxicity of the reagents: It is possible that some reagents, which can be ordered and shipped to labs in small quantities, cannot be transported to potential production facilities in large quantities.
(3) Ability to adapt to various reagent purities: In practice, lower‐grade reagents may be necessary to reduce costs, and so additional purifications steps may need to be added to the process.
(4) Emissions from the process, and abatement strategies: In a lab setting, emissions are often vented through a fumehood, but in a large‐scale process these emissions may require treatment depending on the local environmental policies.
(5) Economics: The process must also be able to produce a catalyst at a reasonable cost. An approach used by some engineers is that the operating cost of the process must be less than half of the price difference between the product (catalyst) and reagents.
(6) Continuous operating process: The process must be able to operate over an extended period of time, often 24 h a day and 7 days a week without interruption.

The second aspect to consider when scaling a catalyst synthesis is identifying the key characteristic of the catalysts that the product catalyst must have. Before attempting to scale up a synthesis method, the implementer must have a strong understanding of the desired properties of their catalyst, i.e., size, shape, surface, strength, architecture, and active site.

The appropriate synthesis should focus on those that will be applied to this technology. These factors may change depending on the reactor in which the catalyst will operate since there is a significant difference between a thermochemical MeOH reactor and a generic photocatalytic reactor. In each, the preferred catalyst form may be different (i.e., powder, fibers, films, foams, etc.). Approaches for scaling photocatalysts are discussed in subsequent sections.

Scaling solar‐assisted chemical processes poses a unique challenge for chemical engineers. While up‐ and downstream unit operations and auxiliary process components remain more or less unchanged, traditional reactor design and catalyst configurations fall short as in addition to optimized heat and mass transport; the photon interaction with the material must now be added to the list of transport phenomena under consideration. This extends to the reactor internals as well, as it quickly becomes clear that the traditional catalyst architecture of a pressed pellet becomes ineffectual in providing adequate light access and contact with the active sites of the photocatalytic material.

#### 8.1.2 Catalyst Architectures and Manufacture

Once synthesized and validated as catalysts in powder form on the lab scale, the catalyst must be configured in a macroscale architecture in a way that provides efficient heat and mass transport, while allowing light to access the catalytic sites. Furthermore, these architectures must be economical and easy to manufacture. This section will review several catalyst architectures, other than the conventional pellet architecture, that are potentially beneficial for light‐assisted chemical processes found in the literature, including ceramic foams, monoliths, and porous films.

*Foams and Honeycomb‐Type Monoliths*: Foam and honeycomb are types of monoliths that are 3D structures that are distinct based on their differing pore characteristics. They both offer a high ratio of active surface area to reactor volume, which is a desirable characteristic of a catalytic support. Foams and honeycomb‐type monoliths are a prospective material for supporting photocatalysts as they provide the necessary porosity for light penetration and utilization.

While honeycomb monoliths are characterized by their axial straight channels, foam structures have a random arrangement. Catalysts can be deposited onto both substrates, but the random structure of the foams is more suitable for efficient light penetration through the interconnected channels. A foam is a reticulated structure, which exhibits high porosity (often greater than 90%), and in the case of a metal foam, it exhibits other favorable thermal and mechanical properties such as high thermal conductivity, light weight, almost reversible quasi‐elastic deformation, high stiffness, and high energy absorption capacity. However, a ceramic foam exhibits characteristics of high thermal stability and high resistance to corrosion and wear.^229^, ^230^, ^231^, ^232^ The high porosity of the foams ensures low pressure drops, improved mixing, and heat transfer in the reaction system.^231^ Nowadays foams of different materials are available commercially (SiO_2_, Al_2_O_3_, Cu, Ni, Ag, Al, etc.) and according to the Market Research Report published by Grand View Research, the global metal foam demand in 2016 was 1.6 kilotonnes, and the market size of metal foams was valued at US$82 million.^233^ Lately, with the advent of 3D printing technologies, 3D printing metal foam configurations is becoming more economical, opening a new pathway to commercial‐scale foam support systems to maximize surface reactivity.^234^

**Figure 32:**
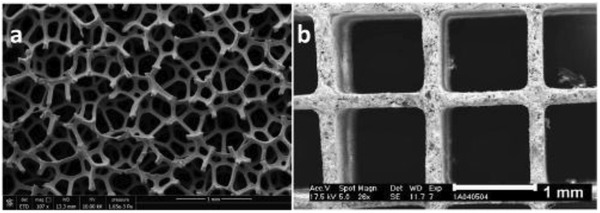
a) Representative image of copper foam (scanning electron microscopy image) and b) honeycomb monolith. Reproduced with permission.^240^ Copyright 2005, Elsevier.

Furthermore, the synthesis of foams in the lab is relatively easy. Foams can be prepared using blowing agents and stabilization particles mixed into the selected metal or molten alloy; the blowing agent decomposes by effect of heat and releases gas that drives the foaming process.^229^, ^235^ On the other hand, a polymeric template can also be employed for the manufacturing of foams, and two different routes can be followed: in the first one (replication process), the interconnected open space of the template is filled with a ceramic material, and after its solidification, the polymeric template is melted/burned out and the resulting structure serves as mold to be filled with the molten metal. Once solidified, the mold is removed and the foam replicates the initial polymeric structure.^236^ In contrast, the polymeric template can be coated with a metallic film (commonly by chemical vapor deposition) followed by a thermal treatment for the calcination of the polymer leading to the final metal foam.^237^ Described procedures represent the classic methods of manufacturing of foams, but nowadays new routes have been developed that enhance control over the pore structure and size and offer the possibility to incorporate different materials in the foam structure, e.g., dealloying and combustion synthesis.^238^, ^239^ This opens the possibility of exploring different foam compositions to enhance the reaction performance. In addition to the composition of the foam, it is also classified by its pore density. The geometric parameter commonly employed for pore density is the number of pores per linear inch. **Figure** **32** depicts a typical perspective of Cu foam and cordierite honeycomb monolith imaged by microscopy.

It is known that the low porosity of solid particles in a packed bed reactor (30–60%) can lead to hot spots within the catalyst bed,^241^ inducing catalyst deactivation and nonuniform heat transfer. In contrast, metallic foams offer a larger surface with homogeneous thermal conductivity. Additionally, the low porosity of a packed bed can contribute to a critical pressure drop across the bed at higher gas flows, thereby limiting the throughput of the reactor system.

Due to the relative novelty of metallic foams as catalytic supports, appropriate models for pressure drop calculations are limited. Edouard et al.^241^ have shown that a modified form of the Ergun equation can be used for pressure drop estimation in SiC foams decorated with SiC nanofibers. This study found that the model predicted that the pressure drop is considerably reduced using a foam structure compared to solid particles. For example, at a gas velocity of 3 m s^−1^, a pressure drop of 1 × 10^4^ Pa m^−1^ was measured for the foam, while in the case of solid particles, the measurement was 8 × 10^4^ Pa m^−1^. Richardson et al.^242^ indicated a factor of 10 between the pressure drop recorded for foams and solid particles in fixed beds.

In the literature, reports can be found of metallic foams as catalyst supports for transformation of CO_2_; however, their use is still limited. One of the reasons for this is that random interconnections in foam structures pose coating challenges, which at present is an area of ongoing research. At present, the best reports published involving transformations of CO_2_ using metallic foams as catalytic supports are the following: use of a Ni–Al_2_O_3_/Ni foam for hydrogenation of CO_2_ to methane,^243^ performance of Ni, Cu, CuZn, and FeCrAl foams supporting a Cu/Zn/Al/Zr catalyst in a MeOH steam microreformer to produce H_2_,^244^ and deposition of a commercial CZA catalyst for MeOH synthesis over Cu open‐cell foams.^245^ In the first example, the thickness of the catalytic film was ≈2 µm and the reaction was performed at 320 °C and 0.1 MPa. A dramatic reduction of hot spots was observed due to enhanced mass transfer. With regard to processes involving MeOH, the work presented by Yu et al. demonstrates the effect of foam composition on both MeOH conversion and selectivity to H_2_. They found that foams with some Cu content exhibited the highest conversions of MeOH, as well as the highest production of H_2_.^244^ Finally, the production of MeOH from syngas catalyzed by a Cu foam coated with commercial CZA catalyst was performed at 5 MPa and at 227 °C and it was found that the performance of the Cu structure was similar to the original CZA powder.^245^ In this case, the authors reported that it was necessary to optimize the coating process to maximize the advantages of the supported catalyst on Cu structures.

Transformation of CO_2_ into valuable chemicals has also been achieved using honeycomb monoliths as catalytic supports, providing an interesting alternative that offers even lower pressure drops than that observed with foams, albeit with less efficient mass transfer.^246^ Nevertheless, honeycomb monoliths have been proven to enhance the selectivity of two‐ and three‐phase reactions by providing a short contact time and relatively small radial diffusion path.^247^, ^248^ It has also been demonstrated that the use of this type of structure considerably reduces the scaling‐up process complexity,^249^ since once the behavior of one capillary is understood, the others will perform similarly, given an even distribution at the inlet. In conventional use, the best‐known example of honeycomb structure use is the catalytic converter in automobiles. However, for the purposes of this review, the work of Liu et al.^250^ is of interest as it describes the design and construction of a bench‐scale MeOH autothermal reformer for H_2_ production in which a mixture of ZnO–Cr_2_O_3_/CeO_2_–ZrO_2_ mixed oxides was used for coating a honeycomb monolith of 400 cells per square inch (6.2 × 10^5^ cells m^−2^). This system achieved almost 100% conversion and an observed production rate of about 160 N m^3^ h^−1^ of reformate per liter of MeOH fed to the system with a product composition of 24:2:21:53 mol% N_2_:CO:CO_2_:H_2_. The aforementioned advantages of honeycomb monoliths have also motivated their assessment in photocatalytic reactions. In this regard, a ceramic honeycomb structure was also used with Cu–TiO_2_ for the photocatalytic reduction of CO_2_ with water vapor;^251^ efficient light distribution in this case was ensured by inserting optical fibers inside the 177 channels. H_2_ and MeOH were found to be the main products at rates of µmol g_cat_^−1^ h^−1^ under UVA radiation.

While previous work has shown that the use of structured catalytic supports is a feasible option in CO_2_ reduction processes, further research still needs to be conducted to develop more efficient catalytic systems. Particularly for solar MeOH production, ceramic supports may be favored for improved deposition of the catalytic layer due to the larger specific surface area and high chemical stability, while metallic foams may be favored for their excellent thermal conductivity that can extend the lifetime of the catalyst by considerable reduction of hot spots otherwise present in catalytic pellets. Both classes of foams are expected to enhance the production of MeOH, maintain a low pressure drop, and improve the overall safety of the process.

*Photocatalytic Films*: The idea of a photocatalytic “panel” seems natural as this form factor has found great success in the world of PVs. Porous films allow for a high surface area, low material waste solution for developing photocatalytic reactors. The tunability of the pore size in the film allows for direct control over mass transfer and optical density of the material, allowing for a customizable configuration that can be adapted to varying applications.

Sordello et al.^252^, ^253^ conducted aqueous‐phase photocatalytic experiments in which they took advantage of the slow photon effect^254^ experienced by photons traveling through inverse opal (IO) structures, which has been shown to improve light absorption and therefore photocatalytic activity. This study found that at wavelengths where the slow photon effect was predicted to occur, the surface normalized photocatalytic rate (µmol m^−2^ min^−1^) was improved over nonporous catalytic material. There are several other examples of similar systems that can be found in refs. 255, 256, 257.

While IO structures representing films with a highly ordered porosity have proven advantageous due to improved photon dynamics, films with disordered porosity have also been shown to improve the activity of photocatalyst powders.^253^ In a study by Sordello and Minero,^253^ disordered porous films outperformed both powder and IO catalysts at wavelengths where the slow photon effect was not expected. This seems more practical as the catalyst is useful in a wider range of incident light energies.

In addition, the manufacturing schemes available for producing porous photocatalytic films are varied and customizable to any required shape, size, and pore size distribution. Lithography, inkjet or 3D printing, conventional bar, blade, or dip coating techniques are low‐cost, easily procurable, and scalable methods by which these reactors panels can be manufactured.

Chen et al.^258^ demonstrated the viability of 3D printing a hierarchically structured porous film for CO_2_ photoreduction using a TiO_2_ catalyst ink, and found that the architecture improved methane and CO evolution by up to sixfold, mimicking a leaf's macro‐ and mesoporous architectures to achieve a high surface area and optimized mass transport.

Care should be taken, however, to monitor and characterize any contaminants that may be present in the final structure due to processing additives or porogenic template materials used to produce these macroporous film architectures. Such contaminants can include organics that can block active sites on the catalyst surface, or preferentially induce the growth of specific crystal phases of faces that may not be advantageous to the target catalytic reaction. Confirmation of observed catalytic rates using kinetic isotope tracers is always recommended in the validation of a new catalytic architecture.

As mentioned, using IOs to improve light access to catalytic sites has the added effect of potentially improving photocatalytic activity through the “slow photon” effect. To expand on this concept, in a photonic crystal structure, such as an IO, the periodic variation in refractive index experienced by a photon propagating through the periodic structure (i.e., air in the pore, followed by the material of the matrix, repeatedly) gives rise to a “photonic band structure,” in an analogous way to the electronic band structure that is formed as an electron propagates through a periodic crystal structure. This photonic band structure gives rise to a photonic bandgap, which prohibits photons with an energy corresponding to the photonic bandgap from propagating through the crystal. Photons with energies that represent the edges of the photonic bandgap experience a reduced group velocity, and are termed “slow photons.”^259^

Numerous publications have pointed out the improvement in photocatalytic activity as a result of improved photon absorption by the catalyst due to their reduced group velocity. This change in behavior as a result of only a change in structure (i.e., the same catalyst in an IO configuration) indicates the importance of careful consideration in catalyst and reactor design for solar‐assisted processes.

While no examples of this exist for the direct production of MeOH from CO_2_, Jiao et al. investigated a CeO_2_ catalyst supported on a TiO_2_ IO for the reduction of CO_2_ and water to CO and H_2_, i.e., syngas, which can be used to synthesize MeOH.^260^ They observed a synergistic effect at the heterojunction between the TiO_2_ IO and the CeO_2_ nanoparticles, in which the “slow light” produced in the IO improved the absorption efficiency of solar irradiation and facilitated the spatial separation of photogenerated electron–hole pairs, leading to an increased photocatalytic activity. They also noted that the “3D ordered macroporous” structure of the IO improved reactant diffusion to the catalytic sites, which also is speculated to have contributed to the increase in activity.

### 8.2 Photoreactor Design and Engineering

The catalytic architectures described in the previous section need to be integrated into a photoreactor, which confines the reaction zone and enables good contact between the photocatalyst, the gas, and the incident photons.

#### 8.2.1 Transport Phenomena Design Considerations and Criteria

Photoreactor design and scale‐up is not straightforward as trade‐offs often arise between heat, mass, and photon transfer when one of the three parameters is optimized without considering the others. Radiation modeling coupled with kinetic, heat, and mass transfer modeling is required for optimization of the reactor and to overcome design challenges. Modeling requires knowledge of the intrinsic kinetic parameters, heat and mass transfer properties of the catalyst architecture in the reactor volume (including radiative heat transfer characteristics), optical properties, and light utilization of the catalyst. Considering the scale dependency of mass and photon transfer, bench‐scale and larger prototype photoreactors need to be designed to screen different catalyst architectures/reactor internals (baffles, fins, ribs, etc.) for model validation and optimization.

*Heat and Mass transfer Considerations*: Mass transfer in a photoreactor needs to be improved through maximizing catalyst contact and mixing with the reactant. Fluidized systems are one of the most investigated due to vigorous mixing of solid catalyst in gas–liquid phase. However, catalyst separation is difficult and overall operation and maintenance costs are high.^261^ To utilize current industrial infrastructure and concentrated sources of CO_2_, gas‐phase fixed bed heterogeneous photocatalysis is a promising scheme. For fixed bed catalysts, catalyst suspension and mixing can be improved through supporting thin film coatings on a larger particle or packing material such as glass beads or other transparent supports. However, such supports tend to be poor heat conductors, which can lead to the formation of local hot spots. The formation of such hot spots can be a desirable effect if the incident radiation is to be converted into heat to drive photothermal catalysis (see Section 4.1.1). However, traditional semiconductor‐based photocatalysts such as TiO_2_ exhibit reduced activity and efficiency at elevated temperature. Hence, efficient heat transport away from the photocatalyst must be enabled in such systems. Structured packing offers several heat and mass transfer advantages over randomly packed beds.

Gascon et al. have thoroughly discussed concepts behind “structuring” at the particle and reactor level to enhance heat and mass transfer, decoupling hydrodynamics, transport phenomena, and reaction kinetics.^262^ Their work is targeted at traditional thermal heterogeneous catalysis; however, these considerations are equally valid for photocatalysts. Briefly, for the entire catalyst particle to be utilized, its diffusion length needs to be minimized or the effective diffusivity into the particle needs to be improved by controlling the range of pore sizes available for gas to diffuse into the particle. Structured packings like monoliths, cross‐flow structures, wire‐knit packing, foams, etc. have several advantages over randomly packed beds, even without optimization. Structured packings have lower but more efficient catalyst loading, allowing better access of the reactants to catalyst active sites. In addition for foams, high flow rates result in turbulence due to its continuous nature and high porosity, which is good for gas mixing and contact between reactants and catalyst. In terms of heat transfer, packed or coated channels offer much better interparticle contact than a randomly packed bed, which leads to better heat conduction, provided a conductive packing material and appropriate wall thickness is selected. For even better heat conduction, cross‐flow structures can be designed to force radial mixing through diagonal flow channels in addition to axial mixing. Such concepts for intensification of thermal catalysis can be applied to photocatalysis.

*Photon Transfer Considerations and Criteria*: Regarding photon transfer for irradiated systems, the position, size, and distance of the light source(s) relative to the photoreactor as well as the type of light source and intensity will affect the final geometry and design of the photoreactor. As light irradiation decreases inversely with the square of the distance from the source of light, it is desirable to minimize the distance between the catalyst surface and light source. However, the shorter the distance, the less uniform the distribution of light over the entire catalyst surface.^261^ For systems reliant on concentrated solar radiation, it is important to completely illuminate the packing surfaces/catalyst internally, through pores or channels, using optical fibers or waveguides, or externally through an optical window or transparent tube positioned along the focal length/point of a solar concentrator. Optimized coating thickness and geometry, dimensions, and spacing of the coated substrate(s) need to be determined for the specific catalyst coating and support system. Voidage and thickness of the coated support need to be optimized based on photon penetration depth and intensity.

In addition to determining the extent of mixing and thermal distribution in the reactor,^263^ comparison of light utilization between photoreactors systems is important for rate comparisons between different systems. Two efficiency factors, the quantum yield (QY, similar to internal quantum efficiency) and the photochemical thermodynamic efficiency factor (PTEF) (Equations (34) and (35)) can be determined by performing an energy balance on the system and quantifying the photons absorbed by the catalyst using a radiation model^264^

(34) $$\mathrm{QY}=\frac{\mathrm{rate}\;\mathrm{of}\;\mathrm{product}\;\mathrm{molecules}\;\mathrm{produced}}{\mathrm{rate}\;\mathrm{of}\;\mathrm{photons}\;\mathrm{absorbed}}$$

(35) $$\mathrm{PTEF}=\frac{\mathrm{rate}\;\mathrm{of}\;\mathrm{irradiated}\;\mathrm{energy}\;\mathrm{used}\;\mathrm{to}\;\mathrm{produce}\;\mathrm{product}\;\mathrm{molecules}}{\mathrm{rate}\;\mathrm{of}\;\mathrm{irradiated}\;\mathrm{energy}\;\mathrm{absorbed}}$$

The PTEF is not commonly reported; however, it is important because it provides information about the energy utilization of the catalyst given the total photon energy from irradiation, under near‐isothermal conditions. By conducting an energy balance on the system, the energy absorbed by the catalyst can be calculated from the total irradiated energy and the unused energy dissipated as heat, which can be quantified by determining the temperature increase of the system. Several studies still report photocatalytic rates and rate constants alone or external quantum efficiency values, which considers the rate as a function of the incident photon flux on the reactor window. For a valid comparison of rates between photoreactor systems, the above‐mentioned QY definition needs to be adopted as there can be significant photon loss through surface reflection and scattering.^264^

Spectral distribution of the light source is an important consideration for designing efficient photoreactors, as most photocatalysts are typically only active over a narrow spectral wavelength range. TiO_2_, which is commonly used for wastewater and air treatment,^265^, ^266^, ^267^ requires a UV light source as it is a wide‐bandgap photocatalyst. However, CO_2_ reduction catalysts like In_2_O_3−_ *_x_*(OH)*_y_* can utilize solar irradiation as they are photocatalytically active at the blue end of the visible spectrum. Therefore, depending on the catalyst used, solar radiation can be used directly or indirectly, the latter in the form of solar electricity to power artificial light sources (e.g., high‐intensity light‐emitting diodes (LEDs)) with a narrower spectral distribution.

#### 8.2.2 Thermochemical and Photochemical Reactor Design

The following section reviews state‐of‐the‐art photoreactor design concepts for both thermochemical and photochemical routes of MeOH production, highlighting potential areas for combining design concepts.

*Process Design for Thermochemical Reactors*: Solar reactors for highly concentrating solar schemes usually feature cavity‐receiver configurations, for example, in a well‐insulated enclosure with a small aperture to facilitate concentrated solar irradiation. Because of multiple internal reflections, the fraction of the incoming energy absorbed by the cavity greatly exceeds the surface absorptance of the inner walls. As the ratio between the cavity's characteristic length to the aperture diameter increases, the cavity‐receiver approaches a blackbody absorber.^124^ Highly concentrating solar schemes typically combine a primary and a secondary concentration stage. Primary concentration of sunlight involves an array of trough (parabolic or enclosed), dish, or heliostat (dual‐axis tracking) reflectors concentrating light onto a focal point or line, along which the secondary concentrator and receiver are placed. A compact 500 m^2^ heliostat field in Spain can provide a flux of 2500 kW m^−2^ (for at least 50 kW) onto an aperture area less than 16 cm^2^. The system is a scaled‐up demonstration of a 4 kW lab‐scale solar reactor (described below) built to achieve temperatures greater than 1300 K for conversion of CO_2_ and water to syngas.^268^

Several reactor concepts have been investigated for thermochemical redox reaction cycles. These include cylindrical cavity‐receivers,^124^, ^126^, ^139^, ^269^ rotating chemical heat engines such as the CR5 (counter‐rotating‐ring receiver/reactor/recuperator),^221^, ^270^ fluidized bed reactors,^271^, ^272^ and indirectly heated particle reactors, in which the solar heat is transferred into the reactor's interior through a heat transfer fluid.^70^, ^273^ The most common reactor configurations are cylindrical cavity‐receivers filled with metal oxide foams or catalyst particles. This configuration, filled with ZnO particles, has been operated for a period of several days at pilot scale in a 100 kW solar reactor.^274^

TEA and life cycle analyses (LCAs) of high‐temperature solar fuel production indicated that solar fuels can be cost competitive with fossil fuels if the solar‐to‐fuel efficiency is at least 20%.^221^ CO_2_ and water splitting into streams of CO, H_2_, or syngas production and O_2_ is generally considered as the bottleneck step of the conversion of solar heat into fuels or chemicals.^188^, ^220^, ^221^ Efficiencies and cyclic rates in thermochemical water/CO_2_ splitting reactors are limited largely by thermal losses resulting from conductive and radiative heat transfer. Theoretically, solar‐thermochemical CO or H_2_ production has higher efficiencies than CO_2_ photoelectrolysis or PEC water electrolysis, for example. Several authors have performed theoretical thermodynamic analyses of thermochemical CO_2_/water splitting cycles. The maximum efficiency of an idealized CO_2_/water splitting process has been estimated at ≈80%; however, solar‐to‐fuel efficiencies of about 38–54% have been demonstrated to be more realistically achievable if 100% of the sensible heat is recovered.^275^ A 20% solar‐to‐fuel efficiency has been shown to be achievable in the absence of heat recovery, exceeding 30% by recovering some sensible heat of the hot products.^224^ Detailed analyses for several systems have provided similar values in the range from 20% to 30%.^132^, ^224^, ^270^, ^276^ For example, a maximum theoretical efficiency of 29.5% has been calculated for 50% sensible heat recovery.^224^ These values are relatively competitive with the efficiency limits for PEC systems, and higher than existing efficiency records for PEC systems.

Experimentally, the benchmark efficiency of thermochemical CO_2_‐to‐CO redox cycles has been reported at 5.25% using a 4 kW lab‐scale solar reactor filled with reticulated porous ceria foam, which was exposed to concentrated solar radiation of 3000 suns.^126^ No sensible heat recovery was performed, which, if integrated into the process, can increase the efficiency of the redox reaction step. An efficiency of 3% has been reported for a pilot‐scale 100 kW solar CO_2_‐to‐CO reactor.^274^ Likewise, no sensible heat recovery was performed.

A theoretical overall solar‐to‐MeOH process efficiency of 7.1% has been calculated using state‐of‐the‐art chemical processing infrastructure and solar concentrator equipment.^221^ This is by a factor of 3–4 higher as compared to natural photosynthesis. An experimentally determined solar‐to‐fuel efficiency of 1.72% has been determined for the entire production chain from CO_2_ and water to synthetic liquid fuel via cosplitting of CO_2_ and water and subsequent Fischer–Tropsch synthesis.^125^ No sensible heat was recovered in that study; hence, the efficiency is expected to be increased if heat recovery is integrated into the process.

Hence, significant improvements of thermochemical redox fuel production cycles are necessary if economically competitive solar MeOH via thermochemical syngas is to be realized. The areas that require further research and development efforts are summarized as follows: 1) redox‐active materials' discovery and optimization (thermodynamics, kinetics, stability); 2) design of redox material architectures, which enable efficient heat and mass transfer to the active sites; 3) reactor designs, which effectively incorporate solar heat, minimize heat loss, and enable sensible heat recovery; and 4) smart integration of thermochemical redox cycles into fuel and chemical synthesis processes (heat recovery, feedstock sources, intermittency of solar radiation).

*Process Design for Photochemical Reactors*: Low‐concentration solar schemes can potentially offer simpler, less expensive, and more compact solutions than high‐concentration solar schemes. Low‐concentration solar schemes typically comprise transparent tubular reactors mounted on parabolic troughs or concentrators. The most efficient of these concentrators are compound parabolic concentrators (CPCs) that reflect all incident rays, direct and diffuse, uniformly onto a central absorber, while enabling turbulent flow and efficient use of the catalyst.^277^, ^278^ With a single‐stage CPC alone, it is possible to achieve a medium temperature range of 80–250 °C if insulation is used.^279^ Such systems have been shown to be scalable in wastewater treatment, treating more than 1000 L day^−1^ of organic contaminants.^277^ A full‐scale plant in Spain comprising of a 100 m^2^ array of low‐concentration CPCs arranged along flat panels can continuously treat 2000 L of nonbiodegradable chlorinated wastewater per day.^278^

Several patents in this field propose new designs to minimize process requirements and improve conversion. One approach is low‐concentration, solar‐irradiated packed bed photocatalytic reactor panels, sectioned with dividers to force a serpentine flow pathway for improved mixing. This design claims to have an increased irradiated surface area compared to an array of tubular reactors, and avoids high pressure drops associated with increasing photoreactor length to improve conversion.^280^ Another approach to achieve high product flux is a hybrid system for contaminant degradation by combining photo‐chemical and thermochemical catalysts/support such that a catalyst region not using the light may use waste process heat from solar heat. The different combinations of catalysts and supports range from coated structured packing to doped metal oxide aerogels and biopolymeric media are arranged into five classes and discussed in a patent by Tabatabaie‐Raissi et al.^281^

Patents incorporating photocatalysis into residential ventilation and air purification units offer design solutions that can be adapted to fixed sources of CO_2_ emission.^282^, ^283^ Along these lines, Negishi and Sano have reported a novel low‐concentration solar tower reactor design operated as a single‐pass flow system at a pilot scale for gas‐phase degradation of toluene.^284^ The solar tower comprises a central coated tubular reactor enclosed by cylindrical scattering mirrors. The top of the tube is directly irradiated with the remainder of the coated tube receiving reflected light from the scattering mirrors. Two catalytic systems were tested with this setup: TiO_2_‐coated ceramic tubes (seven tubes, 21 cm in length) operated at low pressures and a flow rate of 1.5 L min^−1^ and TiO_2_ on silica gel–packed glass tubes (six tubes, 15 cm in length) operated at high pressures and a flow rate of 10 L min^−1^.^284^ Toluene at a concentration of 5 ppm was continuously flowed over the course of half a year, with the first system converting 27.7% of all toluene introduced into the reactor, while the second converted 36% due to a higher surface area.^284^ The height of the solar tower was optimized by measuring the change in toluene degradation when the number of photocatalyst modules was increased, for a fixed light intensity. The solar tower concept enables emission reduction at source and occupies a minimal footprint through vertical stacking of cylindrical scattering mirror modules.^284^

While CO_2_ reduction into CO or MeOH has not been reported with low‐concentration solar schemes, photochemical water splitting has been demonstrated at the pilot scale using flat‐panel and CPC setups.^285^, ^286^, ^287^

*Design Concepts and Examples for Efficient Photochemical Reactors*: Various reactor designs have been devised to facilitate efficient photochemical reactions. Owen et al. provide a framework and examples for developing novel reactor embodiments such as fins, catalyst supports, and waveguides that are multifunctional.^288^ Catalyst support structures and waveguides can be modified or incorporated as reactor internals such as baffles that transmit light while directing the flow pathway and enhancing mixing, in order to satisfy the heat and mass transfer demands outlined earlier. To increase reflected light utilization, uncoated waveguides can be textured to increase side illumination onto catalytic surfaces.^288^ Suitable materials for reactor supports/internals (e.g., Quartzel, silica fiber wool), waveguides, and the reactor itself (e.g., fluorinated ethylene propylene, aluminum) are recommended, considering the presence of a high‐intensity UV light source and temperature increase in the reactor.^288^

**Figure 33:**
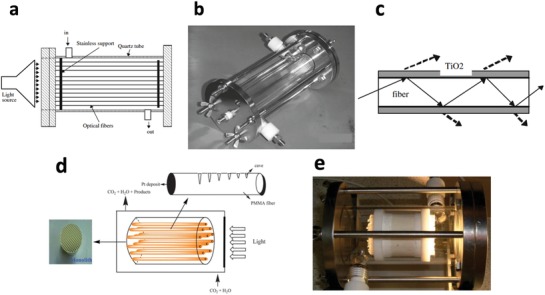
a) Schematic diagram of the fiber optic photoreactor. b) Photograph of the photoreactor. c) Schematic of the fiber optic photoreactor light guide. d) IIMR with light channel inset. e) Operating IIMR. a,c) Reproduced with permission.^187^ Copyright 2009, Springer Nature. b) Reproduced with permission.^217^ Copyright 2010, Springer Nature. d,e) Reproduced with permission.^291^ Copyright 2011, Royal Society of Chemistry.

Wu applied photocatalyst‐coated fiber optic cables for the direct CO_2_‐to‐MeOH conversion approach.^187^ **Figure** **33**a–c depicts the schematic for the coated fiber optic cables and the photoreactor used in their work. Using anatase TiO_2_ and Cu@TiO_2_ composite photocatalysts, he examined the effect of UV irradiation, temperature, and pressure on the activity. His best result was 0.45 µmol_MeOH_ g_cat_^−1^ h^−1^ using 1.2 wt% Cu@TiO_2_. An attempt to improve the light utilization of the fiber optic–coated reactors was carried out by Ren and Valsaraj.^289^ They applied an IO synthesis technique to coat the fiber optic with Cu@TiO_2_ photocatalyst. While they report a lower MeOH production rate of 0.036 µmol_MeOH_ g_cat_^−1^ h^−1^, the authors claim that their photocatalysts improved the quantum efficiency of conventional TiO_2_, which they report as 0.035–0.47%. Wang et al. also compared MeOH production rates between an optical fiber reactor (OFR) and an internally illuminated monolith reactor (IIMR) for NiO/InTaO_4_ catalyst coating at 25 °C and different light intensities.^290^ The internal channels of a ceramic IIMR were coated with catalyst and illuminated by placing waveguides along each channel. Carving the waveguides and coating the tip with aluminum for backward reflection of light was shown to be an effective way of transmitting and scattering most of the light sideways to illuminate the internal channels (Figure 33d,e). The OFR was found to have a higher specific rate of MeOH production than the IIMR, whereas the IIMR had a higher throughput than the OFR. The IIMR showed quantum efficiency of nearly one order of magnitude higher than the OFR.^291^

Both OFRs and IIMRs minimize the distance between the catalyst surface and the light source, while maintaining uniform distribution of light over the entire catalyst surface. To compensate for the exponential drop in light intensity along the length of the optical fiber (uncladded), the fiber diameter can be increased to reduce the number of reflections per unit length of the fiber, while also minimizing volume and thickness of the coating.^292^

**Figure 34:**
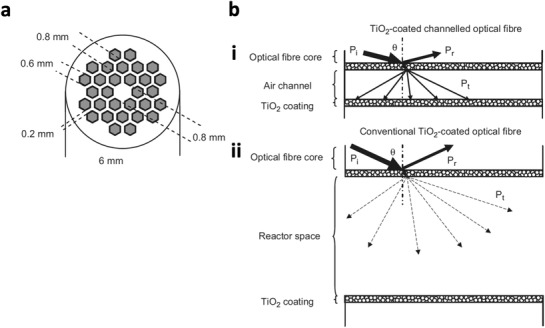
a) Schematic of channeled optical fiber with dimensions. b) Schematic of light pathway in i) TiO_2_‐coated COFR and ii) conventional TiO_2_‐coated OFR. Dashed arrows illustrate scattered light of lower irradiance compared to the light within the optical fiber. Adapted with permission.^293^ Copyright 2010, Elsevier.

For OFRs and IIMRs, the dependency of QY on intensity and angle of incident light is an important limitation in extending utility of these reactor configurations. Denny et al.^293^ have come up with a channeled optical fiber reactor (COFR) comprised of 30 distributed hexagonal air channels with a solid central core (as shown in **Figure** **34**a) to overcome these limitations. The design increases the catalyst surface area capable of absorbing diffuse refracted light (otherwise lost to surroundings) through coating hexagonal channels, while maintaining the optimal catalyst film thickness for light absorption, as described in Figure 34bi,ii. These results open the possibility of coupling COFR design to concentrated solar schemes to increase radiation intensity received by the catalyst without experiencing high efficiency losses in the reactor.^293^

LED‐irradiated photoreactors are becoming increasingly favored over larger UV/halogen lamps as they offer more efficient light generation, flexibility for design, and pulse‐width control to manage temperature in the photoreactor and utilize power more efficiently. Complete modeling and validation of an LED‐based annular photoreactor for toluene degradation has been reported by Khodadadian et al.^294^ The LEDs used in this study provided monochromatic, narrowband emission in the UV spectrum (maximum intensity at 365 nm). Key trends in rates, conversion, and the relationship of toluene degradation to irradiance and reactant composition were established. Operating conditions under which catalyst deactivation occurred and where mass transfer limitations dominated photon transfer were identified by testing a wide range of operating conditions and reactant concentrations.^294^

Recent reviews summarize production rates, product selectivity, and apparent quantum yields for different CO_2_ reduction photocatalysts given the reactants, light source (halogen lamps, UV LED, solar concentrators, etc.), and the operating wavelength range.^178^, ^295^ The wavelength of light can be critical in significantly enhancing photocatalytic rates (by up to 24‐fold) particularly in plasmonic photocatalysts.^296^ Apart from the light source, product formation also has a strong dependence on light intensity. For CO_2_ reduction by TiO_2_ irradiated by UV light, Dilla et al. established a linear increase in methane production rates over a specific range of low light intensities, while determining that methane production rates increased as a square root of light intensity at higher light intensities due to charge recombination dominating over charge transfer reactions.^297^

For UV lamp photocatalytic systems, and most experimental systems that currently use lamps, there are several benefits to switching to LEDs. In terms of efficiency alone, external quantum efficiencies of up to 40% have been achieved in LED‐illuminated reactors compared to that of 15% achieved under lamp illumination.^298^ Moreover, the small size of LEDs can be harnessed to design inexpensive, compact reactors in which innovative design of reactor internals in conjunction with LEDs can minimize the distance between catalyst surface and light source, while improving surface area to volume ratio to enhance mass transfer.

Due to their size, LEDs are also an ideal light source for internal illumination of a closed reactor system. Closed reactor systems illuminated internally can potentially achieve high pressures as they are not constrained by the pressure limitations of transparent materials used for optical windows. Furthermore, they have scale‐up advantages in that they can have a tall design to minimize area footprint, and can make use of stronger materials of construction such as steel. Patents have described ways of incorporating LED silicone molded parts (plates, pipes, tubes) that can be bundled to form lighting inserts, which can be positioned to ensure maximal radiation intensity and uniform surface illumination.^299^

## 9 Incorporating PVs into the Thermochemical MeOH Process

In addition to the development of solar‐thermochemical, photothermal, photochemical, PV–electrochemical, and photoelectrochemical systems, it is also conceivable that light could be harvested to produce electrical energy using PV panels or heat energy using solar concentrators/absorbers for process utility uses. In this way, we can consider the possible “solarization” of thermochemical MeOH synthesis. In order to provide a perspective on the feasibility of using such methods of light harvesting, land area and installation cost estimates have been made based on the electricity and thermal energy input required by traditional and more modern facilities intended for the production of MeOH. These electrical energy estimates, in particular, represent the combined energy required for several key processes, including the operation of compressors, pumps, cooling fans, and other mechanical equipment. As part of an LCA on a fully solar‐powered MeOH synthesis process utilizing CO_2_ and water, a value of 10% was proposed as a reasonable time‐averaged solar‐to‐electrical energy conversion efficiency for PV cells.^300^ This value matches closely with reported values (8–9%) for utility‐scale thin‐film silicon PV panels.^91^ Installation costs for these PV systems are based on an estimate of US$4.80 W^−1^.^91^ In terms of sunlight, a mean daily horizontal solar irradiation of 4.32 kWh m^−2^ day^−1^ was suggested, assuming that the proposed facilities would be located in a near‐equatorial region, such as within 5° of the equator.^67^ However, presupposing a high degree of annual incident solar irradiation means that area requirements and installation costs could be considerably greater for regions receiving less direct sunlight. For installations in less‐equatorial locations, other sources of renewable electrical energy, such and wind and tidal, would likely be more competitive in terms of both space requirements and cost, and would thus be preferred over PVs; however, such considerations are beyond the scope of this review.

Before proceeding further, it should be noted that studies of TCST processes sometimes produce estimated energy consumption values that differ by as much as an order of magnitude.^124^ Based on values reported by the company Lurgi, a commercial‐scale MeOH production route should be expected to consume ≈20 kWh of electrical energy per MT_MeOH_ produced. Based on this calculation, producing 82 700 MT_MeOH_ annually (226 MTPD_MeOH_, assuming continual operation) would require 0.01 km^2^ of land for PV panels, corresponding to a square with side lengths of 100 m, at an installation cost of US$1 million. When researchers attempted to perform their own energy estimates based on the proposed system, however, a significantly greater electrical energy requirement of 213 kWh MT_MeOH_^−1^ was calculated. Based on this larger energy requirement (factor of 10), calculations predicted that 0.1 km^2^ of land would be needed to accommodate PV panels, corresponding to a square with side lengths of 316 m, and costing an additional US$10 million for installation. As can clearly be seen, this constitutes an approximately tenfold difference in expected electrical energy consumption, land requirements, and cost, thereby illustrating the considerable variability that can occur when attempting to estimate requirements based on data from disparate sources. This variability should be kept in mind when considering the following estimates for MeOH synthesis facilities, which may deviate appreciably from the actual values for an operational facility.

### 9.1 PV Electrification of a Coupled Biomass Processing and MeOH Synthesis Facility

Given the assumptions and estimates mentioned above, similar calculations can also be performed for a more complex facility intended for the production of MeOH from biomass. This facility was designed for both converting biomass into syngas and then synthesis, with much of the required process heat being derived from the combustion of additional biomass. In the case of a syngas‐to‐MeOH plant designed to produce 520 MTPD_MeOH_ as part of a larger complex (about one‐tenth the size of a “Mega” MeOH facility), electrical power consumption is expected to be on the order of 13 850 kW.^301^ Thus, the same plant scaled to 82 700 MT_MeOH_ annually (226 MTPD_MeOH_) would require a PV area of 0.33 km^2^ (a square with side length of 580 m), corresponding to an installation cost of US$29 million and an electrical energy consumption for the coupled system of 640 kWh MT_MeOH_^−1^. It should be noted that this particular MeOH synthesis plant draws some of its required heat energy from other components of the complex, thereby reducing its input energy requirements and eliminating the need for electrification of heating.

### 9.2 PV Electrification of a CO_2_‐Rich Syngas MeOH Synthesis Facility

From an emission perspective, using solar energy from PV panels for the production of MeOH via water electrolysis and captured CO_2_ could help to further reduce the net CO_2_ emissions associated with this technology. For comparison, the requirements for producing an amount of MeOH equal to that in the preceding example (226 MTPD_MeOH_) were calculated. In the current example, a simplified summary of electrical and heating energy costs was not provided for the overall process; instead, different variations were presented depending on the chosen source of CO_2_. Energy costs for water electrolysis to produce H_2_ (10.3 kWh kg_MeOH_^−1^) were significantly greater than those for concentrating CO_2_ (1.68–2.75 kWh kg_MeOH_^−1^) and MeOH synthesis from the resulting syngas (1.27 kWh kg_MeOH_^−1^).^301^ If CO_2_ were isolated from the atmosphere, where it occurs at a concentration of ≈409 ppm (as of September 2018),^302^ providing the sum total of electrical and thermal energy would require 1.44 km^2^ of land (square of side length of 1200 m) and US$124 million. Sourcing the requisite CO_2_ from a cement plant (from which CO_2_ can be obtained at a concentration of 25 mol%)^301^ results in somewhat more favorable requirements, necessitating 0.88 km^2^ (a square with side length of 940 m) and US$76 million; however, this approach also presents the possibility of using waste heat from the plant itself to alleviate thermal requirements, which could also improve its viability.^129^ Finally, sourcing richer (49 mol%)^301^ CO_2_ streams from a biogas plant would further reduce the area and capital cost requirements slightly to 0.56 km^2^ and US$48 million. Energy consumption for these various methods ranged from 6390 to 7460 kWh MT_MeOH_^−1^. However, this accounts only for the isolation of CO_2_; both the synthesis of MeOH and the water electrolysis to produce H_2_ must still be considered. On average, 12% of the required energy is used for sourcing CO_2_, 78% for water electrolysis, and 10% for producing MeOH from syngas.

### 9.3 PV Electrification of a Solar‐Thermochemical MeOH Synthesis Facility

A final example is taken from the LCA of a proposed system employing solar‐thermochemical heating to catalyze the conversion of CO_2_ and water into syngas, and then using the product gases to perform MeOH synthesis according to traditional methods. In order to sustain the production of 82 700 MT of MeOH per year (226 MTPD_MeOH_), the proposed system would require a process electricity input of 28 294 kW.^300^ It should be emphasized that this proposed system is based on an assembly of nearly 18 000 individual reactors, each of which has a power draw upward of 1 kW,^270^ resulting in high process electricity consumption; however, it is likely that this consumption could be significantly reduced by consolidating or scaling up these reactors, or via further process optimization. Given this 28 294 kW energy demand and the estimated efficiency of the PV cells, a land area of ≈1.57 km^2^ would be required, corresponding to a square region with sides roughly 1250 m long. Incorporating such a large array of panels would require a prohibitively large initial investment on the order of US$136 million for installation alone, constituting an additional 22% on top of the originally proposed capital costs.^300^ Although this would eliminate the need to purchase electricity from a utility company, the solar electricity generated using this method would be quite costly at US$0.55 kWh^−1^, which is much more expensive than the estimated utility price of US$0.06 kWh^−1^. It is plausible that purchasing such a bulk quantity of equipment from an industrial vendor would afford some price break, and the cost of the bare modules themselves from the factory gate is roughly US$0.88 W^−1^ (not including any associated battery storage),^91^ and would reduce the cost of electrification to just US$25 million, constituting an additional 4% contribution to capital costs. Overall, incorporating solar PV systems into an existing technology represents a significant investment in terms of both land area and capital funds, and could potentially compromise the economic viability of such a facility.

While it is theoretically possible to use the electrical energy generated by such PV installations to provide highly efficient high‐grade heating for the aforementioned solar‐thermochemical process, doing so would require an additional 148 521 kW of energy (this excludes the energy used in the solar‐thermochemical reduction of CO_2_). Unlike the solar‐thermochemical syngas production (described in Section 4.1.4), this system employed some steps that were particularly energy‐intensive, including amine scrubbing, to achieve optimal CO:CO_2_:H_2_ concentrations for MeOH synthesis (in this case 34:5:61 mol%), and distillation of the MeOH/water product to achieve high purity. Even assuming 100% electrical‐to‐heat conversion, this would necessitate upgrading the installation to include 9.82 km^2^ of PV panels (a square with side length of 3130 m) and the contribution US$842 million in capital costs (the additional expense more than doubling the initial cost of US$614 million). The overall electricity consumption for such a process would be on the order of 18 800 kWh MT_MeOH_^−1^. These dramatically large requirements in terms of both area and cost make such conversions far from trivial, and are undesirable for a facility that already projects negative lifetime profits.^300^ Fortunately, alternative approaches involving the concentration and focusing of sunlight to directly provide heat to the system could offer significantly higher solar‐to‐heat energy conversion and prove substantially more viable, overall.

### 9.4 Solar‐Thermochemical and Solar‐Thermal Energy for Utility Heat

As discussed previously, focused solar irradiation can be used to generate high temperatures and drive useful catalytic chemical reactions. Using appropriate focusing mirrors and optics, solar flux concentrations of up to 3000 suns and temperatures of as high as 1773 K can be achieved,^124^, ^126^ given a direct normal solar radiation intensity of 1 kW m^−2^. However, there is no reason that similar technologies could not be used to drive both traditional thermochemical reactions and auxiliary processes requiring process heat. The solar‐to‐heat efficiencies observed for solar concentrator systems are typically well above the analogous solar‐to‐electricity efficiencies attained by PV devices. While values for the former range from ≈20% to 70%, depending on operating conditions and the technology employed,^124^, ^303^, ^304^ those of the latter have a theoretical maximum of slightly above 30%, but utility‐scale installations typically offer performances closer to 9% (time‐averaged).^305^ Using solar gas turbines, sometimes referred to as “power towers,” net solar‐to‐electricity efficiencies as high as 20% could be realized,^124^ making such technologies competitive with, or even superior to, commercially available time‐averaged PV devices.^305^ Meeting input heat requirements using solar‐to‐heat conversion, however, seems to offer greater potential than does employing PVs for the generation of process heat.

### 9.5 Practicality Assessment of PV Electrification of Traditional and Solar MeOH Processes

**Figure 35:**
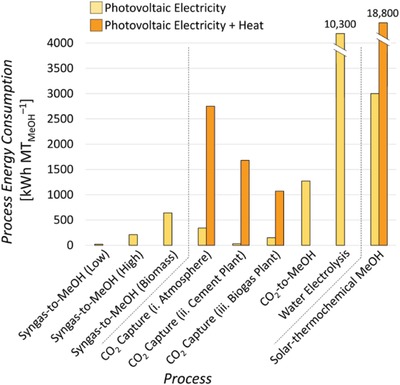
Process energy consumed per MT_MeOH_ produced by various plant designs.

As can be seen in **Table** **9** and **Figure** **35**, significant variation in the estimated costs of electrifying MeOH synthesis processes can occur; however, typical ranges of values can be generally established. Area requirements for providing process heat and electricity using PV electricity ranged from 0.01 km^2^, for a small and simple syngas‐to‐MeOH facility, to as much as 9.82 km^2^, for a facility capable of producing MeOH directly from feedstocks of CO_2_ and water. PV installation costs varied over a similar relative range, from as little as US$1 million for the least expensive installations to as much as three orders of magnitude higher for the most expensive. Clearly, the activation and conversion of simple feedstocks (CO_2_ and water) into value‐added products is a much more involved process, naturally requiring a greater degree of energy input. Some part of this discrepancy can be attributed to the fact that well‐established thermochemical strategies have benefited from decades of optimization and streamlining, whereas new strategies incorporating light are only beginning to emerge as viable technologies. This is also an important consideration to keep in mind when evaluating the viability of new technologies, as there could be ample room for further optimization as these technologies mature.

**Table 9:** Energy, land area, and capital costs associated with solarization of each of the process plants

Section	Facility	Electricity/electricity + heat	Energy consumption [kWh MT_MeOH_^−1^]	Area required [km^2^]	Square side length [km]	Photovoltaic installation cost [US$ million]
9	Syngas‐to‐MeOH (low estimate)	Electricity	20	0.01	0.1	1
	Syngas‐to‐MeOH (high estimate)	Electricity	210	0.11	0.33	10
7	Traditional strategy	Electricity	1111a)	0.58	0.76	–
9.1	Syngas‐to‐MeOH (biomass)	Electricity	640	0.33	0.58	29
7	Biomass strategy	Electricity	1388	0.73	0.85	–
9.2	CO_2_ capture from…					
	… Atmosphere	Electricity	340	0.18	0.42	16
	(400 ppm CO_2_)	Electricity + heat	2750	1.44	1.2	124
	… Cement plant	Electricity	30	0.01	0.1	1
	(25 mol% CO_2_)	Electricity + heat	1680	0.88	0.94	76
	… Biogas plant	Electricity	150	0.08	0.28	7
	(49 mol% CO_2_)	Electricity + heat	1070	0.56	0.75	48
	CO_2_‐to‐MeOH	Electricity	1270	0.66	0.82	57
	Water electrolysis	Electricity	10 300	5.38	2.32	468
9.3	Solar‐thermochemical H_2_O/CO_2_‐to‐MeOH	Electricity	3000	1.57	1.25	136
		Electricity + heat	18 800	9.82	3.13	842

^a)^ The traditional strategy in the TEA uses electricity for the MeOH synthesis step, which is nonstandard practice.

In terms of both area and installation cost, however, these values represent a substantial, if not prohibitive, obstacle to the electrification of existing systems. In many scenarios, the area and cost requirements for converting the process to run on PV electricity are highly impractical; however, these area requirements can theoretically be offset by installing of PV panels on the roofs of buildings. This finding serves to illustrate the importance of incorporating photocatalysis directly into these processes, thereby increasing the efficiency of light utilization. Given the greater demand for process heat than electricity, as well as their significantly greater efficiencies of solar energy conversion, the implementation of solar‐thermal technologies offers the greatest potential for the solarization of thermochemical processes.

## 10 The Cyclical CO_2_‐to‐MeOH Economy: Downstream Product Market

Utilizing MeOH as a fuel has some limitations. They include that it is more toxic by ingestion than gasoline and the flame is invisible under normal illumination. Olfactory (e.g., methyl *tert*‐butyl ether), flame, and colorizing additives have been proposed to mitigate exposure.^306^ However, the benefit of MeOH is that as a neat fuel it has an octane number near 100 and it releases less heat during combustion making internal combustion engines run cooler and more efficiently, although the volumetric energy density is about half of gasoline.^307^ Alternatively, DME (popularly known as e‐fuel when derived from CO_2_), a dehydration product derived from MeOH, has a high cetane number of 60,^308^ is relatively nontoxic, has only slight narcotic effects up to 75 000 ppmv after 12 min,^309^ is cleaner burning than diesel, is biodegradable, and has a visible yellow flame. DME has the potential of fueling long‐distance diesel trucks far into the future. As such, DME will be considered in this section as a value‐added derivative of MeOH or produced in tandem from intermediate CO_2_‐rich syngas (CO_2_/CO*_x_* ratio of 0–0.5) achievable from solar syngas preparation steps.

### 10.1 MeOH versus DME Synthesis from Syngas Feeds

**Figure 36:**
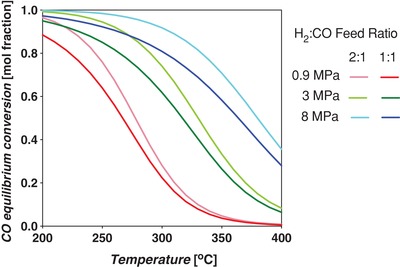
DME equilibrium yield with temperature.

The use of a bifunctional catalyst can facilitate the production of DME from a syngas feed. PODEs are homologues of DME and have also shown promise as a diesel fuel replacement as they are liquids at ambient conditions. As of now, however, the only heterogeneously derivable product is the smallest homologue PODE‐1.^310^ Although DME synthesis is thermodynamically more favorable than MeOH synthesis (see **Figure** **36**), it requires specialized heat managing reactors of the slurry type.^311^ DME not only has superior equilibrium yield as shown for 1:1 and 2:1 H_2_:CO in that figure, but furthermore at moderate pressures. This is due to the heterogeneous DME synthesis reaction (or MeOH dehydration) going through an equimolar second step shown in Equations (36) and (37)

DME from CO‐rich feed (2:1 H_2_:CO feed)

(36) $$\mathrm{2CO}\;+\;{\mathrm{4H}}_{2}⇌\;{\mathrm{2CH}}_{3}{\mathrm{OH}}_{\left(g\right)}⇌\;{\mathrm{CH}}_{3}{\mathrm{OCH}}_{3(g)}\;+\;H_{2}O_{\left(g\right)}$$

DME from CO_2_‐rich feed (3:1 H_2_:CO_2_ feed)

(37) $$\begin{array}{l} {\mathrm{2CO}}_{2}\;+\;{\mathrm{6H}}_{2}⇌\;{\mathrm{2CH}}_{3}{\mathrm{OH}}_{\left(g\right)}\\ \;\;\;\;\;\;\;\;\;+\;{\mathrm{2H}}_{2}O_{\left(g\right)}⇌\;{\mathrm{CH}}_{3}{\mathrm{OCH}}_{3(g)}\;+\;{\mathrm{3H}}_{2}O_{\left(g\right)} \end{array}$$

At 0.9 MPa, 250 °C, and H_2_:CO ratio of 2:1, the CO equilibrium yield to DME is 3400% higher than the CO equilibrium yield to MeOH (76% vs 2.2%).

In a study by Ateka et al.,^148^ a thermodynamic analysis on MeOH versus DME synthesis was conducted to establish which product converted more CO_2_ at various CO_2_:CO*_x_* feed ratios, up to 4 MPa and H_2_:CO*_x_* ratio of 3:1. DME is more favorable in terms of equilibrium yield to oxygenates at lower temperature and pressures. As the CO_2_:CO*_x_* ratio decreases, equilibrium yields improve. At low CO_2_ feed ratios (CO_2_:CO*_x_*) of 0:1 to 0.5:1 at 250 °C and 4 MPa, CO_2_ conversion is higher for MeOH production, and comparable to DME at CO_2_:CO*_x_* ratios of 0.5:1 to 1:1. DME equilibrium oxygenate yield is near 50% versus 30% for MeOH at 250 °C, 3 MPa, CO_2_:CO*_x_* ratio of 0.5:1, the selectivity toward DME (*Ŝ*_DME_) at 90%, and *Ŝ*_MeOH_ (computed herein) of 100%. Conversions of CO_2_ were 5% and 10% for DME and MeOH from that study, respectively. As a result, MeOH synthesis is comparable (Absolute 0.38 mol% CO_2_ in feed to MeOH) to DME (0.28 mol% to DME) at the low to intermediate CO_2_ in the feed. At CO_2_:CO*_x_* ratios greater than 0.5:1, DME is superior (*Ŝ*_DME_ near 80% at 250 °C, 4 MPa, and CO_2_:CO*_x_* ratio of 1:1), but the latter feeds have the disadvantage of high water production and its effect (discussed in Section 4.3.2) on the commercial CZA catalyst. Syngas feeds with CO_2_:CO*_x_* ratios of 0:1 to 0.5:1 are not only accessible with solar syngas preparation methods, but more importantly closer to the optimal feed composition for commercial CZA catalysts; therefore, MeOH is a competitive product with DME, in terms of CO_2_ converted, with minimum investment in RWGS technology. MeOH is also a more flexible feedstock for fine chemical synthesis as mentioned in the introduction.

Water will continue to be an issue in MeOH and DME syntheses using CZA, and as such the intermediate to low CO_2_ in the feed is beneficial. In terms of thermal management, it was found that by increasing the CO_2_:CO*_x_* ratio, the effect was to decrease the heat generated. For example, for CO_2_‐free, CO_2_:CO*_x_* of 0:1 and 0.5:1, the heat decreased by 44% for MeOH synthesis, from −45 to −25 kJ mol^−1^. Cooling remains challenging for DME synthesis, but with MeOH competitive at low CO_2_:CO*_x_* ratios with less produced water overall, it remains a valid reaction pathway for CO_2_ utilization.

## 11 Realizing Olah's MeOH Dream

**Figure 37:**
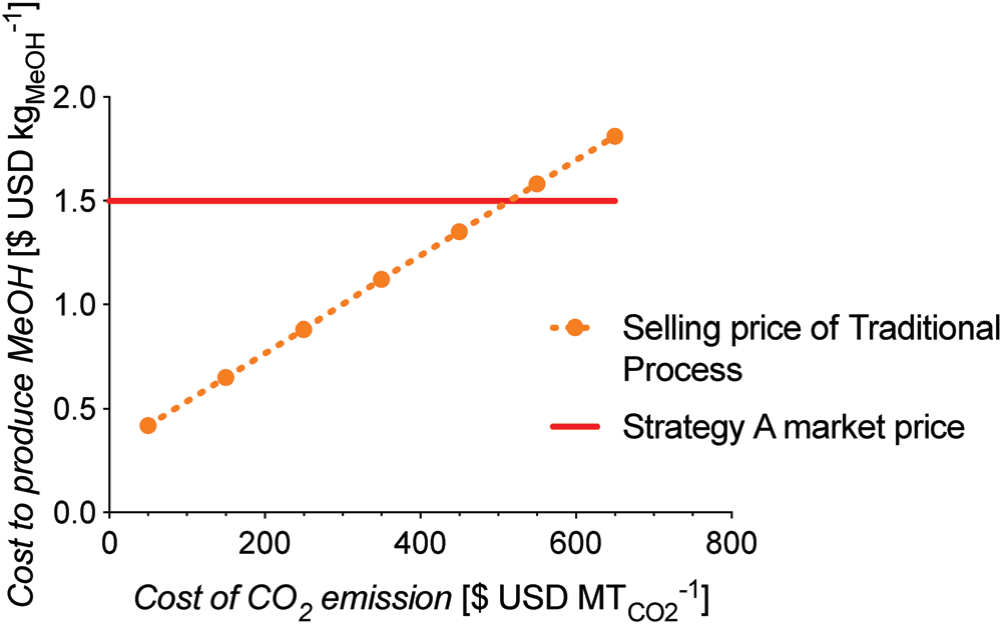
Sensitivity analysis of cost of MeOH with the carbon tax price.

Based on the TEA of various solar‐assisted processes (strategies A–F), strategy A was found to be the most economically viable. The following section will make some conclusions about the viability of this solar MeOH technology in the near‐term international context. Based on our analysis, strategy A made use of PV–electrochemical water electrolysis and solar‐thermal RWGS for syngas generation feeding a thermochemical MeOH synthesis reactor using the commercial CZA catalyst. For strategy A, η^Primary^, defined in Equation (32), is 9% versus 54% for the traditional strategy. The cost of MeOH is also less favorable for the solar strategy A, at US$1.5 kg_MeOH_^−1^ versus US$0.3 kg_MeOH_^−1^ due to the added cost of solar collection, water electrolysis, and CO_2_‐to‐CO RWGS production, and carbon capture (CC). The silver lining for the solar strategy, however, is that less fossil energy is consumed in producing MeOH at 9.9 MJ kg_MeOH_^−1^, down from 42.6 MJ kg_MeOH_^−1^ for the traditional strategy. Thus, the quantity of CO_2_ emissions per kg_MeOH_ produced is reduced by more than fourfold. The potential economic impact of this is if a company emitting additional CO_2_ (by combusting more NG) is charged a carbon tax. A company with a solar‐assisted process could avoid this expense. By taxing carbon emissions, the traditional strategy's production price becomes more expensive due to the extra capital spent in emission taxes. At some carbon tax amount, US$ MT_CO2_^−1^, the solar strategy A becomes competitive with the traditional strategy. To determine the breakeven point, a few parameters need to be estimated. The two most important are the amount of CO_2_ produced during the combustion of NG to produce a MJ of heat and electricity. From one source, these values are 56 and 107 g_CO2_ MJ^−1^ (386 g_CO2_ kWh^−1^), for NG to heat and power, respectively.^312^ Based on the TEA described here, the ratio of energy requirements for the traditional strategy in terms of heat and power is 2.5 (10:4 MW heat:power). Using this ratio, a combined weighted average CO_2_ emission for NG utilization for the traditional strategy of 70.6 g_CO2_ MJ^−1^ is derived. This value combined with the difference of 32.7 MJ kg_MeOH_^−1^ between strategies is an additional 2.33 kg_CO2_ kg_MeOH_^−1^ for the traditional strategy. Correcting the MeOH price of the traditional Strategy by adding CO_2_ emission taxes yields a sensitivity diagram shown in **Figure** **37**.

To give some perspective, the breakeven point occurs at ≈US$500 MT_CO2_^−1^ well above current Canadian federal schemes where it is slated to begin a Can$20 MT_CO2_^−1^ in 2019 and only rise to Can$50 MT_CO2_^−1^ by 2022. This, together with a softened corporate CO_2_ emission responsibility of 20%, is currently an inadequate incentive for prospective solar MeOH via strategy A in Canada.^313^ As mentioned, the US 45Q is a tax credit of US$35 MT_CO2_^−1^ for CO_2_ captured and US$50 MT_CO2_^−1^ for CO_2_ sequestered by 2024,^227^ which is also an insufficient incentive.

Recently, a report supported by the Global CO_2_ Initiative was released that highlighted the breakeven price for a CO_2_‐based thermochemical CCU MeOH production plant coupled to cement manufacturing.^314^ They found with 95% confidence a 4.9‐ to 5.8‐fold greater price than current market price of TCST process. Based on the estimate of the TCST MeOH price herein, this works out to a price of US$1.47–1.74 kg_MeOH_^−1^, equivalent at the low end to the solar‐assisted strategy A of US$1.5 kg_MeOH_^−1^. Some assumptions they made are polymer electrolyte membrane (PEM) water electrolysis (TRL9), membrane CO_2_ capture (TRL4), adiabatic MeOH synthesis section (210/288 °C enter/exit temperature), 7.8 MPa pressure (TRL7), 3:1 CO_2_:H_2_ feed, 22 mol% CO_2_ flue gas, and 2016 base year. Electricity was found to be the main cost contributor, and the renewable quality of it made the process from 30% to 90% less fossil carbon intensive (low to fully decarbonized) than the TCST process. Strategy A was found herein to be 76% less fossil carbon intensive than TCST and this assumes a breakeven price based on a 10% capital recovery factor (calculated based on a 9% rate of return and project lifetime of 30 years), making it comparable to the best carbon reduction scenario of the CO_2_‐based thermochemical process in that report under the best renewable electrification scenario (on‐shore wind energy only). Furthermore, the CO_2_ for strategy A is sourced from a less concentrated coal plant flue gas (13.4 mol% CO_2_) lending further credence to the viability of a solarized process.

### 11.1 Improvements to Strategy A

The S‐E cost assumed in the TEA was US$0.14 kWh^−1^ and F‐E of US$0.06 kWh^−1^, a ratio of 2.3. Similarly, the cost of heat from solar was US$0.07 kWh^−1^ and heat from fossil was US$0.01 kWh^−1^, or a ratio of 7. Solarization does not come into play for CC and MeOH purification, where fossil demand is split 75:25% respectively, and represents 16% of the energy for the process. The most benefit to strategy A will come from increasing the S‐E efficiency, where electricity from solar accounts for 81.6% (36.7 MW power/45 MW total × 100%) of the energy demand from the TEA analysis. About 228 MW of solar, representing 94.2% of the energy input is converted into 36.7 MW electrical, or an S‐E efficiency of 16%. As H_2_ production represents 80% of the MeOH production cost for strategy A, if the S‐E price falls to US$0.11:0.087:0.06 kWh^−1^, the cost of MeOH would come down to US$1.24:1.04:0.8 kg_MeOH_^−1^. With the highly optimistic S‐E cost of US$0.06 kWh^−1^, which is on par with the F‐E cost of US$0.06 kWh^−1^ the solar MeOH plant would be competitive at ≈US$210 MT_CO2_^−1^. Of course, increasing the production rate, by increasing the yield of CO_2_‐to‐MeOH by developing more active low‐temperature and water‐stable catalysts would negate the RWGS step, but this step only accounts for 4% of the energy demand. Solarizing CC and MeOH purification would also help, decreasing energy demand a further 16%. However, the main cost driver remains the H_2_ cost.

### 11.2 Combining Solar MeOH Synthesis with Other Processes

A more immediate application, given the less than ideal global carbon economics for solar MeOH, could be to couple production with heavy industries such as steel production. Roughly 50% of emissions from large chemical processes such as steel or cement production are a direct product of the combustion of fossil fuels at the plant site to generate heat for the process.^315^ CO_2_ is also a product in the reduction of iron ore for steel production, and calcium carbonate reduction for cement manufacture as shown in Equations (38) and (39)

(38) $${\mathrm{Fe}}_{3}O_{4}\;+\;\mathrm{2C}\;\to \;\mathrm{3Fe}+2{\mathrm{CO}}_{2},\;\Delta H_{298\;K}=333\;\mathrm{kJ}\;{\mathrm{mol}}^{-1}$$

(39) $${\mathrm{CaCO}}_{3}\to \mathrm{CaO}+{\mathrm{CO}}_{2},\;\Delta H_{298\;K}=178\;\mathrm{kJ}\;{\mathrm{mol}}^{-1}$$

**Figure 38:**
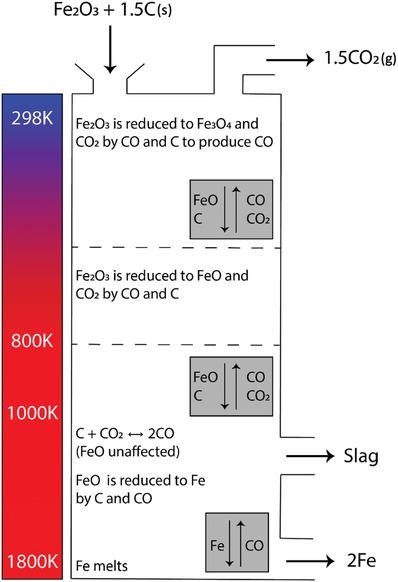
Solar blast furnace.

The visionary work of Steinfeld and Thompson from the early 1990s proposed the combination of these processes with the dry reforming process (see Section 2.1.1) to create a process in which 65% of CO_2_ emissions are reduced through the use of solar energy in place of fossil fuel combustion.^315^ To further this effort, they proposed an idealized concentrated solar blast furnace (**Figure** **38**) that functions as an innovative fractionator that effectively separates CO_2_ and varying iron oxide compounds (FeO, Fe_2_O_3_, etc.).^316^

More recently, a publication from Bender et al. proposed their so‐called “intelligent carbon management” system through the coupled production of steel and chemicals.^317^ In this scheme, MeOH is generated as an intermediate product to promising diesel replacement fuel PODE (mentioned earlier). The direct reduction of iron often uses NG as a reductant (the carbon, C, in the reaction above can be replaced with any number of reductants including H_2_, NG, and CO), which produces preheated syngas that can subsequently be used to synthesize MeOH. Their analysis indicated that this strategy would virtually eliminate all carbon emissions from the existing European steel manufacturing process, and produce usable liquid fuels in the meantime. Process economics for their six carbon reduction scenarios indicated a net additional equipment investment of €2.7–11.4 billion (≈US$3.1–12.9 billion) for 12 MMTA_steel_ production.

## 12 Conclusions and Outlook

This review has provided a comparative investigation of solar‐assisted MeOH processes starting from CO_2_, water, and sunlight, to the TCST process starting from NG and biomass. Through a combination of original work and literature review, it has been demonstrated that shifting to a solar‐assisted CO_2_ and water‐derived CO‐rich or CO_2_‐rich feed could be feasible in a correctly incentivized economy, thereby providing an economically viable GHG utilization strategy to value‐added products, such as MeOH. Various solar‐assisted technologies have been described, including biomass thermochemical, solar‐thermochemical, photothermal, photochemical, PV–electrochemical, and photoelectrochemical systems. All of the aforementioned technologies are currently at different stages of technology readiness levels (TRLs), and most require further research to improve rates, yields, and selectivity to MeOH to become commercially viable. Other factors not addressed here, that also contribute to the economic viability and reduced GHG emission potential of these technologies, include compatibility with existing infrastructure and availability and affordability of land area.

As has been stated, introducing light presents challenges incorporating it into catalyst macroarchitectures and as an additional transport phenomenon that affects the design of photoreactors, and requires careful consideration in order to most efficiently utilize the available light energy. Use of porous films and foams, and their continuing improvement in terms of transport considerations are crucial to development of solar‐assisted chemical processes. Deriving process utility heat and electricity from harnessing solar energy via PVs was also discussed, although it was found that such applications are challenging economically with current PV technology. Finally, MeOH is a promising product compared to other oxygenates due to its flexibility in chemical, energy, and transportation sectors.

Research opportunities in solar‐assisted conversion of CO_2_‐to‐MeOH technologies are vast and extend from catalyst engineering to novel feed processing and reactor design. The discovery of low‐temperature, selective, highly active, solar‐enhanced, scalable, economical, and water‐stable catalysts is a task that requires both detailed theoretical understanding of the surface chemistry and thoughtful engineering. Various CO_2_‐to‐CO preparation technologies are now available, and in the case of RWGS are affordable, thereby allowing continued use of commercial catalyst systems. With current PV technology, carbon taxes of US$500 MT_CO2_^−1^ are necessary for solar fuel adoption decreasing to US$210 MT_CO2_^−1^ with optimistic progress in PV research and development. This is due to the main cost and energy driver for producing MeOH from CO_2_ and water being H_2_ derived from water electrolysis, representing 90% of the MeOH MSP and 80% of the total energy requirement for strategy A.

Though developing rapidly, solar‐assisted CO_2_‐to‐MeOH processes remain, for now, a visionary technology. With further research and development, solar technologies may one day allow us to curb GHG emissions while maintaining sustainable and affordable access to a nonpetroleum‐derived industrial chemical precursor and drop‐in liquid fuel, vital to modern society.

## Conflict of Interest

The authors declare no conflict of interest.
